# Genome Sequence and Metabolic Analysis of a Fluoranthene-Degrading Strain *Pseudomonas aeruginosa* DN1

**DOI:** 10.3389/fmicb.2018.02595

**Published:** 2018-10-31

**Authors:** Chunqiu He, Yanpeng Li, Chao Huang, Fulin Chen, Yanling Ma

**Affiliations:** Shaanxi Provincial Key Laboratory of Biotechnology, Key Laboratory of Resources Biology and Biotechnology in Western China, Ministry of Education, College of Life Science, Northwest University, Xi’an, China

**Keywords:** *Pseudomonas aeruginosa* DN1, genomic sequence analysis, fluoranthene degradation, intermediate metabolites, gene knockout

## Abstract

*Pseudomonas aeruginosa* DN1, isolated from petroleum-contaminated soil, showed excellent degradation ability toward diverse polycyclic aromatic hydrocarbons (PAHs). Many studies have been done to improve its degradation ability. However, the molecular mechanisms of PAHs degradation in DN1 strain are unclear. In this study, the whole genome of DN1 strain was sequenced and analyzed. Its genome contains 6,641,902 bp and encodes 6,684 putative open reading frames (ORFs), which has the largest genome in almost all the comparative *Pseudomonas* strains. Results of gene annotation showed that this strain harbored over 100 candidate genes involved in PAHs degradation, including those encoding 25 dioxygenases, four ring-hydroxylating dioxygenases, five ring-cleaving dioxygenases, and various catabolic enzymes, transcriptional regulators, and transporters in the degradation pathways. In addition, gene knockout experiments revealed that the disruption of some key PAHs degradation genes in DN1 strain, such as *cat*A, *pca*G, *pca*H, and *rhd*A, did not completely inhibit fluoranthene degradation, even though their degradative rate reduced to some extent. Three intermediate metabolites, including 9-hydroxyfluorene, 1-acenaphthenone, and 1, 8-naphthalic anhydride, were identified as the dominating intermediates in presence of 50 μg/mL fluoranthene as the sole carbon source according to gas chromatography mass spectrometry analysis. Taken together, the genomic and metabolic analysis indicated that the fluoranthene degradation by DN1 strain was initiated by dioxygenation at the C-1, 2-, C-2, 3-, and C-7, 8- positions. These results provide new insights into the genomic plasticity and environmental adaptation of DN1 strain.

## Introduction

Polycyclic aromatic hydrocarbons (PAHs) are characterized as hazardous organic pollutants comprising two or more fused benzene rings in linear, angular and clustered arrangements, and their distribution in the environment and possible human exposure is a public concern (Seeger et al., 2010; Kumar et al., 2011). To minimize PAHs threat to human health, increasing interest is given to restore the contaminated sites with PAHs (Kanaly and Harayama, 2000; Yan et al., 2004). Microbial decontamination of PAHs is claimed to be an efficient and economic alternative to physicochemical treatment techniques (Gordon and Dobson, 2001; Fuchedzhieva et al., 2008; Cao et al., 2015). Although great advances have been made in many aspects of PAHs biodegradation, there remain practical constrains in efficiently implementing bioremediation technology due to the complex nature of the contaminated sites and the lack of valuable information concerning the environment adaption and pollutant degradation performance of microbes (Kanaly and Harayama, 2000; Fuchs et al., 2011; Kweon et al., 2011; Cao et al., 2015). Therefore, to develop a bioremediation strategy it is essential to understand the genetic potential of the native microorganisms in the contaminated sites.

A number of microorganisms have been isolated from various environments based on their capability to biodegrade PAHs, especially with high molecular weight (HMW) (Sepic et al., 1998; Rehmann et al., 2001; Xu et al., 2011). Fluoranthene, a representative of HMW PAHs, has received extensive attention. Multiple bacteria capable of fluoranthene degradation have been reported, with this biodegradation initiated by dioxygenation at the C-1,2-, C-2,3-, C-7,8- and C-9,10- carbon positions (Rehmann et al., 2001; Kweon et al., 2007; Cao et al., 2015). As a well-known fluoranthene degrader, *Pseudomonas aeruginosa* exhibits robust metabolic capacity, and has been studied from various aspects, including genomics, transcriptomics, metabonomics, and biotechnological application (Kelley et al., 1993; Rehmann et al., 2001; Van et al., 2003). *Pseudomonas* species can adapt to various environmental conditions due to abundant genetic diversity, however, the knowledge about their genome plasticity remains limited. Implementation of more genome sequencing projects is of great help to understand the genetic basis for versatile metabolic potential and environmental adaption (Feng et al., 2007; Jin et al., 2011; Shetty et al., 2015).

*Pseudomonas aeruginosa* DN1, initially isolated from petroleum-contaminated soil, displayed strong ability to utilize fluoranthene and crude oil as sole carbon source, and much work has been done to improve the degradation performance of this strain in our previous studies (Lu et al., 2015; Ma et al., 2016; Dong et al., 2017). However, the molecular mechanism associated with PAH degradation by this strain is still unclear. In this study, gene prediction and annotation of the complete genome sequence of *P. aeruginosa* DN1 was performed to reveal the metabolic capability and ecological adaptation with the respect to PAH degradation, and a fluoranthene degradation pathway in the strain was proposed by gene knockout experiment and gas chromatography mass spectrometry (GC-MS) analysis of metabolites.

## Materials and Methods

### Bacterial Strain and Culture Media

*Pseudomonas aeruginosa* DN1, isolated from a petroleum-contaminated soil from Changqing Oilfield in Shaanxi Province of China, was grown in 1 L of Luria-Bertani (LB) culture media (5 g/L yeast extract, 10 g/L peptone, and 10 g/L NaCl) at 30°C for 48 h with shaking at 120 rpm. For the analysis of fluoranthene degradation, M9 minimal medium supplemented with 50 μg/mL fluoranthene as the sole carbon source was used as previously described (Lu et al., 2015).

### Genome Annotation and Comparative Genomics

The complete genome sequence of DN1 strain was obtained by whole-genome shotgun sequencing using a combined strategy employing PacBio RSII (Pacific Biosciences, Menlo Park, CA, United States) and the Illumina Miseq paired-end sequencing platform (Illumina, San Diego, CA, United States). The gene prediction, functional annotation and comparison were performed using the tools on the Integrated Microbial Genomes (IMG) server^1^ (Markowitz et al., 2006). The Cluster of Orthologous Groups (COG) of protein sequences were analyzed using the functional category comparison tool of IMG Glimmer 3.0 (Delcher et al., 1999). Genomic islands (GIs) were identified using SeqWord Sniffer^2^ and three programs in the IslandViewer package (Langille and Brinkman, 2009). Transport systems were analyzed by using the Transport Classification Database^3^.

The genome information of the reference *Pseudomonas* strains for comparative analysis are retrieved from Integrated Microbial Genomes & Microbiomes^4^ and the *Pseudomonas* Genome DB^5^. For the identification of paralogous families, the NCBI BLASTclust program was used^6^ (Jeukens et al., 2013).

### Construction of Δ*cat*A, Δ*pca*G, Δ*pca*H, and Δ*rhd*A Mutants

For construction of gene knockout mutants, a SacB-based strategy was employed as previously described (Hoang et al., 1998). To construct the *cat*A null mutant (Δ*cat*A), polymerase chain reactions (PCRs) were performed to amplify sequences upstream and downstream of the targeted deletion gene. The upstream fragment was amplified using the primer pair, pEX-*cat*A-up-S and pEX-*cat*A-up-A, and the downstream fragment was amplified with primer pair, pEX-*cat*A-down-S and pEX-*cat*A-down-A (Supplementary Table S1). The two PCR products were digested and cloned into the *Bam*HI/*Hind*III-digested gene replacement vector pEX18Ap, and the resulting plasmid pEX18Ap-*cat*A was obtained. The plasmids were then electroporated into DN1 strain with selection for carbenicillin resistance. Colonies with both carbenicillin resistance and loss of sucrose (10%) susceptibility were selected on LB agar plates containing 300 μg/mL of carbenicillin and 10% sucrose, which typically indicate a double-crossover event and gene replacement occurrence. The Δ*cat*A mutant was further confirmed by PCR. The *pca*G, *pca*H, and *rhd*A mutant was generated by the similar strategy with *cat*A mutant in DN1 strain.

### Analysis of Intermediate Metabolites Associated With Fluoranthene Degradation

DN1 strain and isogenic mutants were grown in M9 minimal medium supplemented with fluoranthene (50 μg/mL) as the sole carbon source, and cultivated at 30°C with shaking at 200 rpm for 9 days. Metabolites were extracted and processed for analysis as described previously by GC-MS analysis (Kweon et al., 2007).

### Nucleotide Sequence Accession Number

The genome sequence of DN1 strain was submitted to the DDBJ/EMBL/GeneBank database with accession numbers CP017099 (chromosome) and CP018048 (plasmid).

## Results

### General Genome Features and Comparative Analysis

**FIGURE 1:**
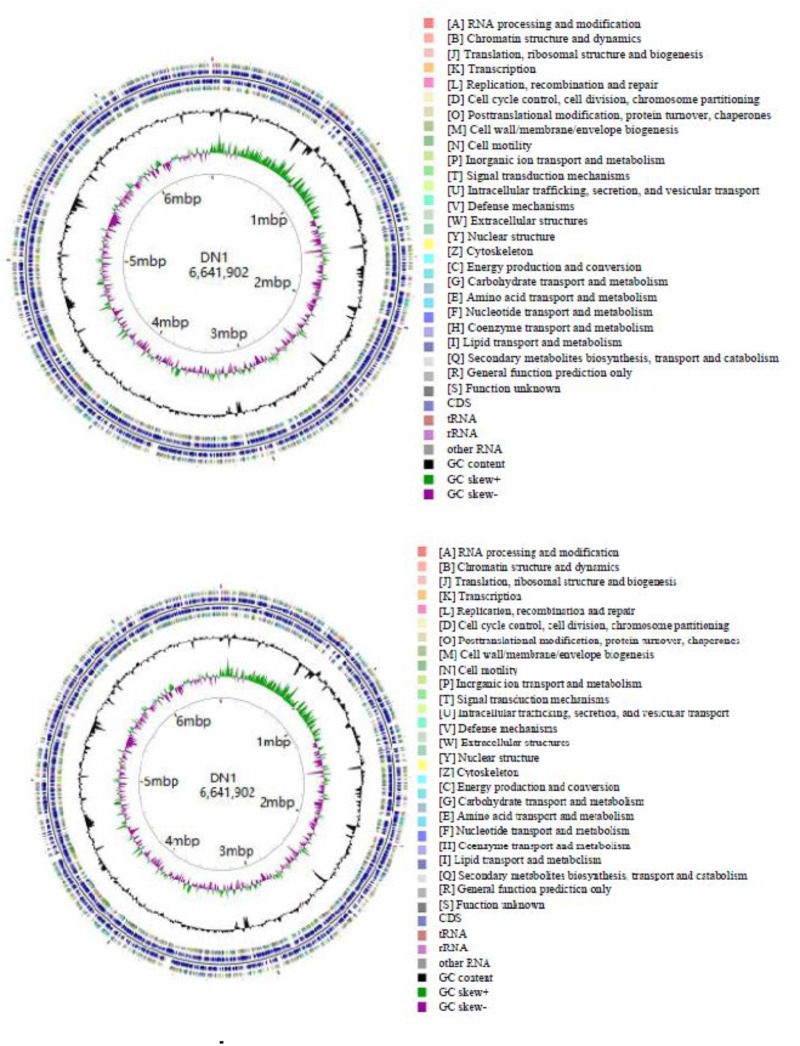
Circular chromosome and plasmid map of *P. aeruginosa* DN1.

As described in our previously report, the genome of *P. aeruginosa* DN1 consisted of a single circular chromosome of 6,641,902,251 base pairs (bp) and a plasmid of 317,349 bp (Dong et al., 2017). Some key structural features of DN1 strain, including GC content, GC skew, and coding sequence (CDS) were graphically depicted in Figure 1. A total of 6,684 open reading frames (ORFs) were predicted, and the deduced proteins were clustered in order to identify paralogs. The entire genome encoded 6,449 candidate protein coding genes (CDSs). Among them, 4,955 (76.8%) CDSs had predicted function based on COG, Pfam and InterPro databases, 496 (7.7%) matched proteins with unknown function, 734 (11.4%) genes had no database matches, and 264 (4.1%) were encoded in the plasmid (Supplementary Tables S2–S8; Dong et al., 2017). In addition, the genome encoded 12 rRNA genes (including 5S rRNA, 16S rRNA and 23S rRNA) and 64 tRNA genes (Table 1).

**Table 1:** Comparative genome analysis of five *P. aeruginosa* strains.

genome features	DN1	PAO1	KF702	N002	DSM50071
Genome size (bp)	6959251	6264404	7164522	6050470	6317050
GC content (%)	66.30%	66.56%	65.81%	66.82%	66.52%
Protein coding genes	6499	5568	6703	5597	5743
Protein coding genes with COGs	4955	4377	4770	4305	4474
RNA genes	154	103	145	137	138
rRNA genes	12	13	3	14	12
5S rRNA	4	4	1	4	4
16S rRNA	4	4	1	4	4
32S rRNA	4	4	1	6	4
tRNA	64	63	61	63	65
other RNAs	78	27	81	60	61


**FIGURE 2:**
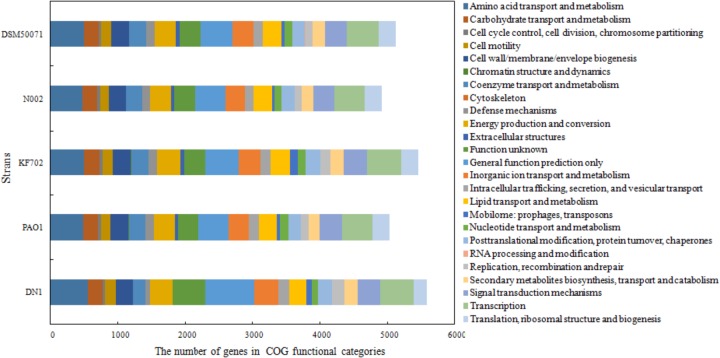
Comparison of COG categories among five *P. aeruginosa* strains.

Some *P.* *aeruginosa* strains have been reported to be associated with PAHs degradation, such as PAO1, KF702, N002, and DSM50071. Here, the genomic comparison between DN1 strain and these strains were performed (Table 1). The results showed the genome of DN1 strain overlapped most extensively with that of the closest relative, *P. aeruginosa* PAO1, and to a high degree with the other *Pseudomonas* members (Dong et al., 2017). However, DN1 strain had a larger genome (6.96 Mb) than PAO1 strain (6.26 Mb), N002 strain (6.05 Mb), and DSM50071 strain (6.32 Mb) (Supplementary Tables S9, S10). Of particular important was genes that distinguished DN1 strain from other strains included those with general prediction and unknown function, apart from the genes involved in amino acid transport and metabolism, cell motility, energy production and conversion, intracellular trafficking, secretion and vesicular transport (Figure 2). Intriguingly, many transcriptional regulator genes in the genome of DN1 strain were predicted to belong to the LysR-type transcriptional regulator family (COG0583, 54 genes), the multiple antibiotic resistance regulator family (COG1846, 11 genes), the isocitrate lyase regulator family, the AraC, GntR, TetR, and FNR families, and two component regulatory systems. Some homologs for these genes were reported to be associated with the pathways involved in aromatic compound degradation (Tropel and van der Meer, 2004; Molina-Henares et al., 2006; Maddocks and Oyston, 2008).

### Genomic Islands and Horizontal Gene Transfer

**FIGURE 3:**
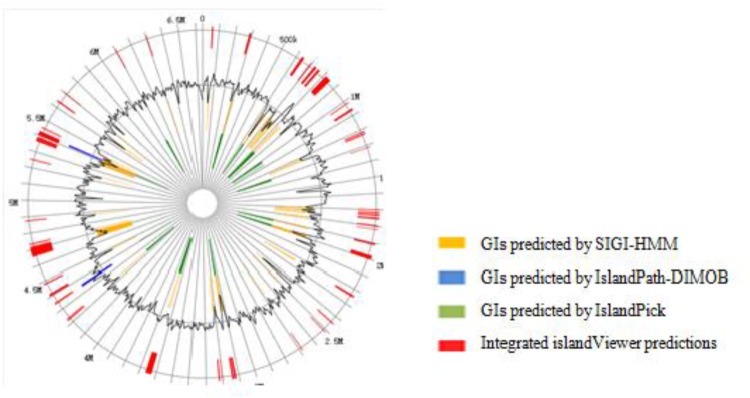
Genomic island prediction of *P. aeruginosa* DN1.

The genomic analysis showed that a number of horizontal gene transfer (HGT) events occurred in DN1 strain. The total number of genes for transposases or relative fragments in the genome of DN1 strain was 83. There were 61 GIs localized in the genome of DN1 strain by IslandPick, IslandPath-DIMOB, and SIGI-HMM methods (Figure 3; Hsiao et al., 2005; Waack et al., 2006; Langille and Brinkman, 2009). These 61 GIs were identified to cover 609,353 bp (9.17%) of the whole chromosome and to comprise 621 genes in total, remarkably contrasting with other group of well-known hydrocarbon utilizing bacteria, such as *Alcanivorax borkumensis*, which has a streamlined genome and a paucity of mobile genetic elements (Binnewies et al., 2006).

Identification and analysis of horizontally transferred GIs revealed identical genes available in other organisms. GIs with probable horizontal origin might play an important role in adapting bacteria to different types of abiotic stress, in addition to conferring antimicrobial resistance and secondary metabolite production. This activity might have been acquired from other organisms or could have evolved after transfer in response to the altered environmental condition. GIs associated with adaptation and environmental interest had substantially impacted bacterial evolution (Hacker and Kaper, 2000; Langille and Brinkman, 2009). In 61 GIs identified here, all genes were among those encoding major facilitator transporters, type II secretion proteins, the hydrogen cyanide synthase HcnB, with these genes involved in adapting to hydrocarbon degradation and biosurfactant synthesis, suggesting that DN1 strain might have used gene transfer to enhance hydrocarbon degradation ability (Supplementary Table S11).

### Biosurfactant Synthesis and PAHs Bioavailability

Hydrophobic PAHs are less available to environmental microbes, and emulsification can help bacteria to solubilize and assimilate PAHs. Biosurfactants, including glycolipids, lipopeptides and lipoproteins, phospholipids, and fatty acids, can emulsify and solubilize hydrocarbons to promote their bioavailability (Kuyukina and Ivshina, 2010). Our previous studies have shown that DN1 strain could produce rhamnolipids, a class of biosurfactants that has represented a suitable alternative to other chemically synthesized counterparts (Lu et al., 2015; Ma et al., 2016; Dong et al., 2017). Many genes involved in biosurfactant rhamnolipid synthesis and regulation have been detected in the genome of DN1 strain (Supplementary Table S12). For example, RmlA, RmlB, RmlC, RmlD, RhlA, RhlB, and RhlC were the key enzymes for rhamnolipid production. The RhlR/C_4_-HSL complex activates the *rhl*A promoter independently from σ_54_ at the transcriptional level, as it remained unclear if the latter only acts indirectly on *rhl*AB transcription. Additionally MvfR and PtxR were the transcriptional regulators, while AlgR was the response regulator of the LytR/AlgR family, and RsmA acted as carbon storage regulator (*csr*A).

In addition, many genes involved in flagella assembly, cell motility, and chemotaxis were identified based on Kyoto Encyclopedia of Genes and Genomes analysis. These included genes predicted to encode flagellin, flagellar motor protein, and chemotaxis complex proteins, such as *flg*A-H, M, *fli*A, E-H, J, M, S, and c*he*A, B, D, R, W, Y, which could actively increase the PAH bioavailability and help bacteria translocate and attach the niches with high content of hydrocarbons (Supplementary Table S3; Velasco-Casal et al., 2008; Nie et al., 2012).

### Genes Associated With PAHs Degradation

The genome of DN1 strain carried more than 100 candidate genes potentially involved in the metabolism of aromatic hydrocarbons, and most of the predicted catabolic genes were located in six regions, including region I, position 181,326–278,269 bp, region II, position 919,361–1,059,085 bp, region III, position 1,570,759–1,917,246 bp, region IV, position 2,284,846–2,955,492 bp, region V, position 3,474,080–3,970,175 bp, and region VI, position 4,705,323–6,485,022 bp (Supplementary Table S13). Other genes involved in the metabolism of aromatic hydrocarbons were found dispersedly throughout the genome.

**FIGURE 4:**
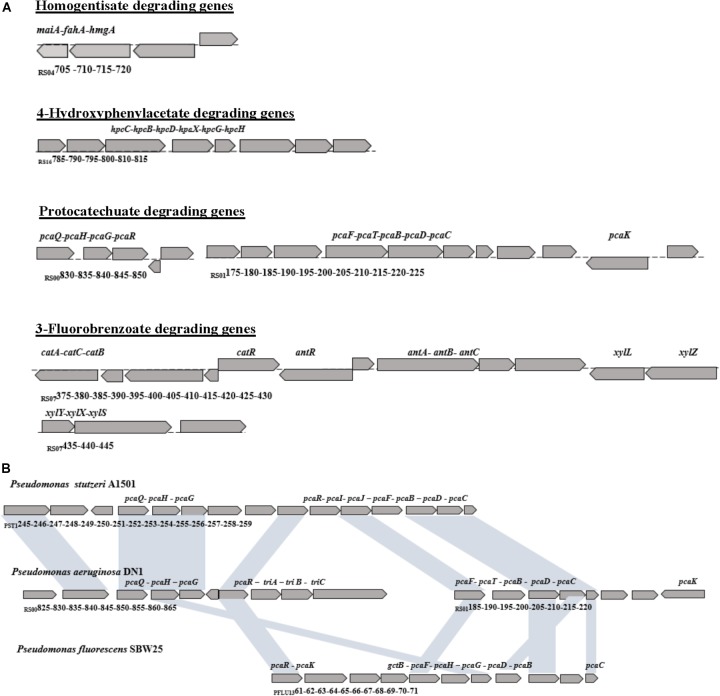
PAHs degrading genes of *P. aeruginosa* DN1. **(A)** PAHs degrading gene loci, **(B)** The gene clusters for protocatechuate degradation pathway in *Pseudomonas.*

Within a 96.94 kp area of region I, the genes involved in the protocatechuate pathway related to PAHs biodegradation were identified (Figure 4A). Among these, *pca*G (RS_00840, protocatechuate 3,4-dioxygenase subunit alpha) was predicted to encode a ring-hydroxylating dioxygenase involved in protocatechuate degradation via the β-ketoadipate pathway. Downstream genes, such as *pca*B (RS_01185, 3-carboxy-cis, *cis*-muconate cycloisomerase) and *pca*C (RS_01195, gamma-carboxymu-conolactone decarboxylase), were predicted as responsible for the next several steps of protocatechuate degradation. In addition, region III contained the genes predicted to be related to catechol degradation, including *cat*A (RS_07375, catechol 1,2-dioxygenase), *cat*B (RS_07385, muconate cycloisomerase I), and *cat*C (RS_07380, muconolactone delta-isomerase), and involved in converting catechol to 2-oxo-2,3-dihydrofuran-5-acetate, which was further metabolized to succinyl-CoA as an intermediate in the TCA cycle (Supplementary Table S14). Moreover, the genes involved in the 3-fluorobenzoate and benzoate pathways were also identified in this region, including *benA-xyl*X (RS_07440, benzoate/toluate 1,2-dioxygenase subunit alpha), *benB-xyl*Y (RS_07435, benzoate/toluate 1,2-dioxygenase subunit beta), *benC-xyl*Z (RS_07430, benzoate/toluate1,2-dioxygenase reductase component), and *benD-xyl*L (RS_07425, dihydroxycyclo hexadienecarboxylate dehydrogenase). Region V contained the genes involved in 4-hydroxyphenylacetate metabolism, including *hpa*D/*hpc*B (RS_16790, 3,4-dihydroxyphenylacetate 2,3-dioxygenase), *hpa*E/*hpc*C (RS_16785, 5-carboxy- methyl-2-hydroxymuconic-semialdehyde dehydrogenase), *hpa*F/*hpc*D (RS_16795, 5-carboxymethyl-2-hydroxylmuconate isomerase), *hpa*G (RS_16780, 5-oxopent -3-ene-1,2,5-tricarboxylate decarboxylase), *hpa*H (RS_16810, 2-oxo-hept-3-ene -1,7-dioate hydratase), and *hpa*I/*hpc*H (RS_16815, 4-hydroxy-2-oxoheptanedioate aldolase). Three sets of genes involved in the gentisate degradation pathway were found in the chromosome genome of strain DN1, including RS_07190 and RS_22530 (gentisate 1,2-dioxygenase), RS_04705 (maleylacetoacetate isomerase), and RS_04710 (fumarylpyruvate hydrolase) in region V and VI, whereas homogentisate degrading genes were found in region II of DN1 strain chromosome.

Compared with the corresponding genes from *P. stutzeri* A1501 and *P. fluorescens* SBW25, the orthologous genes within region I involved in the protocatechuate pathway related to PAH degradation were identified in DN1 strain (Figure 4B). The gene clusters located in region I from RS_00825 to RS_01220 (177,614–263,819 bp) in DN1 strain were likely discontinuous, such as *pca*Q*, gdtA, pca*G, *pca*B*, pca*D, and *pca*C, which differed greatly from the two strains with the successive gene clusters located from PST_1245 to PST_1259 (1,354,724–1,370,743 bp) and PFLU_1361 to PFLU_1371 (1,501,030–1,511,851 bp), respectively.

Additionally, there were 70 genes between *pca*R and *pca*F in the non-consecutive region of DN1 genome, with most genes found to encode proteins, such as TriA TriB and TriC, belonging to the RND family related to the transport of iron ions. This region also included genes encoding oxaloacetic acid decarboxylase, which participates in the TCA cycle and an aromatic sulfate enzyme. Apart from the positional variance of *pca*D and *gdtA* in *P. fluorescens* SBW25, the arrangement of key genes remained consistent among the nine homologous sequences between *P. aeruginosa* DN1 and *P. stutzeri* A1501.

### Fluoranthene Degradation

**FIGURE 5:**
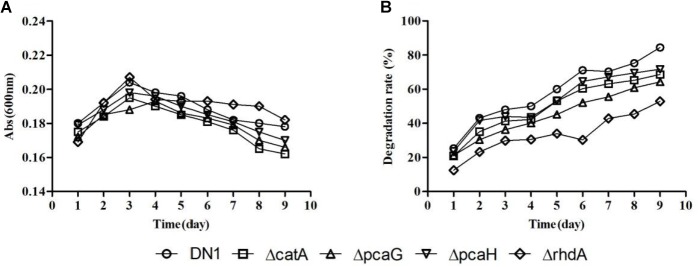
Growth **(A)** and degradative **(B)** curves of *P. aeruginosa* DN1 wild-type strain and isogenic mutants when grown in M9 medium supplemented with 50 μg/mL fluoranthene.

DN1 strain was shown to be able to degrade fluoranthene and crude oil in our previous studies (Lu et al., 2015; Ma et al., 2016). Based on the functional analysis of genome, the potential metabolic pathways associated with PAHs along with the most probable genes to each enzymatic reaction were theoretically predicted as shown in Supplementary Figure S1 (Kelley et al., 1993; Rehmann et al., 2001; Kweon et al., 2007; Cao et al., 2015). PAHs are frequently catabolized through pathways associated with oxidative metabolism beginning with ring-hydroxylating and ring-cleaving dioxygenase reaction that are critical de-aromatization steps in PAH degradation (Rehmann et al., 2001; Kweon et al., 2007). When the gene *rhd*A (RS_30940) that is predicted to encode the aromatic ring dioxygenase alpha submit from DN1 strain was disputed, the mutant Δ*rhd*A still remained the ability to degrade fluoranthene in spite of a degradation rate reduction from 84.47% (wild-type) to 52.89% (Δ*rhd*A) when 50 μg/mL fluoranthene was used as the sole carbon source, indicating *rhd*A was not the only one gene encoded the dioxygenase subunit. The disruption of other genes, such as Δ*cat*A, Δ*pca*G, Δ*pca*H, had little impact on the metabolism of fluoranthene, but their degradative rates were also lower than that of the wild-type strain (Figure 5). These results demonstrated that deletion of genes encoding specific enzymes involved in PAHs biodegradation was insufficient to completely inhibit fluoranthene degradation.

**FIGURE 6:**
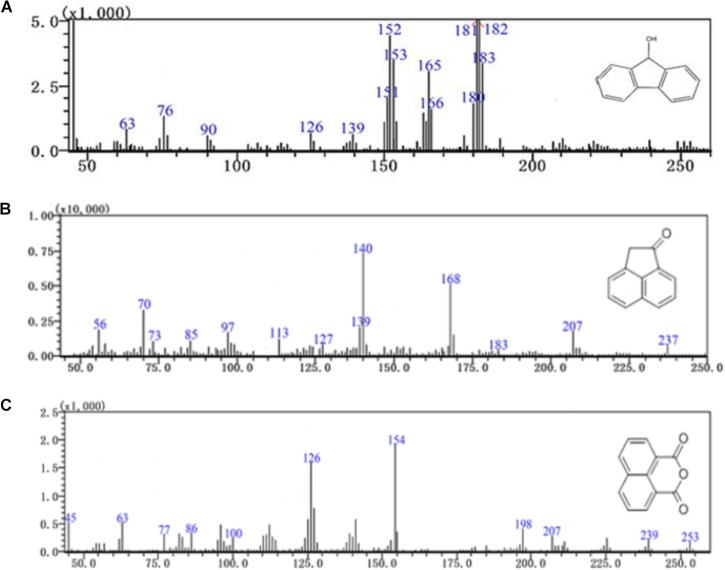
GC-MS profile of fluoranthene metabolite **(A)** 9-hydroxyfluorene, **(B)** 1-acenaphthenone, **(C)** 1,8-naphthalic anhydride.

Furthermore, three major metabolites of fluoranthene degradation, including 9-hydroxyfluorene (I), 1-acenaphthenone (II) and 1,8-naphthalic anhydride (III), were detected by GC-MS analysis during fluoranthene metabolism (Figure 6). Previous studies reported that formation of 9-hydroxyfluorene indicates an initial attack on the fused aromatic ring potion of fluoranthene, likely mediated by a dioxygenase at positions 1 and 2 and in the presence of a dihydroxylated fluoranthene intermediate. Additionally, formation of 1-acenaphthenone and 1,8-naphthalic anhydride suggests that dihydroxylation of the single benzene ring on the fluoranthene molecule occurred at positions 7- and 8 (Kelley et al., 1993; Rehmann et al., 2001; Kweon et al., 2007; Cao et al., 2015).

## Discussion

Genome sequencing of DN1 strain was conducted to expand the knowledge how this bacterium metabolizes PAHs. The genome of DN1 strain harbors over 100 candidate genes potentially involved in PAH metabolism, as well as 198 genes associated with two-component systems, including genes encoding 76 histidine kinases and response regulators. The presence of stress-response and transporter genes provides DN1 strain an advantage for survival in PAH-heavy environments. Compared with some other *P. aeruginosa* strains associated with PAH degradation, such as PAO1, KF702, N002, and DSM50071, the genome of DN1 strain possesses additional genes with generally predicted and unknown function, which indicated that we can further explore new function genes by pan-genome analysis in the next work. Moreover, the DN1 genome contains larger number of GIs and transposases than *Celeribacter indicus* P73^T^, enabling DN1 adaption to different levels of abiotic stress and the conference of antimicrobial resistance (Cao et al., 2015).

Aromatic ring hydroxylation is the most difficult catalytic step during PAH degradation. Although fluoranthene degradation was found to be initiated by dioxygenation at the C-1,2-, C-2,3-, C-7,8-, and C-8,9- positions, and a possible monooxygenation was used to produce monohydroxy fluoranthene according to previous reports. Taken together, the different catabolic genes and regulation and transportation mechanisms in different strains highlight the different characteristics of fluoranthene degradation (Feng et al., 2007; Shetty et al., 2015). For example, Rehmann et al. (2001) indicated that fluoranthene was started with a dioxygenation at C-2,3 position in *Mycobacterium sp.* KR20 strain, while dioxygenation was reported to start at least at the C-1,2, C-2,3 and C-7,8 positions in *Mycobacterium vanbaalenii* PYR-1 (Kweon et al., 2007).

**FIGURE 7:**
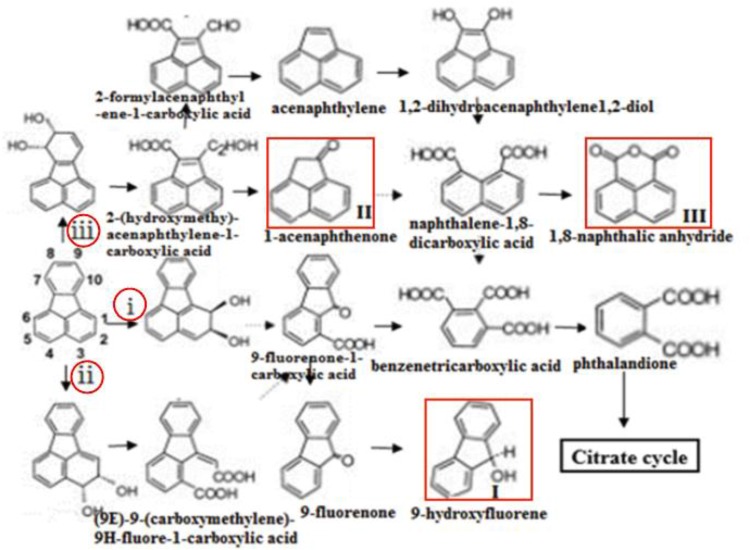
Proposed pathway of fluoranthene biodegradation by *P. aeruginosa* DN1. Compounds in red frame were identified. i, ii, iii shows that the dioxygenation degradation pathways of fluoranthene were initiated at the C-1,2, C-2,3, C-7,8 positions, respectively.

In this study, the gene knockout experiments revealed that the disruption of some key genes in DN1 strain could not completely inhibit the degradation of fluoranthene. Taken this with the GC-MS analysis result that 9-hydroxyfluorene, 1-acenaphthenone, 1,8-naphthalic anhydride were detected, we speculated that fluoranthene degradation was initiated in DN1 strain by ring-hydroxylating dioxygenase reactions at the C-1,2- and/or C-2,3- positions in order to form fluoranthene *cis*-1,2-dihydrodiol and/or fluoranthene *cis*-2,3-dihydrodiol, immediately followed by dehydrogenation to produce 1,2-dihydroxyfluoranthene or 2,3-dihydroxyfluoranthene, which was subjected to extradiol ring rupture by an extradiol-type ring-cleaving dioxygenase to form (9E)-9-(carboxymethylene)-9H-fluore-1-carboxylic acid or 9-fluorenone-1-carboxylic acid. Subsequently, the product of the final step might be converted to 9-fluorenone-1-carboxylic acid, followed by further reduction to 9-fluorenone and 9-hydroxyfluorene (I). Furthermore, fluoranthene also underwent a reaction catalyzed by ring-hydroxylating dioxygenases at the C-7,8- position in order to form fluoranthene *cis*-7,8-dihydrodiol, with the resulting fluoranthene *cis*-7,8-dihydrodiol catalyzed by dihydrodiol dehydrogenase to produce 7,8-dihydroxyfluoranthene, followed by extradiol ring rupture by a ring-cleaving dioxygenase to form (2Z,4Z)-2-hydroxy-4-(2-oxoacenaphthylen-1 (2H)-ylidene) but-2-enoic acid. Subsequent steps were catalyzed by hydratase-aldolase to form 2-hydroxy-acenaphthylene-1-carbaldehyde, after which the central metabolite 1-acenaphthenone (II) was reduced to naphthalene-1, 8-dicarboxylic acid. Besides, 1,8-naphthalic anhydride(III) was subsequently converted from naphthalene-1, 8-dicarboxylic acid through intracellular pyrolysis by microorganisms (Figure 7). These proposed metabolic pathways involved in fluoranthene degradation by *P. aeruginosa* DN1 and the most probable genes associated with each enzymatic reaction require further in-depth exploration and confirmation in future studies.

## Author Contributions

FC and YM conceived and designed the study. CHe, YL, and CHu performed the experiments and bioinformatics analyses. YL and CHe contributed to materials and analysis tools. CHe, FC, and YM wrote the paper. All authors read and approved the final manuscript.

## Conflict of Interest Statement

The authors declare that the research was conducted in the absence of any commercial or financial relationships that could be construed as a potential conflict of interest.

## Footnotes

1 https://img.jgj.doe.gov

2 http://genomics1.mhhannover.de/seqword/sniffer/index.html

3 http://www.tcdb.org

4 http://img.jgi.doe.gov

5 http://www.pseudomonas.com

6 https://blast.ncbi.nlm.nih.gov/Blast.cgi

## References

[B1] Binnewies T. T. Motro Y. Hallin P. F. Lund O. Dunn D. La T. (2006). Ten years of bacterial genome sequencing: comparative-genomics-based discoveries. *Funct. Integr. Genomics* 6 165–185. 10.1007/s10142-006-0027-2 16773396

[B2] Cao J. Lai Q. Yuan J. Shao Z. (2015). Genomic and metabolic analysis of fluoranthene degradation pathway in *Celeribacter indicus* P73(T.). *Sci. Rep.* 5:7741. 10.1038/srep07741 25582347 PMC4291564

[B3] Delcher A. L. Harmon D. Kasif S. White O. Salzberg S. L. (1999). Improved microbial gene identification with GLIMMER. *Nucleic Acids Res.* 27 4636–4641. 10.1093/nar/27.23.4636 10556321 PMC148753

[B4] Dong W. He C. Li Y. Huang C. Chen F. Ma Y. (2017). Complete genome sequence of a versatile hydrocarbon degrader, *Pseudomonas aeruginosa* DN1 isolated from petroleum-contaminated soil. *Gene Rep.* 7 123–126. 10.1016/j.genrep.2017.04.001

[B5] Feng L. Wang W. Cheng J. Ren Y. Zhao G. Gao C. (2007). Genome and proteome of long-chain alkane degrading *Geobacillus thermodenitrificans* NG80-2 isolated from a deep-subsurface oil reservoir. *Proc. Natl. Acad. Sci. U.S.A.* 104 5602–5607. 10.1073/pnas.0609650104 17372208 PMC1838512

[B6] Fuchedzhieva N. Karakashev D. Angelidaki I. (2008). Anaerobic biodegradation of fluoranthene under methanogenic conditions in presence of surface-active compounds. *J. Hazard. Mater.* 153:123. 10.1016/j.jhazmat.2007.08.027 17869417

[B7] Fuchs G. Boll M. Heider J. (2011). Microbial degradation of aromatic compounds - from one strategy to four. *Nat. Rev. Microbiol.* 9:803. 10.1038/nrmicro2652 21963803

[B8] Gordon L. Dobson A. D. W. (2001). Fluoranthene degradation in *Pseudomonas* alcaligenes PA-10. *Biodegradation* 12 393–400. 10.1023/A:1015029519142 12051645

[B9] Hacker J. Kaper J. B. (2000). Pathogenicity islands and the evolution of microbes. *Annu. Rev. Microbiol.* 54:641 10.1146/annurev.micro.54.1.641 11018140

[B10] Hoang T. T. Karkhoff-Schweizer R. R. Kutchma A. J. Schweizer H. P. (1998). A broad-host-range Flp-FRT recombination system for site-specific excision of chromosomally-located DNA sequences: application for isolation of unmarked *Pseudomonas aeruginosa* mutants. *Gene* 212 77–86. 10.1016/S0378-1119(98)00130-9 9661666

[B11] Hsiao W. W. Ung K. Aeschliman D. Bryan J. Finlay B. B. Brinkman F. S. (2005). Evidence of a large novel gene pool associated with prokaryotic genomic islands. *PLoS Genetics* 1:e62. 10.1371/journal.pgen.0010062 16299586 PMC1285063

[B12] Jeukens J. Boyle B. Bianconi I. Kukavica-Ibrulj I. Tümmler B. Bragonzi A. (2013). Complete genome sequence of persistent cystic fibrosis Isolate *Pseudomonas aeruginosa* strain RP73. *Genome Announc.* 1:e00568-13. 10.1128/genomeA.00568-13 23908295 PMC3731849

[B13] Jin H. M. Jeong H. Moon E. J. Math R. K. Lee K. Kim H. J. (2011). Complete genome sequence of the polycyclic aromatic hydrocarbon-degrading bacterium *Alteromonas* sp. *strain SN*2. *J. Bacteriol.* 193 4292–4293. 10.1128/JB.05252-11 21705606 PMC3147666

[B14] Kanaly R. A. Harayama S. (2000). Biodegradation of high-molecular-weight polycyclic aromatic hydrocarbons by bacteria. *J. Bacteriol.* 182 2059–2067. 10.1128/JB.182.8.2059-2067.2000 10735846 PMC111252

[B15] Kelley I. Freeman J. P. Evans F. E. Cerniglia C. E. (1993). Identification of metabolites from the degradation of fluoranthene by Mycobacterium sp. strain PYR-1. *Appl. Environ. Microbiol.* 59 800–806. 8481006 10.1128/aem.59.3.800-806.1993 PMC202192

[B16] Kumar S. Upadhayay S. K. Kumari B. Tiwari S. Singh S. N. Singh P. K. (2011). In vitro degradation of fluoranthene by bacteria isolated from petroleum sludge. *Bioresour. Technol.* 102 3709–3715. 10.1016/j.biortech.2010.11.101 21177104

[B17] Kuyukina M. S. Ivshina I. B. (2010). *Rhodococcus biosurfactants*: biosynthesis, properties, and potential applications. *Biol. Rhodococcus* 16 291–313.

[B18] Kweon O. Kim S. J. Holland R. D. Chen H. Kim D. W. Gao Y. (2011). Polycyclic aromatic hydrocarbon metabolic network in *Mycobacterium vanbaalenii* PYR-1. *J. Bacteriol.* 193:4326. 10.1128/JB.00215-11 21725022 PMC3165511

[B19] Kweon O. Kim S. J. Jones R. C. Freeman J. P. Adjei M. D. Edmondson R. D. (2007). A polyomic approach to elucidate the fluoranthene-degradative pathway in *Mycobacterium vanbaalenii* PYR-1. *J. Bacteriol.* 189 4635–4647. 10.1128/JB.00128-07 17449607 PMC1913438

[B20] Langille M. G. Brinkman F. S. (2009). Island Viewer: an integrated interface for computational identification and visualization of genomic islands. *Bioinformatics* 25 664–665. 10.1093/bioinformatics/btp030 19151094 PMC2647836

[B21] Lu W. Luo N. Dong W. Ma Y. L. (2015). Identification and characterization of a *Pseudomonas aeruginosa* strain DN1 in fluoranthene biodegradation. *Acta Scientiae Circumstantiae* 35 3486–3492.

[B22] Ma K. Y. Sun M. Y. Dong W. He C. Q. Chen F. L. Ma Y. L. (2016). Effects of nutrition optimization strategy on rhamnolipid production in a *Pseudomonas aeruginosa* strain DN1 for bioremediation of crude oil. *Biocatal. Agric. Biotechnol.* 6 144–151. 10.1016/j.bcab.2016.03.008

[B23] Maddocks S. E. Oyston P. C. (2008). Structure and function of the LysR-type transcriptional regulator (LTTR) family proteins. *Microbiology* 154 3609–3623. 10.1099/mic.0.2008/022772-0 19047729

[B24] Markowitz V. M. Frank K. Krishna P. Ernest S. Greg W. Anu P. (2006). The integrated microbial genomes (IMG) system. *Nucleic Acids Res.* 34 344–348. 10.1093/nar/gkj024 16381883 PMC1347387

[B25] Molina-Henares A. Krell T. Guazzaroni M. Segura A. Ramos J. L. (2006). Members of the IcIR family of bacterial transcriptional regulators function as activators and/or repressors. *FEMS Microbiol. Rev.* 30:157. 10.1111/j.1574-6976.2005.00008.x 16472303

[B26] Nie Y. Tang Y. Q. Li Y. Chi C. Q. Cai M. Wu X. L. (2012). The genome sequence of polymorphum gilvum SL003B-26A1(T) reveals its genetic basis for crude oil degradation and adaptation to the saline soil. *PLoS One* 7:e31261. 10.1371/journal.pone.0031261 22359583 PMC3281065

[B27] Rehmann K. Hertkorn N. Kettrup A. A. (2001). Fluoranthene metabolism in *Mycobacterium* sp. *strain KR*20: identity of pathway intermediates during degradation and growth. *Microbiology* 147 2783–2794. 10.1099/00221287-147-10-2783 11577157

[B28] Seeger M. Hernández M. Méndez V. Ponce B. Córdova M. González M. (2010). Bacterial degradation and bioremediation of chlorinated herbicides and biphenyls. *J. Soil Sci. Plant Nutr.* 10 320–332. 10.4067/S0718-95162010000100007

[B29] Sepic E. Bricelj M. Leskovsek H. (1998). Degradation of fluoranthene by *Pasteurella* sp. IFA and *Mycobacterium* sp. PYR-1:isolation and identification of metabolites. *J. Appl. Microbiol.* 85 746–754. 10.1111/j.1365-2672.1998.00587.x 9812386

[B30] Shetty A. R. Gannes V. D. Obi C. C. Lucas S. Lapidus A. Cheng J. F. (2015). Complete genome sequence of the phenanthrene-degrading soil bacterium *Delftia acidovorans* Cs1-4. *Stand. Genomic Sci.* 10 1–10. 10.1186/s40793-015-0041-x 26380642 PMC4572682

[B31] Tropel D. van der Meer J. R. (2004). Bacterial transcriptional regulators for degradation pathways of aromatic compounds. *Microbiol. Mol. Biol. Rev. MMBR* 68:474. 10.1128/MMBR.68.3.474-500.2004 15353566 PMC515250

[B32] Van H. R. Wattiau P. Bastiaens L. Daal L. Jonker L. Springael D. (2003). Elucidation of the metabolic pathway of fluorene and cometabolic pathways of phenanthrene, fluoranthene, anthracene and dibenzothiophene by *Sphingomonas* sp. LB126. *Res. Microbiol.* 154:199. 10.1016/S0923-2508(03)00039-1 12706509

[B33] Velasco-Casal P. Wick L. Y. Ortega-Calvo J. J. (2008). Chemo effectors decrease the deposition of chemotactic bacteria during transport in porous media. *Environ. Sci. Technol.* 42 1131–1137. 10.1021/es071707p 18351083

[B34] Waack S. Keller O. Asper R. Brodag T. Damm C. Fricke W. F. (2006). Score-based prediction of genomic islands in prokaryotic genomes using hidden Markov models. *BMC Bioinformatics* 7:142. 10.1186/1471-2105-7-142 16542435 PMC1489950

[B35] Xu H. X. Wu H. Y. Qiu Y. P. Shi X. Q. He G. H. Zhang J. F. (2011). Degradation of fluoranthene by a newly isolated strain of *Herbaspirillum chlorophenolicum* from activated sludge. *Biodegradation* 22 335–345. 10.1007/s10532-010-9403-7 20711747

[B36] Yan J. Wang L. Fu P. P. Yu H. (2004). Photomutagenicity of 16 polycyclic aromatic hydrocarbons from the US EPA priority pollutant list. *Mutat. Res.* 557 99–108. 10.1016/j.mrgentox.2003.10.004 14706522 PMC2713671

