# Supplementary material for: Genome Sequence and Metabolic Analysis of a Fluoranthene-Degrading Strain Pseudomonas aeruginosa DN1
Source: Front Microbiol. 2018 Oct 31;9:2595. doi: 10.3389/fmicb.2018.02595 (PMC6220107; doi:10.3389/fmicb.2018.02595)
Supplement: Supplementary file 1 [file Table_1.DOCX]

**Table S1 |The primers used in this study**

| Primers | Sequences（5’ to 3’） | Application |
| --- | --- | --- |
| *catA*-up-S | AATggatccCTGGTTGGTCAGCGGGTTG |  |
| *catA*-up-A | ACGtctagaGCGGGCATTGCAGGAGTA |  |
| *catA*-down-S | CCGtctagaCGGCAGTCTGGGAAATCTTCA |  |
| *catA*-down-A | CCGaagcttAGAATGAGCCAAGCCGTGATC | Constructing *catA* mutant |
| *catA* Sense | TTGCCATGAAGATCATCCCG |  |
| *catA* Antiense | GAAGCCATTCCGCAGACAGA |  |
| *pcaG*- up-S | TACggatccTCAGGGAGAAGGACGCGG |  |
| *pcaG*- up-A | TAAtctagaCCAGGCCGATGTGGACGT |  |
| *pcaG*- down-S | TAAtctagaTGCGAAGTGGACGGACAG |  |
| *pcaG*- down-A | TCTaagcttCGTCGACATCGTGGCTGA | Constructing *pcaG* mutant |
| *pcaG* Sense | GCGAGGGCCACTATTACTTCC |  |
| *pcaG* Antiense | GATCTGGGCGATGGTCAGG |  |
| *pcaH*- up-S | TACggatccCTGGCGCTGTTCGACATC |  |
| *pcaH*- up-A | TAAtctagaCGGCACGGAGGTCTTGTA |  |
| *pcaH*- down-S | TAAtctagaACATGAGCATGGCCAACC |  |
| *pcaH*- down-A | TCTaagcttCCAGCGACCTTTGGAATG | Constructing *pcaH* mutant |
| *pcaH* Sense | GACGTCGGCAATGGCGAG |  |
| *pcaH* Antiense | GTTGAACGGTCGCTCCGG |  |
