# Supplementary material for: Genome Sequence and Metabolic Analysis of a Fluoranthene-Degrading Strain Pseudomonas aeruginosa DN1
Source: Front Microbiol. 2018 Oct 31;9:2595. doi: 10.3389/fmicb.2018.02595 (PMC6220107; doi:10.3389/fmicb.2018.02595)
Supplement: Supplementary file 2 [file Table_2.DOCX]

**Table S2 | Amino acid transport and metabolism**

| **Locus Tag** | **Gene Product Name** | **Function ID** |
| --- | --- | --- |
| DN1_orf00007 | Histidinol phosphatase and related phosphatases | COG0241 |
| DN1_orf00032 | Shikimate 5-dehydrogenase | COG0169 |
| DN1_orf00039 | ABC-type proline/glycine betaine transport systems, periplasmic components | COG2113 |
| DN1_orf00047 | Tryptophan synthase beta chain | COG0133 |
| DN1_orf00049 | Tryptophan synthase alpha chain | COG0159 |
| DN1_orf00075 | Asparagine synthase (glutamine-hydrolyzing) | COG0367 |
| DN1_orf00094 | Zn-dependent oligopeptidases | COG0339 |
| DN1_orf00188 | Gamma-aminobutyrate permease and related permeases | COG1113 |
| DN1_orf00240 | Gamma-glutamyltransferase | COG0405 |
| DN1_orf00316 | Amino acid transporters | COG0531 |
| DN1_orf00320 | Spermidine/putrescine-binding periplasmic protein | COG0687 |
| DN1_orf00322 | Dihydrodipicolinate synthase/N-acetylneuraminate lyase | COG0329 |
| DN1_orf00329 | Permeases of the major facilitator superfamily | COG0477 |
| DN1_orf00338 | Permeases of the major facilitator superfamily | COG0477 |
| DN1_orf00345 | Permeases of the drug/metabolite transporter (DMT) superfamily | COG0697 |
| DN1_orf00348 | Permeases of the major facilitator superfamily | COG0477 |
| DN1_orf00352 | 3-dehydroquinate dehydratase II | COG0757 |
| DN1_orf00354 | Shikimate 5-dehydrogenase | COG0169 |
| DN1_orf00356 | Permeases of the major facilitator superfamily | COG0477 |
| DN1_orf00391 | 4-aminobutyrate aminotransferase and related aminotransferases | COG0160 |
| DN1_orf00395 | Transcriptional regulators containing a DNA-binding HTH domain and an aminotransferase domain (MocR family) and their eukaryotic orthologs | COG1167 |
| DN1_orf00427 | Na+/proline symporter | COG0591 |
| DN1_orf00429 | Arginase/agmatinase/formimionoglutamate hydrolase, arginase family | COG0010 |
| DN1_orf00436 | Peptidylarginine deiminase and related enzymes | COG2957 |
| DN1_orf00440 | Spermidine/putrescine-binding periplasmic protein | COG0687 |
| DN1_orf00441 | Glutamine synthetase | COG0174 |
| DN1_orf00443 | Glutamine synthetase | COG0174 |
| DN1_orf00447 | Spermidine/putrescine-binding periplasmic protein | COG0687 |
| DN1_orf00449 | Spermidine/putrescine-binding periplasmic protein | COG0687 |
| DN1_orf00450 | ABC-type spermidine/putrescine transport systems, ATPase components | COG3842 |
| DN1_orf00451 | ABC-type spermidine/putrescine transport system, permease component I | COG1176 |
| DN1_orf00452 | ABC-type spermidine/putrescine transport system, permease component II | COG1177 |
| DN1_orf00466 | ABC-type amino acid transport system, permease component | COG0765 |
| DN1_orf00467 | ABC-type amino acid transport/signal transduction systems, periplasmic component/domain | COG0834 |
| DN1_orf00469 | Phosphoglycerate dehydrogenase and related dehydrogenases | COG0111 |
| DN1_orf00480 | Amino acid transporters | COG0531 |
| DN1_orf00481 | Spermidine/putrescine-binding periplasmic protein | COG0687 |
| DN1_orf00482 | ABC-type spermidine/putrescine transport system, permease component II | COG1177 |
| DN1_orf00483 | ABC-type spermidine/putrescine transport system, permease component I | COG1176 |
| DN1_orf00484 | ABC-type spermidine/putrescine transport systems, ATPase components | COG3842 |
| DN1_orf00492 | Threonine dehydratase | COG1171 |
| DN1_orf00499 | Permeases of the major facilitator superfamily | COG0477 |
| DN1_orf00500 | Phosphoserine phosphatase | COG0560 |
| DN1_orf00526 | Dihydroxyacid dehydratase/phosphogluconate dehydratase | COG0129 |
| DN1_orf00537 | Gamma-glutamyltransferase | COG0405 |
| DN1_orf00540 | Choline dehydrogenase and related flavoproteins | COG2303 |
| DN1_orf00575 | Homoserine acetyltransferase | COG2021 |
| DN1_orf00583 | Pyrroline-5-carboxylate reductase | COG0345 |
| DN1_orf00590 | Cysteine synthase | COG0031 |
| DN1_orf00592 | Cystathionine beta-lyases/cystathionine gamma-synthases | COG0626 |
| DN1_orf00616 | Putative threonine efflux protein | COG1280 |
| DN1_orf00622 | Monoamine oxidase | COG1231 |
| DN1_orf00634 | 5,10-methylenetetrahydrofolate reductase | COG0685 |
| DN1_orf00652 | NADPH-dependent glutamate synthase beta chain and related oxidoreductases | COG0493 |
| DN1_orf00661 | Acetylornithine deacetylase/Succinyl-diaminopimelate desuccinylase and related deacylases | COG0624 |
| DN1_orf00674 | ABC-type amino acid transport/signal transduction systems, periplasmic component/domain | COG0834 |
| DN1_orf00683 | Permeases of the major facilitator superfamily | COG0477 |
| DN1_orf00723 | Glycine cleavage system regulatory protein | COG2716 |
| DN1_orf00739 | Allophanate hydrolase subunit 1 | COG2049 |
| DN1_orf00740 | Allophanate hydrolase subunit 2 | COG1984 |
| DN1_orf00793 | Ornithine/acetylornithine aminotransferase | COG4992 |
| DN1_orf00798 | Glycine/D-amino acid oxidases (deaminating) | COG0665 |
| DN1_orf00804 | Permeases of the drug/metabolite transporter (DMT) superfamily | COG0697 |
| DN1_orf00894 | Spermidine/putrescine-binding periplasmic protein | COG0687 |
| DN1_orf00895 | ABC-type spermidine/putrescine transport systems, ATPase components | COG3842 |
| DN1_orf00896 | Spermidine/putrescine-binding periplasmic protein | COG0687 |
| DN1_orf00897 | ABC-type spermidine/putrescine transport system, permease component I | COG1176 |
| DN1_orf00898 | ABC-type spermidine/putrescine transport system, permease component II | COG1177 |
| DN1_orf00903 | Anthranilate/para-aminobenzoate synthases component I | COG0147 |
| DN1_orf00933 | Anthranilate/para-aminobenzoate synthases component II | COG0512 |
| DN1_orf00934 | Anthranilate phosphoribosyltransferase | COG0547 |
| DN1_orf00935 | Indole-3-glycerol phosphate synthase | COG0134 |
| DN1_orf00938 | S-adenosylmethionine decarboxylase | COG1586 |
| DN1_orf00947 | Acetylglutamate semialdehyde dehydrogenase | COG0002 |
| DN1_orf01012 | Permeases of the major facilitator superfamily | COG0477 |
| DN1_orf01018 | Chorismate mutase | COG1605 |
| DN1_orf01043 | Permeases of the major facilitator superfamily | COG0477 |
| DN1_orf01050 | Anthranilate/para-aminobenzoate synthases component I | COG0147 |
| DN1_orf01052 | 3-deoxy-D-arabino-heptulosonate 7-phosphate (DAHP) synthase | COG3200 |
| DN1_orf01122 | Permeases of the major facilitator superfamily | COG0477 |
| DN1_orf01135 | Amino acid permeases | COG0814 |
| DN1_orf01139 | Glycine/D-amino acid oxidases (deaminating) | COG0665 |
| DN1_orf01158 | Methionine synthase II (cobalamin-independent) | COG0620 |
| DN1_orf01207 | 5-carboxymethyl-2-hydroxymuconate isomerase | COG3232 |
| DN1_orf01214 | Branched-chain amino acid permeases | COG1114 |
| DN1_orf01226 | Permeases of the drug/metabolite transporter (DMT) superfamily | COG0697 |
| DN1_orf01248 | Dipeptidyl aminopeptidases/acylaminoacyl-peptidases | COG1506 |
| DN1_orf01275 | H+/gluconate symporter and related permeases | COG2610 |
| DN1_orf01277 | Permeases of the major facilitator superfamily | COG0477 |
| DN1_orf01286 | Isopropylmalate/homocitrate/citramalate synthases | COG0119 |
| DN1_orf01303 | Lactoylglutathione lyase and related lyases | COG0346 |
| DN1_orf01313 | Transcriptional regulators containing a DNA-binding HTH domain and an aminotransferase domain (MocR family) and their eukaryotic orthologs | COG1167 |
| DN1_orf01319 | Thiamine pyrophosphate-requiring enzymes [acetolactate synthase, pyruvate dehydrogenase (cytochrome), glyoxylate carboligase, phosphonopyruvate decarboxylase] | COG0028 |
| DN1_orf01322 | Predicted branched-chain amino acid permease (azaleucine resistance) | COG1296 |
| DN1_orf01323 | Glutamine synthetase | COG0174 |
| DN1_orf01325 | Amino acid transporters | COG0531 |
| DN1_orf01327 | Na+/serine symporter | COG3633 |
| DN1_orf01332 | Transglutaminase-like enzymes, putative cysteine proteases | COG1305 |
| DN1_orf01354 | ABC-type oligopeptide transport system, periplasmic component | COG4166 |
| DN1_orf01361 | Cysteine sulfinate desulfinase/cysteine desulfurase and related enzymes | COG1104 |
| DN1_orf01371 | Permeases of the major facilitator superfamily | COG0477 |
| DN1_orf01393 | Gamma-aminobutyrate permease and related permeases | COG1113 |
| DN1_orf01395 | Kynureninase | COG3844 |
| DN1_orf01401 | Asparagine synthase (glutamine-hydrolyzing) | COG0367 |
| DN1_orf01424 | Thiamine pyrophosphate-requiring enzymes [acetolactate synthase, pyruvate dehydrogenase (cytochrome), glyoxylate carboligase, phosphonopyruvate decarboxylase] | COG0028 |
| DN1_orf01428 | Allophanate hydrolase subunit 1 | COG2049 |
| DN1_orf01429 | Allophanate hydrolase subunit 2 | COG1984 |
| DN1_orf01435 | Permeases of the major facilitator superfamily | COG0477 |
| DN1_orf01452 | Choline dehydrogenase and related flavoproteins | COG2303 |
| DN1_orf01509 | Threonine dehydrogenase and related Zn-dependent dehydrogenases | COG1063 |
| DN1_orf01555 | Glycine/D-amino acid oxidases (deaminating) | COG0665 |
| DN1_orf01566 | ABC-type amino acid transport system, permease component | COG0765 |
| DN1_orf01568 | ABC-type amino acid transport system, permease component | COG0765 |
| DN1_orf01569 | ABC-type amino acid transport/signal transduction systems, periplasmic component/domain | COG0834 |
| DN1_orf01577 | Permeases of the major facilitator superfamily | COG0477 |
| DN1_orf01582 | Permeases of the major facilitator superfamily | COG0477 |
| DN1_orf01605 | 4-aminobutyrate aminotransferase and related aminotransferases | COG0160 |
| DN1_orf01608 | Amino acid transporters | COG0531 |
| DN1_orf01614 | Glycine/D-amino acid oxidases (deaminating) | COG0665 |
| DN1_orf01662 | Na+/alanine symporter | COG1115 |
| DN1_orf01663 | L-asparaginase/archaeal Glu-tRNAGln amidotransferase subunit D | COG0252 |
| DN1_orf01677 | Permeases of the major facilitator superfamily | COG0477 |
| DN1_orf01681 | Choline dehydrogenase and related flavoproteins | COG2303 |
| DN1_orf01745 | Putative threonine efflux protein | COG1280 |
| DN1_orf01759 | Glycine/D-amino acid oxidases (deaminating) | COG0665 |
| DN1_orf01764 | H+/gluconate symporter and related permeases | COG2610 |
| DN1_orf01871 | Zn-dependent dipeptidase, microsomal dipeptidase homolog | COG2355 |
| DN1_orf01872 | Selenocysteine lyase | COG0520 |
| DN1_orf01902 | 4-aminobutyrate aminotransferase and related aminotransferases | COG0160 |
| DN1_orf01956 | Glycine cleavage system T protein (aminomethyltransferase) | COG0404 |
| DN1_orf01957 | L-serine deaminase | COG1760 |
| DN1_orf01959 | Glycine/serine hydroxymethyltransferase | COG0112 |
| DN1_orf01962 | Glycine cleavage system protein P (pyridoxal-binding), N-terminal domain | COG0403 |
| DN1_orf01964 | Glycine cleavage system H protein (lipoate-binding) | COG0509 |
| DN1_orf01969 | Transcriptional regulator of aromatic amino acids metabolism | COG3283 |
| DN1_orf02004 | Permeases of the major facilitator superfamily | COG0477 |
| DN1_orf02099 | Histidinol-phosphate/aromatic aminotransferase and cobyric acid decarboxylase | COG0079 |
| DN1_orf02102 | Na+/alanine symporter | COG1115 |
| DN1_orf02123 | Lactoylglutathione lyase and related lyases | COG0346 |
| DN1_orf02165 | Permeases of the drug/metabolite transporter (DMT) superfamily | COG0697 |
| DN1_orf02168 | Tryptophan 2,3-dioxygenase (vermilion) | COG3483 |
| DN1_orf02262 | Spermidine/putrescine-binding periplasmic protein | COG0687 |
| DN1_orf02349 | Anthranilate phosphoribosyltransferase | COG0547 |
| DN1_orf02378 | Permeases of the drug/metabolite transporter (DMT) superfamily | COG0697 |
| DN1_orf02458 | Threonine dehydratase | COG1171 |
| DN1_orf02486 | Permeases of the major facilitator superfamily | COG0477 |
| DN1_orf02496 | Cysteine synthase | COG0031 |
| DN1_orf02498 | Putative threonine efflux protein | COG1280 |
| DN1_orf02499 | Spermidine/putrescine-binding periplasmic protein | COG0687 |
| DN1_orf02603 | Glycine/D-amino acid oxidases (deaminating) | COG0665 |
| DN1_orf02617 | Acetylornithine deacetylase/Succinyl-diaminopimelate desuccinylase and related deacylases | COG0624 |
| DN1_orf02652 | Transglutaminase-like enzymes, putative cysteine proteases | COG1305 |
| DN1_orf02679 | Aspartate/tyrosine/aromatic aminotransferase | COG0436 |
| DN1_orf02683 | Predicted carboxypeptidase | COG2866 |
| DN1_orf02689 | Permeases of the major facilitator superfamily | COG0477 |
| DN1_orf02700 | Spermidine synthase | COG0421 |
| DN1_orf02703 | 3-deoxy-D-arabino-heptulosonate 7-phosphate (DAHP) synthase | COG3200 |
| DN1_orf02721 | Lysophospholipase L1 and related esterases | COG2755 |
| DN1_orf02748 | Transglutaminase-like enzymes, putative cysteine proteases | COG1305 |
| DN1_orf02788 | Transcriptional regulators containing a DNA-binding HTH domain and an aminotransferase domain (MocR family) and their eukaryotic orthologs | COG1167 |
| DN1_orf02818 | Putative threonine efflux protein | COG1280 |
| DN1_orf02828 | ABC-type amino acid transport/signal transduction systems, periplasmic component/domain | COG0834 |
| DN1_orf02829 | ABC-type arginine transport system, permease component | COG4215 |
| DN1_orf02830 | ABC-type arginine/histidine transport system, permease component | COG4160 |
| DN1_orf02831 | ABC-type histidine transport system, ATPase component | COG4598 |
| DN1_orf02837 | Putative threonine efflux protein | COG1280 |
| DN1_orf02858 | 3-deoxy-D-arabino-heptulosonate 7-phosphate (DAHP) synthase | COG0722 |
| DN1_orf02879 | Selenocysteine lyase | COG0520 |
| DN1_orf02888 | Branched-chain amino acid aminotransferase/4-amino-4-deoxychorismate lyase | COG0115 |
| DN1_orf02935 | Gamma-aminobutyrate permease and related permeases | COG1113 |
| DN1_orf03006 | Predicted carboxypeptidase | COG2866 |
| DN1_orf03027 | NAD-specific glutamate dehydrogenase | COG2902 |
| DN1_orf03049 | Aminopeptidase N | COG0308 |
| DN1_orf03058 | 1-aminocyclopropane-1-carboxylate deaminase | COG2515 |
| DN1_orf03085 | Cystathionine beta-lyases/cystathionine gamma-synthases | COG0626 |
| DN1_orf03094 | Phosphoribosylanthranilate isomerase | COG0135 |
| DN1_orf03098 | Aspartate-semialdehyde dehydrogenase | COG0136 |
| DN1_orf03100 | Aspartate-semialdehyde dehydrogenase | COG0136 |
| DN1_orf03102 | Isocitrate/isopropylmalate dehydrogenase | COG0473 |
| DN1_orf03104 | 3-isopropylmalate dehydratase small subunit | COG0066 |
| DN1_orf03105 | 3-isopropylmalate dehydratase large subunit | COG0065 |
| DN1_orf03111 | Lysophospholipase L1 and related esterases | COG2755 |
| DN1_orf03113 | Lactoylglutathione lyase and related lyases | COG0346 |
| DN1_orf03127 | Permeases of the major facilitator superfamily | COG0477 |
| DN1_orf03131 | Aspartate/tyrosine/aromatic aminotransferase | COG1448 |
| DN1_orf03140 | 5-enolpyruvylshikimate-3-phosphate synthase | COG0128 |
| DN1_orf03142 | Histidinol-phosphate/aromatic aminotransferase and cobyric acid decarboxylase | COG0079 |
| DN1_orf03143 | Prephenate dehydratase | COG0077 |
| DN1_orf03144 | Phosphoserine aminotransferase | COG1932 |
| DN1_orf03154 | Arginase/agmatinase/formimionoglutamate hydrolase, arginase family | COG0010 |
| DN1_orf03156 | Na+/glutamate symporter | COG0786 |
| DN1_orf03185 | Dihydroxyacid dehydratase/phosphogluconate dehydratase | COG0129 |
| DN1_orf03225 | Permeases of the drug/metabolite transporter (DMT) superfamily | COG0697 |
| DN1_orf03230 | Glycine cleavage system H protein (lipoate-binding) | COG0509 |
| DN1_orf03250 | ABC-type proline/glycine betaine transport systems, periplasmic components | COG2113 |
| DN1_orf03263 | Aspartyl aminopeptidase | COG1362 |
| DN1_orf03270 | ABC-type spermidine/putrescine transport system, permease component II | COG1177 |
| DN1_orf03271 | ABC-type spermidine/putrescine transport systems, ATPase components | COG3842 |
| DN1_orf03272 | Phosphoserine phosphatase | COG0560 |
| DN1_orf03297 | Na+/proline symporter | COG0591 |
| DN1_orf03429 | Permeases of the major facilitator superfamily | COG0477 |
| DN1_orf03432 | Glutamine synthetase | COG0174 |
| DN1_orf03433 | D-serine dehydratase | COG3048 |
| DN1_orf03435 | Permeases of the drug/metabolite transporter (DMT) superfamily | COG0697 |
| DN1_orf03441 | ABC-type branched-chain amino acid transport systems, periplasmic component | COG0683 |
| DN1_orf03476 | Lactoylglutathione lyase and related lyases | COG0346 |
| DN1_orf03524 | Glutamate dehydrogenase/leucine dehydrogenase | COG0334 |
| DN1_orf03534 | Glycine/D-amino acid oxidases (deaminating) | COG0665 |
| DN1_orf03580 | Asparagine synthase (glutamine-hydrolyzing) | COG0367 |
| DN1_orf03592 | Permeases of the major facilitator superfamily | COG0477 |
| DN1_orf03598 | Permeases of the major facilitator superfamily | COG0477 |
| DN1_orf03610 | Permeases of the drug/metabolite transporter (DMT) superfamily | COG0697 |
| DN1_orf03612 | ABC-type amino acid transport/signal transduction systems, periplasmic component/domain | COG0834 |
| DN1_orf03657 | Lactoylglutathione lyase and related lyases | COG0346 |
| DN1_orf03658 | Argininosuccinate synthase | COG0137 |
| DN1_orf03667 | Permeases of the major facilitator superfamily | COG0477 |
| DN1_orf03675 | Ornithine carbamoyltransferase | COG0078 |
| DN1_orf03676 | ABC-type spermidine/putrescine transport systems, ATPase components | COG3842 |
| DN1_orf03738 | Permeases of the major facilitator superfamily | COG0477 |
| DN1_orf03771 | Permeases of the major facilitator superfamily | COG0477 |
| DN1_orf03774 | Amino acid transporters | COG0531 |
| DN1_orf03781 | Glutamate synthase domain 2 | COG0069 |
| DN1_orf03785 | Permeases of the drug/metabolite transporter (DMT) superfamily | COG0697 |
| DN1_orf03787 | ABC-type spermidine/putrescine transport systems, ATPase components | COG3842 |
| DN1_orf03789 | ABC-type spermidine/putrescine transport system, permease component I | COG1176 |
| DN1_orf03790 | ABC-type spermidine/putrescine transport system, permease component II | COG1177 |
| DN1_orf03791 | Spermidine/putrescine-binding periplasmic protein | COG0687 |
| DN1_orf03834 | Na+/alanine symporter | COG1115 |
| DN1_orf03857 | Aspartate/tyrosine/aromatic aminotransferase | COG0436 |
| DN1_orf03867 | Putative threonine efflux protein | COG1280 |
| DN1_orf03869 | Tetrahydrodipicolinate N-succinyltransferase | COG2171 |
| DN1_orf03870 | Selenocysteine lyase | COG0520 |
| DN1_orf03928 | Permeases of the major facilitator superfamily | COG0477 |
| DN1_orf03931 | Choline dehydrogenase and related flavoproteins | COG2303 |
| DN1_orf03949 | Zinc metalloprotease (elastase) | COG3227 |
| DN1_orf03967 | Threonine synthase | COG0498 |
| DN1_orf03970 | Homoserine dehydrogenase | COG0460 |
| DN1_orf03985 | Permeases of the major facilitator superfamily | COG0477 |
| DN1_orf03988 | Lysophospholipase L1 and related esterases | COG2755 |
| DN1_orf04011 | Amino acid permeases | COG0814 |
| DN1_orf04068 | Isopropylmalate/homocitrate/citramalate synthases | COG0119 |
| DN1_orf04076 | Aspartate/tyrosine/aromatic aminotransferase | COG0436 |
| DN1_orf04097 | Cysteine sulfinate desulfinase/cysteine desulfurase and related enzymes | COG1104 |
| DN1_orf04100 | Serine acetyltransferase | COG1045 |
| DN1_orf04121 | Leucyl aminopeptidase | COG0260 |
| DN1_orf04162 | ABC-type amino acid transport/signal transduction systems, periplasmic component/domain | COG0834 |
| DN1_orf04171 | Predicted ornithine cyclodeaminase, mu-crystallin homolog | COG2423 |
| DN1_orf04175 | ABC-type amino acid transport/signal transduction systems, periplasmic component/domain | COG0834 |
| DN1_orf04206 | ABC-type proline/glycine betaine transport systems, permease component | COG1174 |
| DN1_orf04209 | ABC-type proline/glycine betaine transport systems, permease component | COG1174 |
| DN1_orf04212 | ABC-type proline/glycine betaine transport systems, ATPase components | COG1125 |
| DN1_orf04319 | Lactoylglutathione lyase and related lyases | COG0346 |
| DN1_orf04385 | Gamma-glutamyl phosphate reductase | COG0014 |
| DN1_orf04408 | Amino acid transporters | COG0531 |
| DN1_orf04412 | Ethanolamine ammonia-lyase, large subunit | COG4303 |
| DN1_orf04414 | Ethanolamine ammonia-lyase, small subunit | COG4302 |
| DN1_orf04417 | ABC-type amino acid transport/signal transduction systems, periplasmic component/domain | COG0834 |
| DN1_orf04478 | Amino acid transporters | COG0531 |
| DN1_orf04511 | Threonine dehydrogenase and related Zn-dependent dehydrogenases | COG1063 |
| DN1_orf04517 | Choline dehydrogenase and related flavoproteins | COG2303 |
| DN1_orf04548 | 5-carboxymethyl-2-hydroxymuconate isomerase | COG3232 |
| DN1_orf04549 | Permeases of the major facilitator superfamily | COG0477 |
| DN1_orf04558 | Transcriptional regulators containing a DNA-binding HTH domain and an aminotransferase domain (MocR family) and their eukaryotic orthologs | COG1167 |
| DN1_orf04565 | Permeases of the major facilitator superfamily | COG0477 |
| DN1_orf04582 | Putative threonine efflux protein | COG1280 |
| DN1_orf04590 | Threonine dehydrogenase and related Zn-dependent dehydrogenases | COG1063 |
| DN1_orf04606 | Transcriptional regulators containing a DNA-binding HTH domain and an aminotransferase domain (MocR family) and their eukaryotic orthologs | COG1167 |
| DN1_orf04635 | Thiamine pyrophosphate-requiring enzymes [acetolactate synthase, pyruvate dehydrogenase (cytochrome), glyoxylate carboligase, phosphonopyruvate decarboxylase] | COG0028 |
| DN1_orf04640 | Lactoylglutathione lyase and related lyases | COG0346 |
| DN1_orf04644 | Glycine/D-amino acid oxidases (deaminating) | COG0665 |
| DN1_orf04645 | Permeases of the major facilitator superfamily | COG0477 |
| DN1_orf04646 | Dihydrodipicolinate synthase/N-acetylneuraminate lyase | COG0329 |
| DN1_orf04728 | Anthranilate/para-aminobenzoate synthases component I | COG0147 |
| DN1_orf04729 | 3-deoxy-D-arabino-heptulosonate 7-phosphate (DAHP) synthase | COG3200 |
| DN1_orf04764 | Putative threonine efflux protein | COG1280 |
| DN1_orf04820 | Methionine synthase I (cobalamin-dependent), methyltransferase domain | COG0646 |
| DN1_orf04855 | Amino acid transporters | COG0531 |
| DN1_orf04859 | Arginine/lysine/ornithine decarboxylases | COG1982 |
| DN1_orf04868 | ABC-type oligopeptide transport system, periplasmic component | COG4166 |
| DN1_orf04869 | ABC-type oligopeptide transport system, periplasmic component | COG4166 |
| DN1_orf04946 | Anthranilate/para-aminobenzoate synthases component I | COG0147 |
| DN1_orf04947 | Phosphoserine phosphatase | COG0560 |
| DN1_orf04948 | 3'-phosphoadenosine 5'-phosphosulfate sulfotransferase (PAPS reductase)/FAD synthetase and related enzymes | COG0175 |
| DN1_orf04956 | 3-deoxy-D-arabino-heptulosonate 7-phosphate (DAHP) synthase | COG0722 |
| DN1_orf04982 | Transglutaminase-like enzymes, putative cysteine proteases | COG1305 |
| DN1_orf05049 | Spermidine synthase | COG0421 |
| DN1_orf05056 | Permeases of the major facilitator superfamily | COG0477 |
| DN1_orf05057 | Chorismate synthase | COG0082 |
| DN1_orf05097 | Transcriptional regulators containing a DNA-binding HTH domain and an aminotransferase domain (MocR family) and their eukaryotic orthologs | COG1167 |
| DN1_orf05114 | Selenophosphate synthase | COG0709 |
| DN1_orf05118 | Glutaminase | COG2066 |
| DN1_orf05148 | Putative threonine efflux protein | COG1280 |
| DN1_orf05192 | Branched-chain amino acid permeases | COG1114 |
| DN1_orf05225 | Glycine/D-amino acid oxidases (deaminating) | COG0665 |
| DN1_orf05228 | Glutamine synthetase | COG0174 |
| DN1_orf05230 | Glycine/D-amino acid oxidases (deaminating) | COG0665 |
| DN1_orf05329 | Urea transporter | COG4413 |
| DN1_orf05336 | Ethanolamine utilization protein | COG3192 |
| DN1_orf05344 | L-aminopeptidase/D-esterase | COG3191 |
| DN1_orf05346 | Amino acid transporters | COG0531 |
| DN1_orf05444 | Arginase/agmatinase/formimionoglutamate hydrolase, arginase family | COG0010 |
| DN1_orf05449 | Na+/proline symporter | COG0591 |
| DN1_orf05452 | Thiamine pyrophosphate-requiring enzymes [acetolactate synthase, pyruvate dehydrogenase (cytochrome), glyoxylate carboligase, phosphonopyruvate decarboxylase] | COG0028 |
| DN1_orf05459 | Permeases of the major facilitator superfamily | COG0477 |
| DN1_orf05461 | Permeases of the drug/metabolite transporter (DMT) superfamily | COG0697 |
| DN1_orf05464 | Spermidine/putrescine-binding periplasmic protein | COG0687 |
| DN1_orf05508 | Phosphoglycerate dehydrogenase and related dehydrogenases | COG0111 |
| DN1_orf05533 | Permeases of the major facilitator superfamily | COG0477 |
| DN1_orf05540 | Arginine/lysine/ornithine decarboxylases | COG1982 |
| DN1_orf05547 | ABC-type amino acid transport/signal transduction systems, periplasmic component/domain | COG0834 |
| DN1_orf05548 | ABC-type amino acid transport system, permease component | COG0765 |
| DN1_orf05549 | ABC-type amino acid transport system, permease component | COG0765 |
| DN1_orf05550 | ABC-type polar amino acid transport system, ATPase component | COG1126 |
| DN1_orf05551 | Gamma-glutamyltransferase | COG0405 |
| DN1_orf05554 | L-asparaginase/archaeal Glu-tRNAGln amidotransferase subunit D | COG0252 |
| DN1_orf05573 | Threonine dehydratase | COG1171 |
| DN1_orf05591 | Permeases of the major facilitator superfamily | COG0477 |
| DN1_orf05595 | Permeases of the major facilitator superfamily | COG0477 |
| DN1_orf05608 | Protease II | COG1770 |
| DN1_orf05618 | Phosphoglycerate dehydrogenase and related dehydrogenases | COG0111 |
| DN1_orf05632 | Permeases of the major facilitator superfamily | COG0477 |
| DN1_orf05640 | Permeases of the major facilitator superfamily | COG0477 |
| DN1_orf05649 | Histidinol-phosphate/aromatic aminotransferase and cobyric acid decarboxylase | COG0079 |
| DN1_orf05664 | Proline racemase | COG3938 |
| DN1_orf05665 | Glycine/D-amino acid oxidases (deaminating) | COG0665 |
| DN1_orf05670 | Permeases of the drug/metabolite transporter (DMT) superfamily | COG0697 |
|  |  |  |
| DN1_orf05673 | Permeases of the major facilitator superfamily | COG0477 |
| DN1_orf05676 | ABC-type amino acid transport/signal transduction systems, periplasmic component/domain | COG0834 |
| DN1_orf05678 | ABC-type amino acid transport system, permease component | COG0765 |
| DN1_orf05679 | ABC-type amino acid transport system, permease component | COG0765 |
| DN1_orf05680 | ABC-type polar amino acid transport system, ATPase component | COG1126 |
| DN1_orf05681 | Proline racemase | COG3938 |
| DN1_orf05682 | Dihydrodipicolinate synthase/N-acetylneuraminate lyase | COG0329 |
| DN1_orf05717 | Permeases of the major facilitator superfamily | COG0477 |
| DN1_orf05736 | Peptidylarginine deiminase and related enzymes | COG2957 |
| DN1_orf05737 | Spermidine/putrescine-binding periplasmic protein | COG0687 |
| DN1_orf05739 | Peptidylarginine deiminase and related enzymes | COG2957 |
| DN1_orf05751 | Isopropylmalate/homocitrate/citramalate synthases | COG0119 |
| DN1_orf05755 | Asparagine synthase (glutamine-hydrolyzing) | COG0367 |
| DN1_orf05780 | N-Dimethylarginine dimethylaminohydrolase | COG1834 |
| DN1_orf05830 | Acetylornithine deacetylase/Succinyl-diaminopimelate desuccinylase and related deacylases | COG0624 |
| DN1_orf05850 | Amino acid transporters | COG0531 |
| DN1_orf05857 | Phosphoserine phosphatase | COG0560 |
| DN1_orf05875 | Permeases of the major facilitator superfamily | COG0477 |
| DN1_orf05878 | Lactoylglutathione lyase and related lyases | COG0346 |
| DN1_orf05910 | Permeases of the major facilitator superfamily | COG0477 |
| DN1_orf05965 | ABC-type branched-chain amino acid transport systems, periplasmic component | COG0683 |
| DN1_orf05966 | Branched-chain amino acid ABC-type transport system, permease components | COG0559 |
| DN1_orf05967 | ABC-type branched-chain amino acid transport system, permease component | COG4177 |
| DN1_orf05968 | ABC-type branched-chain amino acid transport systems, ATPase component | COG0411 |
| DN1_orf05969 | ABC-type branched-chain amino acid transport systems, ATPase component | COG0410 |
| DN1_orf05979 | Cysteine synthase | COG0031 |
| DN1_orf05981 | Permeases of the drug/metabolite transporter (DMT) superfamily | COG0697 |
| DN1_orf05994 | H+/gluconate symporter and related permeases | COG2610 |
| DN1_orf06025 | Glycine/D-amino acid oxidases (deaminating) | COG0665 |
| DN1_orf06040 | Permeases of the major facilitator superfamily | COG0477 |
| DN1_orf06054 | Dihydrodipicolinate synthase/N-acetylneuraminate lyase | COG0329 |
| DN1_orf06055 | Glycine cleavage system regulatory protein | COG2716 |
| DN1_orf06062 | Anthranilate/para-aminobenzoate synthases component II | COG0512 |
| DN1_orf06063 | Anthranilate/para-aminobenzoate synthases component I | COG0147 |
| DN1_orf06109 | Choline dehydrogenase and related flavoproteins | COG2303 |
| DN1_orf06172 | Cysteine synthase | COG0031 |
| DN1_orf06213 | Aspartokinases | COG0527 |
| DN1_orf06216 | Threonine aldolase | COG2008 |
| DN1_orf06217 | Succinylglutamate desuccinylase | COG2988 |
| DN1_orf06221 | Succinylarginine dihydrolase | COG3724 |
| DN1_orf06223 | Arginine/ornithine N-succinyltransferase beta subunit | COG3138 |
| DN1_orf06225 | Arginine/ornithine N-succinyltransferase beta subunit | COG3138 |
| DN1_orf06226 | Ornithine/acetylornithine aminotransferase | COG4992 |
| DN1_orf06230 | ABC-type histidine transport system, ATPase component | COG4598 |
| DN1_orf06232 | ABC-type arginine/histidine transport system, permease component | COG4160 |
| DN1_orf06233 | ABC-type arginine transport system, permease component | COG4215 |
| DN1_orf06235 | ABC-type amino acid transport/signal transduction systems, periplasmic component/domain | COG0834 |
| DN1_orf06249 | Lactoylglutathione lyase and related lyases | COG0346 |
| DN1_orf06261 | Transcriptional regulator of aromatic amino acids metabolism | COG3283 |
| DN1_orf06262 | Phenylalanine-4-hydroxylase | COG3186 |
| DN1_orf06264 | Aspartate/tyrosine/aromatic aminotransferase | COG1448 |
| DN1_orf06271 | Gamma-aminobutyrate permease and related permeases | COG1113 |
| DN1_orf06272 | 4-hydroxyphenylpyruvate dioxygenase and related hemolysins | COG3185 |
| DN1_orf06294 | Threonine dehydratase | COG1171 |
| DN1_orf06301 | Uncharacterized protein involved in cysteine biosynthesis | COG2981 |
| DN1_orf06347 | Selenocysteine lyase | COG0520 |
| DN1_orf06390 | Gamma-aminobutyrate permease and related permeases | COG1113 |
| DN1_orf06404 | Na+/proline symporter | COG0591 |
| DN1_orf06415 | ABC-type amino acid transport/signal transduction systems, periplasmic component/domain | COG0834 |
| DN1_orf06484 | Lactoylglutathione lyase and related lyases | COG0346 |
| DN1_orf06494 | Permeases of the major facilitator superfamily | COG0477 |
| DN1_orf06650 | Permeases of the major facilitator superfamily | COG0477 |
| DN1_orf06715 | Permeases of the major facilitator superfamily | COG0477 |
| DN1_orf06729 | N-acetylglutamate synthase (N-acetylornithine aminotransferase) | COG1364 |
| DN1_orf06786 | 3'-phosphoadenosine 5'-phosphosulfate sulfotransferase (PAPS reductase)/FAD synthetase and related enzymes | COG0175 |
| DN1_orf06791 | Histidinol-phosphate/aromatic aminotransferase and cobyric acid decarboxylase | COG0079 |
| DN1_orf06792 | Histidinol dehydrogenase | COG0141 |
| DN1_orf06793 | ATP phosphoribosyltransferase | COG0040 |
| DN1_orf06892 | ABC-type dipeptide transport system, periplasmic component | COG0747 |
| DN1_orf06894 | ABC-type dipeptide transport system, periplasmic component | COG0747 |
| DN1_orf06895 | Xaa-Pro aminopeptidase | COG0006 |
| DN1_orf06897 | ABC-type dipeptide transport system, periplasmic component | COG0747 |
| DN1_orf06903 | ABC-type dipeptide transport system, periplasmic component | COG0747 |
| DN1_orf06905 | ABC-type dipeptide/oligopeptide/nickel transport systems, permease components | COG0601 |
| DN1_orf06906 | ABC-type dipeptide/oligopeptide/nickel transport systems, permease components | COG1173 |
| DN1_orf06907 | ABC-type dipeptide/oligopeptide/nickel transport system, ATPase component | COG0444 |
| DN1_orf06909 | ABC-type oligopeptide transport system, ATPase component | COG4608 |
| DN1_orf06914 | Allophanate hydrolase subunit 2 | COG1984 |
| DN1_orf06915 | Allophanate hydrolase subunit 1 | COG2049 |
| DN1_orf06924 | Lactoylglutathione lyase and related lyases | COG0346 |
| DN1_orf06925 | Diaminopimelate decarboxylase | COG0019 |
| DN1_orf07058 | Glycine/D-amino acid oxidases (deaminating) | COG0665 |
| DN1_orf07078 | Glutamate 5-kinase | COG0263 |
| DN1_orf07111 | Glutamate dehydrogenase/leucine dehydrogenase | COG0334 |
| DN1_orf07138 | Glycine/serine hydroxymethyltransferase | COG0112 |
| DN1_orf07170 | Permeases of the major facilitator superfamily | COG0477 |
| DN1_orf07181 | Amino acid transporters | COG0833 |
| DN1_orf07215 | Permeases of the major facilitator superfamily | COG0477 |
| DN1_orf07236 | Phosphoribosylpyrophosphate synthetase | COG0462 |
| DN1_orf07246 | Dipeptidase | COG4690 |
| DN1_orf07269 | Ketol-acid reductoisomerase | COG0059 |
| DN1_orf07270 | Acetolactate synthase, small (regulatory) subunit | COG0440 |
| DN1_orf07273 | Thiamine pyrophosphate-requiring enzymes [acetolactate synthase, pyruvate dehydrogenase (cytochrome), glyoxylate carboligase, phosphonopyruvate decarboxylase] | COG0028 |
| DN1_orf07296 | Aspartate/tyrosine/aromatic aminotransferase | COG0436 |
| DN1_orf07305 | Aspartate/tyrosine/aromatic aminotransferase | COG0436 |
| DN1_orf07312 | Na+/proline symporter | COG0591 |
| DN1_orf07353 | Carbamoylphosphate synthase large subunit (split gene in MJ) | COG0458 |
| DN1_orf07355 | Putative threonine efflux protein | COG1280 |
| DN1_orf07357 | Carbamoylphosphate synthase small subunit | COG0505 |
| DN1_orf07358 | Dihydrodipicolinate reductase | COG0289 |
| DN1_orf07380 | S-adenosylmethionine decarboxylase | COG1586 |
| DN1_orf07381 | Spermidine synthase | COG0421 |
| DN1_orf07388 | Permeases of the drug/metabolite transporter (DMT) superfamily | COG0697 |
| DN1_orf07394 | Permeases of the drug/metabolite transporter (DMT) superfamily | COG0697 |
| DN1_orf07410 | Glycine cleavage system H protein (lipoate-binding) | COG0509 |
| DN1_orf07424 | Amino acid transporters | COG0531 |
| DN1_orf07432 | Selenocysteine synthase [seryl-tRNASer selenium transferase] | COG1921 |
| DN1_orf07474 | Permeases of the drug/metabolite transporter (DMT) superfamily | COG0697 |
| DN1_orf07482 | Arginine decarboxylase (spermidine biosynthesis) | COG1166 |
| DN1_orf07496 | 3-dehydroquinate dehydratase II | COG0757 |
| DN1_orf07514 | ABC-type branched-chain amino acid transport systems, periplasmic component | COG0683 |
| DN1_orf07516 | Branched-chain amino acid ABC-type transport system, permease components | COG0559 |
| DN1_orf07518 | ABC-type branched-chain amino acid transport system, permease component | COG4177 |
| DN1_orf07521 | ABC-type branched-chain amino acid transport systems, ATPase component | COG0410 |
| DN1_orf07524 | Urea amidohydrolase (urease) gamma subunit | COG0831 |
| DN1_orf07526 | Urea amidohydrolase (urease) beta subunit | COG0832 |
| DN1_orf07527 | Urea amidohydrolase (urease) alpha subunit | COG0804 |
| DN1_orf07552 | Permeases of the major facilitator superfamily | COG0477 |
| DN1_orf07572 | Permeases of the major facilitator superfamily | COG0477 |
| DN1_orf07573 | Thiamine pyrophosphate-requiring enzymes [acetolactate synthase, pyruvate dehydrogenase (cytochrome), glyoxylate carboligase, phosphonopyruvate decarboxylase] | COG0028 |
| DN1_orf07577 | Permeases of the major facilitator superfamily | COG0477 |
| DN1_orf07583 | Predicted ornithine cyclodeaminase, mu-crystallin homolog | COG2423 |
| DN1_orf07584 | ABC-type branched-chain amino acid transport systems, ATPase component | COG0410 |
| DN1_orf07585 | ABC-type branched-chain amino acid transport systems, ATPase component | COG0411 |
| DN1_orf07586 | ABC-type branched-chain amino acid transport system, permease component | COG4177 |
| DN1_orf07587 | Branched-chain amino acid ABC-type transport system, permease components | COG0559 |
| DN1_orf07588 | ABC-type branched-chain amino acid transport systems, periplasmic component | COG0683 |
| DN1_orf07606 | Transglutaminase-like enzymes, putative cysteine proteases | COG1305 |
| DN1_orf07628 | ATP phosphoribosyltransferase involved in histidine biosynthesis | COG3705 |
| DN1_orf07661 | Phosphoserine phosphatase | COG0560 |
| DN1_orf07682 | Aspartate/tyrosine/aromatic aminotransferase | COG0436 |
| DN1_orf07686 | Thiamine pyrophosphate-requiring enzymes [acetolactate synthase, pyruvate dehydrogenase (cytochrome), glyoxylate carboligase, phosphonopyruvate decarboxylase] | COG0028 |
| DN1_orf07693 | Amino acid transporters | COG0833 |
| DN1_orf07701 | Spermidine/putrescine-binding periplasmic protein | COG0687 |
| DN1_orf07740 | Branched-chain amino acid aminotransferase/4-amino-4-deoxychorismate lyase | COG0115 |
| DN1_orf07766 | O-acetylhomoserine sulfhydrylase | COG2873 |
| DN1_orf07773 | Permeases of the major facilitator superfamily | COG0477 |
| DN1_orf07779 | NADPH-dependent glutamate synthase beta chain and related oxidoreductases | COG0493 |
| DN1_orf07783 | Glutamate synthase domain 1 | COG0067 |
| DN1_orf07786 | 3-dehydroquinate synthetase | COG0337 |
| DN1_orf07787 | Shikimate kinase | COG0703 |
| DN1_orf07825 | Phosphoribosyl-AMP cyclohydrolase | COG0139 |
| DN1_orf07826 | Phosphoribosyl-ATP pyrophosphohydrolase | COG0140 |
| DN1_orf07835 | ABC-type polar amino acid transport system, ATPase component | COG1126 |
| DN1_orf07836 | ABC-type amino acid transport system, permease component | COG0765 |
| DN1_orf07837 | ABC-type amino acid transport/signal transduction systems, periplasmic component/domain | COG0834 |
| DN1_orf07846 | ABC-type amino acid transport/signal transduction systems, periplasmic component/domain | COG0834 |
| DN1_orf07849 | Glycine/D-amino acid oxidases (deaminating) | COG0665 |
| DN1_orf07856 | N-formylglutamate amidohydrolase | COG3741 |
| DN1_orf07859 | Histidine ammonia-lyase | COG2986 |
| DN1_orf07860 | ABC-type proline/glycine betaine transport system, permease component | COG4176 |
| DN1_orf07861 | ABC-type proline/glycine betaine transport system, ATPase component | COG4175 |
| DN1_orf07862 | ABC-type proline/glycine betaine transport systems, periplasmic components | COG2113 |
| DN1_orf07864 | Gamma-aminobutyrate permease and related permeases | COG1113 |
| DN1_orf07866 | Histidine ammonia-lyase | COG2986 |
| DN1_orf07869 | Urocanate hydratase | COG2987 |
| DN1_orf07874 | ABC-type proline/glycine betaine transport systems, periplasmic components | COG2113 |
| DN1_orf07884 | Lactoylglutathione lyase and related lyases | COG0346 |
| DN1_orf07898 | Glutamine synthetase | COG0174 |
|  |  |  |
| DN1_orf07924 | ABC-type amino acid transport/signal transduction systems, periplasmic component/domain | COG0834 |
| DN1_orf07925 | ABC-type amino acid transport/signal transduction systems, periplasmic component/domain | COG0834 |
| DN1_orf07926 | ABC-type amino acid transport/signal transduction systems, periplasmic component/domain | COG0834 |
| DN1_orf07927 | Imidazoleglycerol-phosphate synthase | COG0107 |
| DN1_orf07928 | Phosphoribosylformimino-5-aminoimidazole carboxamide ribonucleotide (ProFAR) isomerase | COG0106 |
| DN1_orf07931 | Glutamine amidotransferase | COG0118 |
| DN1_orf07932 | Imidazoleglycerol-phosphate dehydratase | COG0131 |
| DN1_orf07946 | ABC-type histidine transport system, ATPase component | COG4598 |
| DN1_orf07947 | ABC-type amino acid transport/signal transduction systems, periplasmic component/domain | COG0834 |
| DN1_orf07949 | ABC-type arginine transport system, permease component | COG4215 |
| DN1_orf07950 | ABC-type arginine/histidine transport system, permease component | COG4160 |
| DN1_orf07958 | Permeases of the major facilitator superfamily | COG0477 |
| DN1_orf07976 | Amino acid transporters | COG0531 |
| DN1_orf07978 | Arginine deiminase | COG2235 |
| DN1_orf07980 | Ornithine carbamoyltransferase | COG0078 |
| DN1_orf07981 | Carbamate kinase | COG0549 |
| DN1_orf07998 | Chorismate mutase | COG1605 |
| DN1_orf08027 | N-acetylglutamate synthase and related acetyltransferases | COG1246 |
| DN1_orf08029 | Acetylornithine deacetylase/Succinyl-diaminopimelate desuccinylase and related deacylases | COG0624 |
| DN1_orf08042 | Glycine cleavage system protein P (pyridoxal-binding), N-terminal domain | COG0403 |
| DN1_orf08043 | Glycine cleavage system H protein (lipoate-binding) | COG0509 |
| DN1_orf08044 | Glycine cleavage system T protein (aminomethyltransferase) | COG0404 |
| DN1_orf08050 | Permeases of the major facilitator superfamily | COG0477 |
| DN1_orf08055 | Xaa-Pro aminopeptidase | COG0006 |
| DN1_orf08091 | Putative threonine efflux protein | COG1280 |
| DN1_orf08113 | Argininosuccinate lyase | COG0165 |
| DN1_orf08131 | Diaminopimelate decarboxylase | COG0019 |
| DN1_orf08133 | Diaminopimelate epimerase | COG0253 |
| DN1_orf08141 | Transcriptional regulators containing a DNA-binding HTH domain and an aminotransferase domain (MocR family) and their eukaryotic orthologs | COG1167 |
| DN1_orf08150 | Nitrogen regulatory protein PII | COG0347 |
| DN1_orf08166 | Thiamine pyrophosphate-requiring enzymes [acetolactate synthase, pyruvate dehydrogenase (cytochrome), glyoxylate carboligase, phosphonopyruvate decarboxylase] | COG0028 |
| DN1_orf08177 | Glycine/D-amino acid oxidases (deaminating) | COG0665 |
| DN1_orf08183 | Glycine/D-amino acid oxidases (deaminating) | COG0665 |
| DN1_orf08186 | Permeases of the major facilitator superfamily | COG0477 |
| DN1_orf08195 | ABC-type dipeptide transport system, periplasmic component | COG0747 |
| DN1_orf08207 | Acetylglutamate kinase | COG0548 |
| DN1_orf08210 | Predicted amino acid aldolase or racemase | COG3616 |
| DN1_orf08270 | Permeases of the major facilitator superfamily | COG0477 |
| DN1_orf08273 | Choline dehydrogenase and related flavoproteins | COG2303 |
| DN1_orf08280 | ABC-type proline/glycine betaine transport system, ATPase component | COG4175 |
| DN1_orf08282 | ABC-type proline/glycine betaine transport system, permease component | COG4176 |
| DN1_orf08284 | ABC-type proline/glycine betaine transport systems, periplasmic components | COG2113 |
| DN1_orf08286 | L-serine deaminase | COG1760 |
| DN1_orf08297 | ABC-type proline/glycine betaine transport systems, periplasmic components | COG2113 |
| DN1_orf08300 | Acetylornithine deacetylase/Succinyl-diaminopimelate desuccinylase and related deacylases | COG0624 |
| DN1_orf08307 | Zn-dependent dipeptidase, microsomal dipeptidase homolog | COG2355 |
| DN1_orf08331 | Threonine aldolase | COG2008 |
| DN1_orf08333 | Glycine/serine hydroxymethyltransferase | COG0112 |
| DN1_orf08335 | Glycine/D-amino acid oxidases (deaminating) | COG0665 |
| DN1_orf08337 | Sarcosine oxidase delta subunit | COG4311 |
| DN1_orf08338 | Glycine cleavage system T protein (aminomethyltransferase) | COG0404 |
| DN1_orf08340 | Sarcosine oxidase gamma subunit | COG4583 |
| DN1_orf08344 | Threonine dehydrogenase and related Zn-dependent dehydrogenases | COG1063 |
| DN1_orf08357 | Aspartate ammonia-lyase | COG1027 |
| DN1_orf08361 | Transcriptional regulators containing a DNA-binding HTH domain and an aminotransferase domain (MocR family) and their eukaryotic orthologs | COG1167 |
| DN1_orf08365 | Amino acid permeases | COG0814 |
| DN1_orf08438 | Permeases of the major facilitator superfamily | COG0477 |
| DN1_orf08479 | Glutamine synthetase | COG0174 |
| DN1_orf08481 | Predicted N-formylglutamate amidohydrolase | COG3931 |
| DN1_orf08482 | Amino acid transporters | COG0531 |
| DN1_orf08499 | Glutamine synthetase | COG0174 |
| DN1_orf08511 | Permeases of the major facilitator superfamily | COG0477 |
| DN1_orf08533 | Phosphoserine phosphatase | COG0560 |
| plasmid_orf00256 | 3'-phosphoadenosine 5'-phosphosulfate sulfotransferase (PAPS reductase)/FAD synthetase and related enzymes | COG0175 |
| plasmid_orf00715 | 3'-phosphoadenosine 5'-phosphosulfate sulfotransferase (PAPS reductase)/FAD synthetase and related enzymes | COG0175 |
