# Supplementary material for: Genome Sequence and Metabolic Analysis of a Fluoranthene-Degrading Strain Pseudomonas aeruginosa DN1
Source: Front Microbiol. 2018 Oct 31;9:2595. doi: 10.3389/fmicb.2018.02595 (PMC6220107; doi:10.3389/fmicb.2018.02595)
Supplement: Supplementary file 3 [file Table_3.DOCX]

**Table S3 | Cell motility**

| **Locus Tag** | **Gene Product Name** | **Function ID** |
| --- | --- | --- |
| DN1 _orf00111 | Flagellar motor protein | COG1360 |
| DN1 _orf00253 | Chemotaxis response regulator containing a CheY-like receiver domain and a methylesterase domain | COG2201 |
| DN1 _orf00254 | Chemotaxis protein; stimulates methylation of MCP proteins | COG1871 |
| DN1 _orf00255 | Methylase of chemotaxis methyl-accepting proteins | COG1352 |
| DN1 _orf00256 | Methyl-accepting chemotaxis protein | COG0840 |
| DN1 _orf00259 | Chemotaxis signal transduction protein | COG0835 |
| DN1 _orf00262 | Chemotaxis protein histidine kinase and related kinases | COG0643 |
| DN1 _orf00265 | Methyl-accepting chemotaxis protein | COG0840 |
| DN1 _orf00585 | Tfp pilus assembly protein, pilus retraction ATPase PilT | COG2805 |
| DN1 _orf00587 | Tfp pilus assembly protein, ATPase PilU | COG5008 |
| DN1 _orf00604 | Chemotaxis signal transduction protein | COG0835 |
| DN1 _orf00605 | Methyl-accepting chemotaxis protein | COG0840 |
| DN1 _orf00608 | Methylase of chemotaxis methyl-accepting proteins | COG1352 |
| DN1 _orf00613 | Chemotaxis response regulator containing a CheY-like receiver domain and a methylesterase domain | COG2201 |
| DN1 _orf01460 | P pilus assembly protein, pilin FimA | COG3539 |
| DN1 _orf01462 | P pilus assembly protein, chaperone PapD | COG3121 |
| DN1 _orf01463 | P pilus assembly protein, porin PapC | COG3188 |
| DN1 _orf01467 | P pilus assembly protein, chaperone PapD | COG3121 |
| DN1 _orf02148 | Methyl-accepting chemotaxis protein | COG0840 |
| DN1 _orf02162 | Methyl-accepting chemotaxis protein | COG0840 |
| DN1 _orf02192 | Bacterial surface proteins containing Ig-like domains | COG5492 |
| DN1 _orf02198 | Bacterial surface proteins containing Ig-like domains | COG5492 |
| DN1 _orf02413 | Methyl-accepting chemotaxis protein | COG0840 |
| DN1 _orf02419 | Methyl-accepting chemotaxis protein | COG0840 |
| DN1 _orf02443 | Type II secretory pathway, pseudopilin PulG | COG2165 |
| DN1 _orf02446 | Type II secretory pathway, pseudopilin PulG | COG2165 |
| DN1 _orf02447 | Type II secretory pathway, pseudopilin PulG | COG2165 |
| DN1 _orf02448 | Type II secretory pathway, component PulF | COG1459 |
| DN1 _orf02450 | Type II secretory pathway, ATPase PulE/Tfp pilus assembly pathway, ATPase PilB | COG2804 |
| DN1 _orf02620 | Methyl-accepting chemotaxis protein | COG0840 |
| DN1 _orf02738 | Methyl-accepting chemotaxis protein | COG0840 |
| DN1 _orf02824 | Methyl-accepting chemotaxis protein | COG0840 |
| DN1 _orf02881 | Tfp pilus assembly protein PilZ | COG3215 |
| DN1 _orf03075 | Type II secretory pathway, pseudopilin PulG | COG2165 |
| DN1 _orf03076 | Type II secretory pathway, pseudopilin PulG | COG2165 |
| DN1 _orf03077 | Type II secretory pathway, component PulF | COG1459 |
| DN1 _orf03080 | Type II secretory pathway, ATPase PulE/Tfp pilus assembly pathway, ATPase PilB | COG2804 |
| DN1 _orf03083 | Type II secretory pathway, component PulD | COG1450 |
| DN1 _orf03097 | Tfp pilus assembly protein FimV | COG3170 |
| DN1 _orf03200 | P pilus assembly/Cpx signaling pathway, periplasmic inhibitor/zinc-resistance associated protein | COG3678 |
| DN1 _orf03409 | Tfp pilus assembly protein FimV | COG3170 |
| DN1 _orf03422 | Methylase of chemotaxis methyl-accepting proteins | COG1352 |
| DN1 _orf03424 | Flagellar basal body P-ring biosynthesis protein | COG1261 |
| DN1 _orf03425 | Negative regulator of flagellin synthesis (anti-sigma28 factor) | COG2747 |
| DN1 _orf03426 | Flagellar biosynthesis/type III secretory pathway chaperone | COG3418 |
| DN1 _orf03873 | ABC-type uncharacterized transport system involved in gliding motility, auxiliary component | COG3225 |
| DN1 _orf03920 | Chemotaxis response regulator containing a CheY-like receiver domain and a methylesterase domain | COG2201 |
| DN1 _orf03923 | Chemotaxis signal transduction protein | COG0835 |
| DN1 _orf03925 | Chemotaxis signal transduction protein | COG0835 |
| DN1 _orf03927 | Methyl-accepting chemotaxis protein | COG0840 |
| DN1 _orf04086 | Tfp pilus assembly protein PilF | COG3063 |
| DN1 _orf04492 | P pilus assembly protein, pilin FimA | COG3539 |
| DN1 _orf04495 | P pilus assembly protein, chaperone PapD | COG3121 |
| DN1 _orf04497 | P pilus assembly protein, porin PapC | COG3188 |
| DN1 _orf04498 | P pilus assembly protein, chaperone PapD | COG3121 |
| DN1 _orf04499 | P pilus assembly protein, pilin FimA | COG3539 |
| DN1 _orf04780 | Type II secretory pathway, component PulD | COG1450 |
| DN1 _orf04995 | Flagellar biosynthesis/type III secretory pathway protein | COG1317 |
| DN1 _orf05007 | Type II secretory pathway, component PulD | COG1450 |
| DN1 _orf05032 | Flagellar biosynthesis/type III secretory pathway ATPase | COG1157 |
| DN1 _orf05036 | Flagellar motor switch/type III secretory pathway protein | COG1886 |
| DN1 _orf05109 | Methyl-accepting chemotaxis protein | COG0840 |
| DN1 _orf05165 | Methyl-accepting chemotaxis protein | COG0840 |
| DN1 _orf05374 | Chemotaxis signal transduction protein | COG0835 |
| DN1 _orf05378 | Flagellar motor protein | COG1360 |
| DN1 _orf05380 | Flagellar motor component | COG1291 |
| DN1 _orf05381 | Chemotaxis response regulator containing a CheY-like receiver domain and a methylesterase domain | COG2201 |
| DN1 _orf05383 | Chemotaxis protein histidine kinase and related kinases | COG0643 |
| DN1 _orf05386 | Chemotaxis protein | COG3143 |
| DN1 _orf05390 | Flagellar GTP-binding protein | COG1419 |
| DN1 _orf05392 | Flagellar biosynthesis pathway, component FlhA | COG1298 |
| DN1 _orf05396 | Flagellar biosynthesis pathway, component FlhB | COG1377 |
| DN1_orf05398 | Flagellar biosynthesis pathway, component FliR | COG1684 |
| DN1 _orf05399 | Flagellar biosynthesis pathway, component FliQ | COG1987 |
| DN1 _orf05401 | Flagellar biosynthesis pathway, component FliP | COG1338 |
| DN1 _orf05404 | Flagellar biogenesis protein | COG3190 |
| DN1 _orf05405 | Flagellar motor switch/type III secretory pathway protein | COG1886 |
| DN1 _orf05408 | Flagellar motor switch protein | COG1868 |
| DN1 _orf05409 | Flagellar basal body-associated protein | COG1580 |
| DN1 _orf05411 | Flagellar hook-length control protein | COG3144 |
| DN1 _orf05688 | Methyl-accepting chemotaxis protein | COG0840 |
| DN1 _orf05913 | Flagellar biosynthesis chaperone | COG2882 |
| DN1 _orf05915 | Flagellar biosynthesis/type III secretory pathway ATPase | COG1157 |
| DN1 _orf05916 | Flagellar biosynthesis/type III secretory pathway protein | COG1317 |
| DN1 _orf05917 | Flagellar motor switch protein | COG1536 |
| DN1 _orf05919 | Flagellar biosynthesis/type III secretory pathway lipoprotein | COG1766 |
| DN1 _orf05921 | Flagellar hook-basal body protein | COG1677 |
| DN1 _orf05928 | Flagellin-specific chaperone FliS | COG1516 |
| DN1 _orf05929 | Flagellin-specific chaperone FliS | COG1516 |
| DN1 _orf05930 | Flagellar capping protein | COG1345 |
| DN1 _orf05931 | Uncharacterized flagellar protein FlaG | COG1334 |
| DN1 _orf05933 | Flagellin and related hook-associated proteins | COG1344 |
| DN1 _orf05945 | Flagellin and related hook-associated proteins | COG1344 |
| DN1 _orf05947 | Flagellar hook-associated protein | COG1256 |
| DN1 _orf05948 | Rod binding protein | COG3951 |
| DN1 _orf05951 | Flagellar basal-body P-ring protein | COG1706 |
| DN1 _orf05952 | Flagellar basal body L-ring protein | COG2063 |
| DN1 _orf05954 | Flagellar basal body rod protein | COG4786 |
| DN1 _orf05955 | Flagellar basal body rod protein | COG4787 |
| DN1 _orf05957 | Flagellar hook protein FlgE | COG1749 |
| DN1 _orf05958 | Flagellar hook capping protein | COG1843 |
| DN1 _orf05960 | Flagellar basal body rod protein | COG1558 |
| DN1 _orf05961 | Flagellar basal body protein | COG1815 |
| DN1 _orf06074 | P pilus assembly protein, porin PapC | COG3188 |
| DN1 _orf06076 | P pilus assembly protein, pilin FimA | COG3539 |
| DN1 _orf06524 | Type II secretory pathway, component PulF | COG1459 |
| DN1 _orf06526 | Type II secretory pathway, ATPase PulE/Tfp pilus assembly pathway, ATPase PilB | COG2804 |
| DN1 _orf06528 | Type II secretory pathway, component PulD | COG1450 |
| DN1 _orf06532 | Type II secretory pathway, pseudopilin PulG | COG2165 |
| DN1 _orf06533 | Type II secretory pathway, pseudopilin PulG | COG2165 |
| DN1 _orf06535 | Type II secretory pathway, pseudopilin PulG | COG2165 |
| DN1 _orf06565 | Methyl-accepting chemotaxis protein | COG0840 |
| DN1 _orf06579 | Flp pilus assembly protein TadC | COG2064 |
| DN1 _orf06590 | Methyl-accepting chemotaxis protein | COG0840 |
| DN1 _orf06592 | Predicted ATPase | COG0433 |
| DN1 _orf06594 | Methyl-accepting chemotaxis protein | COG0840 |
| DN1 _orf06596 | Methyl-accepting chemotaxis protein | COG0840 |
| DN1 _orf06852 | Predicted periplasmic or secreted lipoprotein | COG1724 |
| DN1 _orf06928 | Methyl-accepting chemotaxis protein | COG0840 |
| DN1 _orf06935 | Tfp pilus assembly protein, major pilin PilA | COG4969 |
| DN1 _orf06936 | Type II secretory pathway, ATPase PulE/Tfp pilus assembly pathway, ATPase PilB | COG2804 |
| DN1 _orf06937 | Type II secretory pathway, component PulF | COG1459 |
| DN1 _orf06938 | Type II secretory pathway, prepilin signal peptidase PulO and related peptidases | COG1989 |
| DN1 _orf06995 | Type II secretory pathway, ATPase PulE/Tfp pilus assembly pathway, ATPase PilB | COG2804 |
| DN1 _orf06996 | Type II secretory pathway, component PulF | COG1459 |
| DN1 _orf06999 | Tfp pilus assembly protein, ATPase PilU | COG5008 |
| DN1 _orf07059 | Tfp pilus assembly protein FimT | COG4970 |
| DN1 _orf07060 | Tfp pilus assembly protein PilV | COG4967 |
| DN1 _orf07061 | Tfp pilus assembly protein PilW | COG4966 |
| DN1 _orf07062 | Tfp pilus assembly protein PilX | COG4726 |
| DN1 _orf07064 | Tfp pilus assembly protein, tip-associated adhesin PilY1 | COG3419 |
| DN1 _orf07066 | Tfp pilus assembly protein PilE | COG4968 |
| DN1 _orf07187 | Methyl-accepting chemotaxis protein | COG0840 |
| DN1 _orf07211 | P pilus assembly protein, chaperone PapD | COG3121 |
| DN1 _orf07213 | P pilus assembly protein, porin PapC | COG3188 |
| DN1 _orf07492 | Methyl-accepting chemotaxis protein | COG0840 |
| DN1 _orf07590 | Methyl-accepting chemotaxis protein | COG0840 |
| DN1 _orf07648 | Flagellar motor protein | COG1360 |
| DN1 _orf07649 | Flagellar motor component | COG1291 |
| DN1 _orf07790 | Tfp pilus assembly protein PilP | COG3168 |
| DN1 _orf07791 | Tfp pilus assembly protein PilO | COG3167 |
| DN1 _orf07793 | Tfp pilus assembly protein PilN | COG3166 |
| DN1 _orf07794 | Tfp pilus assembly protein, ATPase PilM | COG4972 |
| DN1 _orf07832 | Methyl-accepting chemotaxis protein | COG0840 |
| DN1 _orf08036 | Type II secretory pathway, ATPase PulE/Tfp pilus assembly pathway, ATPase PilB | COG2804 |
| DN1 _orf08068 | Flagellar basal body-associated protein | COG1580 |
| DN1 _orf08130 | Predicted small periplasmic lipoprotein | COG5567 |
| DN1 _orf08142 | P pilus assembly protein, pilin FimA | COG3539 |
| plasmid_orf00356 | Chemotaxis response regulator containing a CheY-like receiver domain and a methylesterase domain | COG2201 |
| plasmid_orf00359 | Methylase of chemotaxis methyl-accepting proteins | COG1352 |
| plasmid_orf00414 | Type II secretory pathway, ATPase PulE/Tfp pilus assembly pathway, ATPase PilB | COG2804 |
| plasmid_orf00417 | Type II secretory pathway, component PulF | COG1459 |
| plasmid_orf00446 | Tfp pilus assembly protein, pilus retraction ATPase PilT | COG2805 |
