# Supplementary material for: Genome Sequence and Metabolic Analysis of a Fluoranthene-Degrading Strain Pseudomonas aeruginosa DN1
Source: Front Microbiol. 2018 Oct 31;9:2595. doi: 10.3389/fmicb.2018.02595 (PMC6220107; doi:10.3389/fmicb.2018.02595)
Supplement: Supplementary file 4 [file Table_4.DOCX]

**Table S4 | Energy production and conversion**

| **Locus Tag** | **Gene Product Name** | **Function ID** |
| --- | --- | --- |
| DN1_orf00029 | NADPH:quinone reductase and related Zn-dependent oxidoreductases | COG0604 |
| DN1_orf00158 | Heme/copper-type cytochrome/quinol oxidases, subunit 2 | COG1622 |
| DN1_orf00159 | Heme/copper-type cytochrome/quinol oxidases, subunit 1 | COG0843 |
| DN1_orf00163 | Heme/copper-type cytochrome/quinol oxidase, subunit 3 | COG1845 |
| DN1_orf00175 | Na+/H+-dicarboxylate symporters | COG1301 |
| DN1_orf00191 | NAD-dependent aldehyde dehydrogenases | COG1012 |
| DN1_orf00286 | NAD/NADP transhydrogenase alpha subunit | COG3288 |
| DN1_orf00287 | NAD/NADP transhydrogenase alpha subunit | COG3288 |
| DN1_orf00288 | NAD/NADP transhydrogenase beta subunit | COG1282 |
| DN1_orf00314 | NAD-dependent aldehyde dehydrogenases | COG1012 |
| DN1_orf00357 | 2-polyprenyl-6-methoxyphenol hydroxylase and related FAD-dependent oxidoreductases | COG0654 |
| DN1_orf00389 | NAD-dependent aldehyde dehydrogenases | COG1012 |
| DN1_orf00471 | FAD/FMN-containing dehydrogenases | COG0277 |
| DN1_orf00517 | Glycerophosphoryl diester phosphodiesterase | COG0584 |
| DN1_orf00538 | Ferredoxin | COG1145 |
| DN1_orf00542 | NAD-dependent aldehyde dehydrogenases | COG1012 |
| DN1_orf00624 | Cytochrome B561 | COG3038 |
| DN1_orf00664 | Predicted acyl-CoA transferases/carnitine dehydratase | COG1804 |
| DN1_orf00721 | Malate synthase | COG2225 |
| DN1_orf00763 | Cytochrome c, mono- and diheme variants | COG2010 |
| DN1_orf00772 | Cytochrome c, mono- and diheme variants | COG2010 |
| DN1_orf00773 | Cytochrome c551/c552 | COG4654 |
| DN1_orf00775 | Cytochrome c, mono- and diheme variants | COG2010 |
| DN1_orf00778 | Heme/copper-type cytochrome/quinol oxidase, subunit 3 | COG1845 |
| DN1_orf00780 | Cytochrome c, mono- and diheme variants | COG2010 |
| DN1_orf00806 | Cytochrome c556 | COG3909 |
| DN1_orf01045 | 2-polyprenyl-6-methoxyphenol hydroxylase and related FAD-dependent oxidoreductases | COG0654 |
| DN1_orf01163 | Aerobic-type carbon monoxide dehydrogenase, small subunit CoxS/CutS homologs | COG2080 |
| DN1_orf01164 | Aerobic-type carbon monoxide dehydrogenase, middle subunit CoxM/CutM homologs | COG1319 |
| DN1_orf01165 | Aerobic-type carbon monoxide dehydrogenase, large subunit CoxL/CutL homologs | COG1529 |
| DN1_orf01239 | NAD-dependent aldehyde dehydrogenases | COG1012 |
| DN1_orf01250 | Alcohol dehydrogenase, class IV | COG1454 |
| DN1_orf01304 | Pyruvate/2-oxoglutarate dehydrogenase complex, dihydrolipoamide dehydrogenase (E3) component, and related enzymes | COG1249 |
| DN1_orf01410 | Coenzyme F420-dependent N5,N10-methylene tetrahydromethanopterin reductase and related flavin-dependent oxidoreductases | COG2141 |
| DN1_orf01423 | Ferredoxin | COG1145 |
| DN1_orf01455 | NAD-dependent aldehyde dehydrogenases | COG1012 |
| DN1_orf01588 | NAD-dependent aldehyde dehydrogenases | COG1012 |
| DN1_orf01607 | NAD-dependent aldehyde dehydrogenases | COG1012 |
| DN1_orf01609 | Alcohol dehydrogenase, class IV | COG1454 |
| DN1_orf01650 | Succinate dehydrogenase/fumarate reductase, flavoprotein subunit | COG1053 |
| DN1_orf01655 | Pyruvate/2-oxoglutarate dehydrogenase complex, dehydrogenase (E1) component, eukaryotic type, alpha subunit | COG1071 |
| DN1_orf01656 | Pyruvate/2-oxoglutarate dehydrogenase complex, dehydrogenase (E1) component, eukaryotic type, beta subunit | COG0022 |
| DN1_orf01657 | Pyruvate/2-oxoglutarate dehydrogenase complex, dihydrolipoamide acyltransferase (E2) component, and related enzymes | COG0508 |
| DN1_orf01659 | Pyruvate/2-oxoglutarate dehydrogenase complex, dihydrolipoamide dehydrogenase (E3) component, and related enzymes | COG1249 |
| DN1_orf01670 | Cytochrome c553 | COG2863 |
| DN1_orf01678 | Lactate dehydrogenase and related dehydrogenases | COG1052 |
| DN1_orf01683 | Cytochrome c, mono- and diheme variants | COG2010 |
| DN1_orf01724 | FOG: HEAT repeat | COG1413 |
| DN1_orf01726 | Ferredoxin | COG1146 |
| DN1_orf01729 | Succinate dehydrogenase/fumarate reductase, flavoprotein subunit | COG1053 |
| DN1_orf01768 | NAD-dependent aldehyde dehydrogenases | COG1012 |
| DN1_orf01775 | Coenzyme F420-dependent N5,N10-methylene tetrahydromethanopterin reductase and related flavin-dependent oxidoreductases | COG2141 |
| DN1_orf01806 | Coenzyme F420-dependent N5,N10-methylene tetrahydromethanopterin reductase and related flavin-dependent oxidoreductases | COG2141 |
| DN1_orf01813 | Glycerophosphoryl diester phosphodiesterase | COG0584 |
| DN1_orf01818 | Coenzyme F420-dependent N5,N10-methylene tetrahydromethanopterin reductase and related flavin-dependent oxidoreductases | COG2141 |
| DN1_orf01851 | Aerobic-type carbon monoxide dehydrogenase, small subunit CoxS/CutS homologs | COG2080 |
| DN1_orf01852 | Aerobic-type carbon monoxide dehydrogenase, large subunit CoxL/CutL homologs | COG1529 |
| DN1_orf01855 | L-lactate dehydrogenase (FMN-dependent) and related alpha-hydroxy acid dehydrogenases | COG1304 |
| DN1_orf01912 | Protein involved in meta-pathway of phenol degradation | COG4313 |
| DN1_orf02008 | Protein involved in meta-pathway of phenol degradation | COG4313 |
| DN1_orf02013 | Thiol-disulfide isomerase and thioredoxins | COG0526 |
| DN1_orf02014 | Thiol:disulfide interchange protein | COG4232 |
| DN1_orf02021 | Cytochrome c | COG3258 |
| DN1_orf02022 | Cytochrome c553 | COG2863 |
| DN1_orf02023 | CoenzymeF420-dependent N5,N10-methylene tetrahydromethanopterin reductase and related flavin-dependent oxidoreductases | COG2141 |
| DN1_orf02033 | NADPH:quinone reductase and related Zn-dependent oxidoreductases | COG0604 |
| DN1_orf02064 | 2-polyprenylphenol hydroxylase and related flavodoxin oxidoreductases | COG0543 |
| DN1_orf02068 | 2-polyprenylphenol hydroxylase and related flavodoxin oxidoreductases | COG0543 |
| DN1_orf02105 | Predicted oxidoreductases (related to aryl-alcohol dehydrogenases) | COG0667 |
| DN1_orf02255 | 2-polyprenyl-6-methoxyphenol hydroxylase and related FAD-dependent oxidoreductases | COG0654 |
| DN1_orf02270 | Coenzyme F420-dependent N5,N10-methylene tetrahydromethanopterin reductase and related flavin-dependent oxidoreductases | COG2141 |
| DN1_orf02275 | Coenzyme F420-dependent N5,N10-methylene tetrahydromethanopterin reductase and related flavin-dependent oxidoreductases | COG2141 |
| DN1_orf02275 | Coenzyme F420-dependent N5,N10-methylene tetrahydromethanopterin reductase and related flavin-dependent oxidoreductases | COG2141 |
| DN1_orf02369 | Isocitrate dehydrogenases | COG0538 |
| DN1_orf02370 | Monomeric isocitrate dehydrogenase | COG2838 |
| DN1_orf02385 | Isocitrate lyase | COG2224 |
| DN1_orf02390 | NADH:ubiquinone oxidoreductase subunit 3 (chain A) | COG0838 |
| DN1_orf02391 | NADH:ubiquinone oxidoreductase 20 kD subunit and related Fe-S oxidoreductases | COG0377 |
| DN1_orf02393 | NADH:ubiquinone oxidoreductase 49 kD subunit 7 | COG0649 |
| DN1_orf02395 | NADH:ubiquinone oxidoreductase 24 kD subunit | COG1905 |
| DN1_orf02396 | NADH:ubiquinone oxidoreductase, NADH-binding (51 kD) subunit | COG1894 |
| DN1_orf02399 | NADH dehydrogenase/NADH:ubiquinone oxidoreductase 75 kD subunit (chain G) | COG1034 |
| DN1_orf02401 | NADH:ubiquinone oxidoreductase subunit 1 (chain H) | COG1005 |
| DN1_orf02402 | Formate hydrogenlyase subunit 6/NADH:ubiquinone oxidoreductase 23 kD subunit (chain I) | COG1143 |
| DN1_orf02403 | NADH:ubiquinone oxidoreductase subunit 6 (chain J) | COG0839 |
| DN1_orf02404 | NADH:ubiquinone oxidoreductase subunit 11 or 4L (chain K) | COG0713 |
| DN1_orf02405 | NADH:ubiquinone oxidoreductase subunit 5 (chain L)/Multisubunit Na+/H+ antiporter, MnhA subunit | COG1009 |
| DN1_orf02407 | NADH:ubiquinone oxidoreductase subunit 4 (chain M) | COG1008 |
| DN1_orf02408 | NADH:ubiquinone oxidoreductase subunit 2 (chain N) | COG1007 |
| DN1_orf02433 | Flavodoxin reductases (ferredoxin-NADPH reductases) family 1 | COG1018 |
| DN1_orf02453 | NADPH:quinone reductase and related Zn-dependent oxidoreductases | COG0604 |
| DN1_orf02473 | NADH dehydrogenase, FAD-containing subunit | COG1252 |
| DN1_orf02476 | Thiol-disulfide isomerase and thioredoxins | COG0526 |
| DN1_orf02503 | Anaerobic dehydrogenases, typically selenocysteine-containing | COG0243 |
| DN1_orf02505 | Ferredoxin | COG1146 |
| DN1_orf02506 | NADH:flavin oxidoreductases, Old Yellow Enzyme family | COG1902 |
| DN1_orf02840 | NADH:flavin oxidoreductases, Old Yellow Enzyme family | COG1902 |
| DN1_orf02847 | Cytochrome B561 | COG3038 |
| DN1_orf02871 | Electron transfer flavoprotein, alpha subunit | COG2025 |
| DN1_orf02872 | Electron transfer flavoprotein, beta subunit | COG2086 |
| DN1_orf02873 | Dehydrogenases (flavoproteins) | COG0644 |
| DN1_orf02877 | Fe-S oxidoreductase | COG1032 |
| DN1_orf02922 | Glycerophosphoryl diester phosphodiesterase | COG0584 |
| DN1_orf02923 | Pyruvate/2-oxoglutarate dehydrogenase complex, dihydrolipoamide dehydrogenase (E3) component, and related enzymes | COG1249 |
| DN1_orf02927 | Na+-transporting NADH:ubiquinone oxidoreductase, subunit NqrF | COG2871 |
| DN1_orf02928 | Na+-transporting NADH:ubiquinone oxidoreductase, subunit NqrE | COG2209 |
| DN1_orf02929 | Na+-transporting NADH:ubiquinone oxidoreductase, subunit NqrD | COG1347 |
| DN1_orf02930 | Na+-transporting NADH:ubiquinone oxidoreductase, subunit NqrC | COG2869 |
| DN1_orf02931 | Na+-transporting NADH:ubiquinone oxidoreductase, subunit NqrB | COG1805 |
| DN1_orf02933 | Na+-transporting NADH:ubiquinone oxidoreductase, subunit NqrA | COG1726 |
| DN1_orf02971 | Glycerol-3-phosphate dehydrogenase | COG0578 |
| DN1_orf02972 | FAD/FMN-containing dehydrogenases | COG0277 |
| DN1_orf02980 | Thiol-disulfide isomerase and thioredoxins | COG0526 |
| DN1_orf03062 | NADH:flavin oxidoreductases, Old Yellow Enzyme family | COG1902 |
| DN1_orf03102 | Isocitrate/isopropylmalate dehydrogenase | COG0473 |
| DN1_orf03203 | Nitroreductase | COG0778 |
| DN1_orf03273 | NADPH:quinone reductase and related Zn-dependent oxidoreductases | COG0604 |
| DN1_orf03391 | 2-polyprenyl-6-methoxyphenol hydroxylase and related FAD-dependent oxidoreductases | COG0654 |
| DN1_orf03446 | Nitroreductase | COG0778 |
| DN1_orf03481 | Nitrous oxide reductase | COG4263 |
| DN1_orf03488 | Predicted lipoprotein involved in nitrous oxide reduction | COG4314 |
| DN1_orf03489 | Flavodoxin reductases (ferredoxin-NADPH reductases) family 1 | COG1018 |
| DN1_orf03518 | Pyruvate/2-oxoglutarate dehydrogenase complex, dihydrolipoamide acyltransferase (E2) component, and related enzymes | COG0508 |
| DN1_orf03519 | Pyruvate/2-oxoglutarate dehydrogenase complex, dehydrogenase (E1) component, eukaryotic type, beta subunit | COG0022 |
| DN1_orf03521 | Pyruvate/2-oxoglutarate dehydrogenase complex, dehydrogenase (E1) component, eukaryotic type, alpha subunit | COG1071 |
| DN1_orf03544 | Flavodoxins | COG0716 |
| DN1_orf03554 | Coenzyme F420-dependent N5,N10-methylene tetrahydromethanopterin reductase and related flavin-dependent oxidoreductases | COG2141 |
| DN1_orf03606 | Malic enzyme | COG0281 |
| DN1_orf03615 | Glycosyl transferases, related to UDP-glucuronosyltransferase | COG1819 |
| DN1_orf03633 | Predicted NADH:ubiquinone oxidoreductase, subunit RnfA | COG4657 |
| DN1_orf03635 | Predicted NADH:ubiquinone oxidoreductase, subunit RnfB | COG2878 |
| DN1_orf03638 | Predicted NADH:ubiquinone oxidoreductase, subunit RnfC | COG4656 |
| DN1_orf03639 | Predicted NADH:ubiquinone oxidoreductase, subunit RnfD | COG4658 |
| DN1_orf03640 | Predicted NADH:ubiquinone oxidoreductase, subunit RnfG | COG4659 |
| DN1_orf03642 | Predicted NADH:ubiquinone oxidoreductase, subunit RnfE | COG4660 |
| DN1_orf03670 | Anaerobic dehydrogenases, typically selenocysteine-containing | COG0243 |
| DN1_orf03728 | NADPH:quinone reductase and related Zn-dependent oxidoreductases | COG0604 |
| DN1_orf03735 | NAD-dependent aldehyde dehydrogenases | COG1012 |
| DN1_orf03742 | Cytochrome B561 | COG3038 |
| DN1_orf03748 | Glycerol kinase | COG0554 |
| DN1_orf03751 | Glycerol kinase | COG0554 |
| DN1_orf03755 | Glycerol-3-phosphate dehydrogenase | COG0578 |
| DN1_orf03767 | Predicted acyl-CoA transferases/carnitine dehydratase | COG1804 |
| DN1_orf03808 | Ferredoxin | COG1146 |
| DN1_orf03819 | Zn-dependent alcohol dehydrogenases, class III | COG1062 |
| DN1_orf03898 | Phosphoenolpyruvate carboxylase | COG2352 |
| DN1_orf03948 | NADH:flavin oxidoreductases, Old Yellow Enzyme family | COG1902 |
| DN1_orf03963 | Predicted acyl-CoA transferases/carnitine dehydratase | COG1804 |
| DN1_orf04072 | Predicted oxidoreductases (related to aryl-alcohol dehydrogenases) | COG0667 |
| DN1_orf04091 | Ferredoxin | COG0633 |
| DN1_orf04096 | NifU homolog involved in Fe-S cluster formation | COG0822 |
| DN1_orf04142 | NADPH:quinone reductase and related Zn-dependent oxidoreductases | COG0604 |
| DN1_orf04183 | Nitrate reductase gamma subunit | COG2181 |
| DN1_orf04184 | Nitrate reductase delta subunit | COG2180 |
| DN1_orf04186 | Nitrate reductase beta subunit | COG1140 |
| DN1_orf04188 | Nitrate reductase alpha subunit | COG5013 |
| DN1_orf04222 | Lactate dehydrogenase and related dehydrogenases | COG1052 |
| DN1_orf04276 | Cytochrome bd-type quinol oxidase, subunit 2 | COG1294 |
| DN1_orf04277 | Cytochrome bd-type quinol oxidase, subunit 1 | COG1271 |
| DN1_orf04298 | Nitroreductase | COG0778 |
| DN1_orf04316 | Coenzyme F420-dependent N5,N10-methylene tetrahydromethanopterin reductase and related flavin-dependent oxidoreductases | COG2141 |
| DN1_orf04406 | NAD-dependent aldehyde dehydrogenases | COG1012 |
| DN1_orf04423 | Inorganic pyrophosphatase | COG0221 |
| DN1_orf04482 | NAD-dependent aldehyde dehydrogenases | COG1012 |
| DN1_orf04545 | NAD-dependent aldehyde dehydrogenases | COG1012 |
| DN1_orf04556 | Polyferredoxin | COG0348 |
| DN1_orf04572 | FAD/FMN-containing dehydrogenases | COG0277 |
| DN1_orf04587 | Pyruvate/2-oxoglutarate dehydrogenase complex, dehydrogenase (E1) component, eukaryotic type, alpha subunit | COG1071 |
| DN1_orf04588 | Pyruvate/2-oxoglutarate dehydrogenase complex, dehydrogenase (E1) component, eukaryotic type, beta subunit | COG0022 |
| DN1_orf04592 | Coenzyme F420-dependent N5,N10-methylene tetrahydromethanopterin reductase and related flavin-dependent oxidoreductases | COG2141 |
| DN1_orf04649 | NAD-dependent aldehyde dehydrogenases | COG1012 |
| DN1_orf04650 | 2-polyprenyl-6-methoxyphenol hydroxylase and related FAD-dependent oxidoreductases | COG0654 |
| DN1_orf04755 | NADH:ubiquinone oxidoreductase subunit 3 (chain A) | COG0838 |
| DN1_orf04757 | Aerobic-type carbon monoxide dehydrogenase, small subunit CoxS/CutS homologs | COG2080 |
| DN1_orf04759 | Aerobic-type carbon monoxide dehydrogenase, large subunit CoxL/CutL homologs | COG1529 |
| DN1_orf04836 | NADPH:quinone reductase and related Zn-dependent oxidoreductases | COG0604 |
| DN1_orf04903 | Aconitase B | COG1049 |
| DN1_orf04913 | NAD(P)H-nitrite reductase | COG1251 |
| DN1_orf04918 | NAD(P)H-nitrite reductase | COG1251 |
| DN1_orf04955 | Thiol-disulfide isomerase and thioredoxins | COG0526 |
| DN1_orf04970 | Predicted oxidoreductases (related to aryl-alcohol dehydrogenases) | COG0667 |
| DN1_orf04979 | Cytochrome c, mono- and diheme variants | COG2010 |
| DN1_orf05053 | Predicted enolase-phosphatase | COG4229 |
| DN1_orf05156 | Glycerol-3-phosphate dehydrogenase | COG0240 |
| DN1_orf05174 | Aerobic-type carbon monoxide dehydrogenase, small subunit CoxS/CutS homologs | COG2080 |
| DN1_orf05177 | Aerobic-type carbon monoxide dehydrogenase, large subunit CoxL/CutL homologs | COG1529 |
| DN1_orf05178 | Cytochrome c, mono- and diheme variants | COG2010 |
| DN1_orf05193 | Succinyl-CoA synthetase, alpha subunit | COG0074 |
| DN1_orf05194 | Succinyl-CoA synthetase, beta subunit | COG0045 |
| DN1_orf05195 | Pyruvate/2-oxoglutarate dehydrogenase complex, dihydrolipoamide dehydrogenase (E3) component, and related enzymes | COG1249 |
| DN1_orf05198 | Pyruvate/2-oxoglutarate dehydrogenase complex, dihydrolipoamide acyltransferase (E2) component, and related enzymes | COG0508 |
| DN1_orf05200 | 2-oxoglutarate dehydrogenase complex, dehydrogenase (E1) component, and related enzymes | COG0567 |
| DN1_orf05201 | Succinate dehydrogenase/fumarate reductase, Fe-S protein subunit | COG0479 |
| DN1_orf05202 | Succinate dehydrogenase/fumarate reductase, flavoprotein subunit | COG1053 |
| DN1_orf05206 | Citrate synthase | COG0372 |
| DN1_orf05235 | Aconitase A | COG1048 |
| DN1_orf05244 | Cbb3-type cytochrome oxidase, cytochrome c subunit | COG2993 |
| DN1_orf05246 | Cytochrome c, mono- and diheme variants | COG2010 |
| DN1_orf05248 | Cbb3-type cytochrome oxidase, cytochrome c subunit | COG2993 |
| DN1_orf05251 | Cytochrome c, mono- and diheme variants | COG2010 |
| DN1_orf05252 | Polyferredoxin | COG0348 |
| DN1_orf05342 | Glycerol kinase | COG0554 |
| DN1_orf05352 | Thiol-disulfide isomerase and thioredoxins | COG0526 |
| DN1_orf05453 | FAD/FMN-containing dehydrogenases | COG0277 |
| DN1_orf05560 | NADH:flavin oxidoreductases, Old Yellow Enzyme family | COG1902 |
| DN1_orf05583 | Heme/copper-type cytochrome/quinol oxidase, subunit 4 | COG3125 |
| DN1_orf05584 | Heme/copper-type cytochrome/quinol oxidase, subunit 3 | COG1845 |
| DN1_orf05586 | Heme/copper-type cytochrome/quinol oxidases, subunit 1 | COG0843 |
| DN1_orf05587 | Heme/copper-type cytochrome/quinol oxidases, subunit 2 | COG1622 |
| DN1_orf05652 | Nitroreductase | COG0778 |
| DN1_orf05685 | NAD-dependent aldehyde dehydrogenases | COG1012 |
| DN1_orf05687 | Malate/L-lactate dehydrogenases | COG2055 |
| DN1_orf05789 | Predicted acyl-CoA transferases/carnitine dehydratase | COG1804 |
| DN1_orf05792 | Coenzyme F420-dependent N5,N10-methylene tetrahydromethanopterin reductase and related flavin-dependent oxidoreductases | COG2141 |
| DN1_orf05797 | Na+/H+-dicarboxylate symporters | COG1301 |
| DN1_orf05807 | Periplasmic nitrate reductase system, NapE component | COG4459 |
| DN1_orf05809 | Ferredoxin | COG1145 |
| DN1_orf05811 | Anaerobic dehydrogenases, typically selenocysteine-containing | COG0243 |
| DN1_orf05814 | Nitrate reductase cytochrome c-type subunit | COG3043 |
| DN1_orf05815 | Nitrate/TMAO reductases, membrane-bound tetraheme cytochrome c subunit | COG3005 |
| DN1_orf05852 | Alcohol dehydrogenase, class IV | COG1454 |
| DN1_orf05868 | NADPH:quinone reductase and related Zn-dependent oxidoreductases | COG0604 |
| DN1_orf05881 | Predicted oxidoreductases (related to aryl-alcohol dehydrogenases) | COG0667 |
| DN1_orf05987 | Formate hydrogenlyase subunit 3/Multisubunit Na+/H+ antiporter, MnhD subunit | COG0651 |
| DN1_orf05990 | NADH:ubiquinone oxidoreductase subunit 5 (chain L)/Multisubunit Na+/H+ antiporter, MnhA subunit | COG1009 |
| DN1_orf06027 | NAD-dependent aldehyde dehydrogenases | COG1012 |
| DN1_orf06044 | Acyl-CoA synthetase (NDP forming) | COG1042 |
| DN1_orf06106 | NAD-dependent aldehyde dehydrogenases | COG1012 |
| DN1_orf06113 | 2-polyprenyl-6-methoxyphenol hydroxylase and related FAD-dependent oxidoreductases | COG0654 |
| DN1_orf06141 | Acylphosphatases | COG1254 |
| DN1_orf06142 | Thiol-disulfide isomerase and thioredoxins | COG0526 |
| DN1_orf06158 | Thiol-disulfide isomerase and thioredoxins | COG0526 |
| DN1_orf06179 | Lactate dehydrogenase and related dehydrogenases | COG1052 |
| DN1_orf06192 | Cytochrome B561 | COG3038 |
| DN1_orf06222 | NAD-dependent aldehyde dehydrogenases | COG1012 |
| DN1_orf06246 | Predicted acyl-CoA transferases/carnitine dehydratase | COG1804 |
| DN1_orf06275 | NADPH:quinone reductase and related Zn-dependent oxidoreductases | COG0604 |
| DN1_orf06289 | Fumarase | COG0114 |
| DN1_orf06310 | NADH:flavin oxidoreductases, Old Yellow Enzyme family | COG1902 |
| DN1_orf06315 | Acetate kinase | COG0282 |
| DN1_orf06318 | Phosphotransacetylase | COG0280 |
| DN1_orf06378 | Citrate synthase | COG0372 |
| DN1_orf06380 | Aconitase A | COG1048 |
| DN1_orf06408 | Delta 1-pyrroline-5-carboxylate dehydrogenase | COG4230 |
| DN1_orf06457 | NAD-dependent aldehyde dehydrogenases | COG1012 |
| DN1_orf06626 | 2-polyprenylphenol hydroxylase and related flavodoxin oxidoreductases | COG0543 |
| DN1_orf06629 | Tartrate dehydratase alpha subunit/Fumarate hydratase class I, N-terminal domain | COG1951 |
| DN1_orf06657 | Uncharacterized flavoproteins | COG0426 |
| DN1_orf06667 | NADH:flavin oxidoreductases, Old Yellow Enzyme family | COG1902 |
| DN1_orf06688 | Predicted thiol oxidoreductase | COG3488 |
| DN1_orf06766 | Cytochrome c1 | COG2857 |
| DN1_orf06767 | Cytochrome b subunit of the bc complex | COG1290 |
| DN1_orf06769 | Rieske Fe-S protein | COG0723 |
| DN1_orf06772 | Predicted oxidoreductases (related to aryl-alcohol dehydrogenases) | COG0667 |
| DN1_orf06856 | Fumarase | COG0114 |
| DN1_orf06951 | NADH dehydrogenase, FAD-containing subunit | COG1252 |
| DN1_orf07087 | Cytochrome c | COG3258 |
| DN1_orf07158 | Flavodoxin reductases (ferredoxin-NADPH reductases) family 1 | COG1018 |
| DN1_orf07165 | Cytochrome c, mono- and diheme variants | COG2010 |
| DN1_orf07166 | Aerobic-type carbon monoxide dehydrogenase, small subunit CoxS/CutS homologs | COG2080 |
| DN1_orf07168 | Aerobic-type carbon monoxide dehydrogenase, large subunit CoxL/CutL homologs | COG1529 |
| DN1_orf07179 | Lactate dehydrogenase and related dehydrogenases | COG1052 |
| DN1_orf07374 | L-lactate permease | COG1620 |
| DN1_orf07376 | L-lactate dehydrogenase (FMN-dependent) and related alpha-hydroxy acid dehydrogenases | COG1304 |
| DN1_orf07379 | Fe-S oxidoreductase | COG0247 |
| DN1_orf07406 | Glycerophosphoryl diester phosphodiesterase | COG0584 |
| DN1_orf07435 | Cytochrome b subunit of formate dehydrogenase | COG2864 |
| DN1_orf07436 | Fe-S-cluster-containing hydrogenase components 1 | COG0437 |
| DN1_orf07438 | Anaerobic dehydrogenases, typically selenocysteine-containing | COG0243 |
| DN1_orf07439 | Anaerobic dehydrogenases, typically selenocysteine-containing | COG0243 |
| DN1_orf07442 | NADH:flavin oxidoreductases, Old Yellow Enzyme family | COG1902 |
| DN1_orf07468 | Pyruvate/2-oxoglutarate dehydrogenase complex, dihydrolipoamide dehydrogenase (E3) component, and related enzymes | COG1249 |
| DN1_orf07494 | Thiol:disulfide interchange protein | COG4232 |
| DN1_orf07546 | Thiosulfate reductase cytochrome B subunit (membrane anchoring protein) | COG4117 |
| DN1_orf07556 | Flavodoxin reductases (ferredoxin-NADPH reductases) family 1 | COG1018 |
| DN1_orf07570 | NAD-dependent aldehyde dehydrogenases | COG1012 |
| DN1_orf07579 | Flavodoxin reductases (ferredoxin-NADPH reductases) family 1 | COG1018 |
| DN1_orf07601 | Azurin | COG3241 |
| DN1_orf07609 | Fe-S oxidoreductase | COG1032 |
| DN1_orf07644 | Uncharacterized Fe-S protein | COG1600 |
| DN1_orf07688 | Acyl-CoA synthetase (NDP forming) | COG1042 |
| DN1_orf07703 | NADH:flavin oxidoreductases, Old Yellow Enzyme family | COG1902 |
| DN1_orf07709 | Glycerol-3-phosphate dehydrogenase | COG0578 |
| DN1_orf07711 | Predicted oxidoreductases (related to aryl-alcohol dehydrogenases) | COG0667 |
| DN1_orf07744 | Pyruvate dehydrogenase complex, dehydrogenase (E1) component | COG2609 |
| DN1_orf07747 | Pyruvate/2-oxoglutarate dehydrogenase complex, dihydrolipoamide acyltransferase (E2) component, and related enzymes | COG0508 |
| DN1_orf07798 | Malic enzyme | COG0281 |
| DN1_orf07941 | Fe-S cluster protector protein | COG2924 |
| DN1_orf07990 | Uncharacterized protein required for formate dehydrogenase activity | COG1526 |
| DN1_orf07993 | Anaerobic dehydrogenases, typically selenocysteine-containing | COG0243 |
| DN1_orf08000 | Alcohol dehydrogenase, class IV | COG1454 |
| DN1_orf08007 | Nitroreductase | COG0778 |
| DN1_orf08009 | Phosphoenolpyruvate carboxykinase (ATP) | COG1866 |
| DN1_orf08052 | 2-polyprenyl-6-methoxyphenol hydroxylase and related FAD-dependent oxidoreductases | COG0654 |
| DN1_orf08054 | 2-polyprenyl-6-methoxyphenol hydroxylase and related FAD-dependent oxidoreductases | COG0654 |
| DN1_orf08069 | NADPH:quinone reductase and related Zn-dependent oxidoreductases | COG0604 |
| DN1_orf08073 | 2-polyprenylphenol hydroxylase and related flavodoxin oxidoreductases | COG0543 |
| DN1_orf08080 | Thiol-disulfide isomerase and thioredoxins | COG0526 |
| DN1_orf08169 | Acetyl-CoA hydrolase | COG0427 |
| DN1_orf08171 | Cytochrome c5 | COG3245 |
| DN1_orf08187 | NAD-dependent aldehyde dehydrogenases | COG1012 |
| DN1_orf08208 | Protein involved in meta-pathway of phenol degradation | COG4313 |
| DN1_orf08213 | FAD/FMN-containing dehydrogenases | COG0277 |
| DN1_orf08241 | Rubredoxin | COG1773 |
| DN1_orf08243 | Rubredoxin | COG1773 |
| DN1_orf08245 | Fe-S oxidoreductase | COG0247 |
| DN1_orf08248 | FAD/FMN-containing dehydrogenases | COG0277 |
| DN1_orf08249 | FAD/FMN-containing dehydrogenases | COG0277 |
| DN1_orf08274 | NAD-dependent aldehyde dehydrogenases | COG1012 |
| DN1_orf08310 | NADH:flavin oxidoreductases, Old Yellow Enzyme family | COG1902 |
| DN1_orf08313 | Fe-S oxidoreductase | COG0247 |
| DN1_orf08315 | Electron transfer flavoprotein, alpha subunit | COG2025 |
| DN1_orf08317 | Electron transfer flavoprotein, beta subunit | COG2086 |
| DN1_orf08328 | Flavodoxin reductases (ferredoxin-NADPH reductases) family 1 | COG1018 |
| DN1_orf08389 | Acetyl-CoA hydrolase | COG0427 |
| DN1_orf08424 | H+/citrate symporter | COG2851 |
| DN1_orf08445 | Na+/H+-dicarboxylate symporters | COG1301 |
| DN1_orf08455 | Thiol-disulfide isomerase and thioredoxins | COG0526 |
| DN1_orf08457 | Cytochrome c553 | COG2863 |
| DN1_orf08458 | Cytochrome c5 | COG3245 |
| DN1_orf08541 | F0F1-type ATP synthase, epsilon subunit (mitochondrial delta subunit) | COG0355 |
| DN1_orf08544 | F0F1-type ATP synthase, beta subunit | COG0055 |
| DN1_orf08545 | F0F1-type ATP synthase, gamma subunit | COG0224 |
| DN1_orf08546 | F0F1-type ATP synthase, alpha subunit | COG0056 |
| DN1_orf08548 | F0F1-type ATP synthase, delta subunit (mitochondrial oligomycin sensitivity protein) | COG0712 |
| DN1_orf08549 | F0F1-type ATP synthase, subunit b | COG0711 |
| DN1_orf08550 | F0F1-type ATP synthase, subunit c/Archaeal/vacuolar-type H+-ATPase, subunit K | COG0636 |
| DN1_orf08551 | F0F1-type ATP synthase, subunit a | COG0356 |
