# Supplementary material for: Genome Sequence and Metabolic Analysis of a Fluoranthene-Degrading Strain Pseudomonas aeruginosa DN1
Source: Front Microbiol. 2018 Oct 31;9:2595. doi: 10.3389/fmicb.2018.02595 (PMC6220107; doi:10.3389/fmicb.2018.02595)
Supplement: Supplementary file 5 [file Table_5.DOCX]

**Table S5 | General function prediction**

| **Locus Tag** | **Gene Product Name** | **Function ID** |
| --- | --- | --- |
| DN1_orf00018 | FOG: TPR repeat | COG0457 |
| DN1_orf00029 | NADPH:quinone reductase and related Zn-dependent oxidoreductases | COG0604 |
| DN1_orf00080 | Zn-dependent hydrolases, including glyoxylases | COG0491 |
| DN1_orf00089 | Predicted aminopeptidase | COG4324 |
| DN1_orf00092 | Predicted phosphatases | COG0546 |
| DN1_orf00093 | Carbonic anhydrases/acetyltransferases, isoleucine patch superfamily | COG0663 |
| DN1_orf00096 | Predicted nucleic-acid-binding protein containing a Zn-ribbon domain | COG3529 |
| DN1_orf00105 | Serine/threonine protein kinase | COG0515 |
| DN1_orf00127 | Protein of avirulence locus involved in temperature-dependent protein secretion | COG4455 |
| DN1_orf00169 | Uncharacterized protein SCO1/SenC/PrrC, involved in biogenesis of respiratory and photosynthetic systems | COG1999 |
| DN1_orf00170 | Predicted acyltransferase | COG2153 |
| DN1_orf00172 | Dehydrogenases with different specificities (related to short-chain alcohol dehydrogenases) | COG1028 |
| DN1_orf00182 | Plasmid stabilization system protein | COG3668 |
| DN1_orf00197 | Cytosine deaminase and related metal-dependent hydrolases | COG0402 |
| DN1_orf00199 | ABC-type uncharacterized transport systems, ATPase components | COG3845 |
| DN1_orf00201 | ABC-type uncharacterized transport system, permease component | COG4603 |
| DN1_orf00204 | Uncharacterized ABC-type transport system, permease component | COG1079 |
| DN1_orf00209 | Cytosine deaminase and related metal-dependent hydrolases | COG0402 |
| DN1_orf00215 | Uncharacterized ABC-type transport system, periplasmic component/surface lipoprotein | COG1744 |
| DN1_orf00216 | Isopenicillin N synthase and related dioxygenases | COG3491 |
| DN1_orf00268 | Dehydrogenases with different specificities (related to short-chain alcohol dehydrogenases) | COG1028 |
| DN1_orf00298 | Predicted esterase of the alpha/beta hydrolase fold | COG3545 |
| DN1_orf00329 | Permeases of the major facilitator superfamily | COG0477 |
| DN1_orf00333 | Predicted hydrolases or acyltransferases (alpha/beta hydrolase superfamily) | COG0596 |
| DN1_orf00338 | Permeases of the major facilitator superfamily | COG0477 |
| DN1_orf00342 | Predicted dehydrogenases and related proteins | COG0673 |
| DN1_orf00345 | Permeases of the drug/metabolite transporter (DMT) superfamily | COG0697 |
| DN1_orf00348 | Permeases of the major facilitator superfamily | COG0477 |
| DN1_orf00356 | Permeases of the major facilitator superfamily | COG0477 |
| DN1_orf00359 | Histone acetyltransferase HPA2 and related acetyltransferases | COG0454 |
| DN1_orf00360 | FOG: CBS domain | COG0517 |
| DN1_orf00411 | Predicted permeases | COG0730 |
| DN1_orf00427 | Na+/proline symporter | COG0591 |
| DN1_orf00438 | Predicted amidohydrolase | COG0388 |
| DN1_orf00442 | Predicted glutamine amidotransferases | COG2071 |
| DN1_orf00455 | Protein related to penicillin acylase | COG2366 |
| DN1_orf00489 | Predicted aminopeptidases | COG2234 |
| DN1_orf00490 | Acetyltransferase (isoleucine patch superfamily) | COG0110 |
| DN1_orf00499 | Permeases of the major facilitator superfamily | COG0477 |
| DN1_orf00501 | NTP pyrophosphohydrolases including oxidative damage repair enzymes | COG0494 |
| DN1_orf00507 | Predicted permeases | COG0730 |
| DN1_orf00512 | SAM-dependent methyltransferases | COG0500 |
| DN1_orf00522 | Predicted phosphoesterase | COG0622 |
| DN1_orf00529 | Predicted SAM-dependent methyltransferases | COG1092 |
| DN1_orf00530 | Putative intracellular protease/amidase | COG0693 |
| DN1_orf00535 | FOG: TPR repeat, SEL1 subfamily | COG0790 |
| DN1_orf00547 | Predicted hydrolase of the alpha/beta-hydrolase fold | COG0429 |
| DN1_orf00550 | Predicted Zn-dependent peptidases | COG0612 |
| DN1_orf00552 | Predicted Zn-dependent peptidases | COG0612 |
| DN1_orf00564 | Predicted S-adenosylmethionine-dependent methyltransferase | COG0220 |
| DN1_orf00574 | SAM-dependent methyltransferases | COG0500 |
| DN1_orf00579 | Predicted GTPases (dynamin-related) | COG0699 |
| DN1_orf00584 | Predicted enzyme with a TIM-barrel fold | COG0325 |
| DN1_orf00645 | Cytosine deaminase and related metal-dependent hydrolases | COG0402 |
| DN1_orf00652 | NADPH-dependent glutamate synthase beta chain and related oxidoreductases | COG0493 |
| DN1_orf00683 | Permeases of the major facilitator superfamily | COG0477 |
| DN1_orf00715 | Histone acetyltransferase HPA2 and related acetyltransferases | COG0454 |
| DN1_orf00718 | Predicted hydrolases or acyltransferases (alpha/beta hydrolase superfamily) | COG0596 |
| DN1_orf00722 | Histone acetyltransferase HPA2 and related acetyltransferases | COG0454 |
| DN1_orf00724 | Predicted permeases | COG2962 |
| DN1_orf00726 | Putative homoserine kinase type II (protein kinase fold) | COG2334 |
| DN1_orf00727 | N-terminal domain of molybdenum-binding protein | COG2005 |
| DN1_orf00728 | Predicted periplasmic lipoprotein | COG5544 |
| DN1_orf00729 | Predicted amidophosphoribosyltransferases | COG1040 |
| DN1_orf00733 | Uncharacterized proteins, homologs of lactam utilization protein B | COG1540 |
| DN1_orf00746 | Predicted hydrolases or acyltransferases (alpha/beta hydrolase superfamily) | COG0596 |
| DN1_orf00748 | SAM-dependent methyltransferases | COG0500 |
| DN1_orf00765 | Predicted Fe-S oxidoreductases | COG0535 |
| DN1_orf00776 | MoxR-like ATPases | COG0714 |
| DN1_orf00800 | Beta-propeller domains of methanol dehydrogenase type | COG1512 |
| DN1_orf00804 | Permeases of the drug/metabolite transporter (DMT) superfamily | COG0697 |
| DN1_orf00827 | SAM-dependent methyltransferases | COG0500 |
| DN1_orf00828 | Predicted flavoproteins | COG2081 |
| DN1_orf00830 | Predicted metal-dependent hydrolase | COG1451 |
| DN1_orf00832 | Predicted phosphatase/phosphohexomutase | COG0637 |
| DN1_orf00887 | Predicted phosphotransferase related to Ser/Thr protein kinases | COG3178 |
| DN1_orf00902 | Predicted phosphatases | COG0546 |
| DN1_orf00911 | Phage P2 baseplate assembly protein gpV | COG4540 |
| DN1_orf00912 | Phage baseplate assembly protein W | COG3628 |
| DN1_orf00913 | Phage-related baseplate assembly protein | COG3948 |
| DN1_orf00914 | Bacteriophage P2-related tail formation protein | COG4385 |
| DN1_orf00916 | Phage-related tail fibre protein | COG5301 |
| DN1_orf00919 | Phage tail sheath protein FI | COG3497 |
| DN1_orf00920 | Phage tail tube protein FII | COG3498 |
| DN1_orf00924 | Mu-like prophage protein | COG3941 |
| DN1_orf00925 | Phage protein U | COG3499 |
| DN1_orf00927 | P2-like prophage tail protein X | COG5004 |
| DN1_orf00928 | Phage protein D | COG3500 |
| DN1_orf00929 | Predicted chitinase | COG3179 |
| DN1_orf00941 | Diadenosine tetraphosphate (Ap4A) hydrolase and other HIT family hydrolases | COG0537 |
| DN1_orf00943 | Short-chain dehydrogenases of various substrate specificities | COG0300 |
| DN1_orf00945 | Dioxygenases related to 2-nitropropane dioxygenase | COG2070 |
| DN1_orf01012 | Permeases of the major facilitator superfamily | COG0477 |
| DN1_orf01043 | Permeases of the major facilitator superfamily | COG0477 |
| DN1_orf01048 | Predicted epimerase, PhzC/PhzF homolog | COG0384 |
| DN1_orf01055 | SAM-dependent methyltransferases | COG0500 |
| DN1_orf01074 | Mu-like prophage host-nuclease inhibitor protein Gam | COG4396 |
| DN1_orf01093 | Mu-like prophage protein gpG | COG5005 |
| DN1_orf01097 | Mu-like prophage I protein | COG4388 |
| DN1_orf01099 | Mu-like prophage major head subunit gpT | COG4397 |
| DN1_orf01119 | Diadenosine tetraphosphate (Ap4A) hydrolase and other HIT family hydrolases | COG0537 |
| DN1_orf01121 | Predicted dienelactone hydrolase | COG4188 |
| DN1_orf01122 | Permeases of the major facilitator superfamily | COG0477 |
| DN1_orf01138 | Predicted enzyme of the cupin superfamily | COG3450 |
| DN1_orf01146 | SAM-dependent methyltransferases | COG0500 |
| DN1_orf01173 | Acetyltransferases | COG0456 |
| DN1_orf01189 | Predicted double-glycine peptidase | COG3271 |
| DN1_orf01204 | ATPase components of ABC transporters with duplicated ATPase domains | COG0488 |
| DN1_orf01215 | Predicted membrane-associated, metal-dependent hydrolase | COG2194 |
| DN1_orf01226 | Permeases of the drug/metabolite transporter (DMT) superfamily | COG0697 |
| DN1_orf01243 | Metal-dependent hydrolases of the beta-lactamase superfamily I | COG1235 |
| DN1_orf01247 | Predicted Fe-S oxidoreductases | COG0535 |
| DN1_orf01272 | Dehydrogenases with different specificities (related to short-chain alcohol dehydrogenases) | COG1028 |
| DN1_orf01277 | Permeases of the major facilitator superfamily | COG0477 |
| DN1_orf01305 | Predicted Na+-dependent transporter | COG0385 |
| DN1_orf01356 | ABC-type uncharacterized transport system, permease component | COG4174 |
| DN1_orf01358 | ABC-type uncharacterized transport system, permease component | COG4239 |
| DN1_orf01359 | ABC-type uncharacterized transport system, duplicated ATPase component | COG4172 |
| DN1_orf01370 | Predicted phosphatase/phosphohexomutase | COG0637 |
| DN1_orf01371 | Permeases of the major facilitator superfamily | COG0477 |
| DN1_orf01396 | Predicted metal-dependent hydrolase | COG1878 |
| DN1_orf01400 | Phenylpropionate dioxygenase and related ring-hydroxylating dioxygenases, large terminal subunit | COG4638 |
| DN1_orf01404 | Predicted hydrolases or acyltransferases (alpha/beta hydrolase superfamily) | COG0596 |
| DN1_orf01422 | Dehydrogenases with different specificities (related to short-chain alcohol dehydrogenases) | COG1028 |
| DN1_orf01431 | Uncharacterized proteins, homologs of lactam utilization protein B | COG1540 |
| DN1_orf01435 | Permeases of the major facilitator superfamily | COG0477 |
| DN1_orf01457 | Predicted phosphoadenosine phosphosulfate sulfotransferase | COG3969 |
| DN1_orf01481 | Uncharacterized protein (competence- and mitomycin-induced) | COG1546 |
| DN1_orf01482 | Dehydrogenases with different specificities (related to short-chain alcohol dehydrogenases) | COG1028 |
| DN1_orf01490 | General stress protein | COG3729 |
| DN1_orf01507 | Metal-dependent hydrolase | COG3568 |
| DN1_orf01509 | Threonine dehydrogenase and related Zn-dependent dehydrogenases | COG1063 |
| DN1_orf01510 | FOG: CBS domain | COG0517 |
| DN1_orf01525 | Hydrolases of the alpha/beta superfamily | COG1073 |
| DN1_orf01550 | FOG: CBS domain | COG0517 |
| DN1_orf01552 | Uncharacterized NAD(FAD)-dependent dehydrogenases | COG0446 |
| DN1_orf01553 | Uncharacterized NAD(FAD)-dependent dehydrogenases | COG0446 |
| DN1_orf01558 | Putative NADP-dependent oxidoreductases | COG2130 |
| DN1_orf01577 | Permeases of the major facilitator superfamily | COG0477 |
| DN1_orf01579 | Predicted metal-dependent hydrolase of the TIM-barrel fold | COG3618 |
| DN1_orf01582 | Permeases of the major facilitator superfamily | COG0477 |
| DN1_orf01585 | L-alanine-DL-glutamate epimerase and related enzymes of enolase superfamily | COG4948 |
| DN1_orf01592 | Paraquat-inducible protein B | COG3008 |
| DN1_orf01595 | Predicted aminoglycoside phosphotransferase | COG3173 |
| DN1_orf01599 | Dehydrogenases with different specificities (related to short-chain alcohol dehydrogenases) | COG1028 |
| DN1_orf01603 | Predicted symporter | COG4147 |
| DN1_orf01610 | Predicted hydrolases or acyltransferases (alpha/beta hydrolase superfamily) | COG0596 |
| DN1_orf01619 | Predicted exporters of the RND superfamily | COG1033 |
| DN1_orf01620 | Uncharacterized protein related to plant photosystem II stability/assembly factor | COG4447 |
| DN1_orf01624 | Predicted Zn-dependent hydrolases of the beta-lactamase fold | COG2220 |
| DN1_orf01626 | Dehydrogenases with different specificities (related to short-chain alcohol dehydrogenases) | COG1028 |
| DN1_orf01647 | Uncharacterized membrane protein, putative virulence factor | COG0728 |
| DN1_orf01677 | Permeases of the major facilitator superfamily | COG0477 |
| DN1_orf01678 | Lactate dehydrogenase and related dehydrogenases | COG1052 |
| DN1_orf01687 | Uncharacterized enzyme involved in biosynthesis of extracellular polysaccharides | COG2329 |
| DN1_orf01696 | Zn-dependent alcohol dehydrogenases | COG1064 |
| DN1_orf01704 | Predicted flavoprotein | COG0431 |
| DN1_orf01760 | Predicted enzyme of the cupin superfamily | COG3450 |
| DN1_orf01800 | Uncharacterized NAD(FAD)-dependent dehydrogenases | COG0446 |
| DN1_orf01815 | Predicted ATPase | COG1485 |
| DN1_orf01820 | Predicted flavoprotein | COG0431 |
| DN1_orf01861 | Protein related to penicillin acylase | COG2366 |
| DN1_orf01909 | Pirin-related protein | COG1741 |
| DN1_orf01967 | Predicted metal-dependent hydrolase with the TIM-barrel fold | COG1574 |
| DN1_orf01977 | Predicted acetyltransferase | COG3153 |
| DN1_orf02004 | Permeases of the major facilitator superfamily | COG0477 |
| DN1_orf02033 | NADPH:quinone reductase and related Zn-dependent oxidoreductases | COG0604 |
| DN1_orf02043 | FOG: Ankyrin repeat | COG0666 |
| DN1_orf02048 | Serine/threonine protein kinase | COG0515 |
| DN1_orf02056 | L-alanine-DL-glutamate epimerase and related enzymes of enolase superfamily | COG4948 |
| DN1_orf02061 | Phenylpropionate dioxygenase and related ring-hydroxylating dioxygenases, large terminal subunit | COG4638 |
| DN1_orf02066 | Dehydrogenases with different specificities (related to short-chain alcohol dehydrogenases) | COG1028 |
| DN1_orf02071 | Phenylpropionate dioxygenase and related ring-hydroxylating dioxygenases, large terminal subunit | COG4638 |
| DN1_orf02094 | Predicted Zn-dependent proteases and their inactivated homologs | COG0312 |
| DN1_orf02096 | Predicted Zn-dependent proteases and their inactivated homologs | COG0312 |
| DN1_orf02107 | Predicted CDP-diglyceride synthetase/phosphatidate cytidylyltransferase | COG4589 |
| DN1_orf02113 | SAM-dependent methyltransferases | COG0500 |
| DN1_orf02136 | Dehydrogenases with different specificities (related to short-chain alcohol dehydrogenases) | COG1028 |
| DN1_orf02151 | Trans-aconitate methyltransferase | COG4106 |
| DN1_orf02153 | Uncharacterized NAD(FAD)-dependent dehydrogenases | COG0446 |
| DN1_orf02164 | Predicted oxidoreductase related to nitroreductase | COG3560 |
| DN1_orf02165 | Permeases of the drug/metabolite transporter (DMT) superfamily | COG0697 |
| DN1_orf02169 | Putative NADPH-quinone reductase (modulator of drug activity B) | COG2249 |
| DN1_orf02177 | Predicted chitinase | COG3179 |
| DN1_orf02277 | Predicted metal-dependent enzyme of the double-stranded beta helix superfamily | COG5553 |
| DN1_orf02284 | Predicted chitinase | COG3179 |
| DN1_orf02344 | Integral membrane protein, interacts with FtsH | COG0670 |
| DN1_orf02373 | NTP pyrophosphohydrolases including oxidative damage repair enzymes | COG0494 |
| DN1_orf02377 | Uncharacterized protein involved in purine metabolism | COG2915 |
| DN1_orf02378 | Permeases of the drug/metabolite transporter (DMT) superfamily | COG0697 |
| DN1_orf02382 | Histone acetyltransferase HPA2 and related acetyltransferases | COG0454 |
| DN1_orf02387 | Predicted phosphatase | COG3211 |
| DN1_orf02410 | SAM-dependent methyltransferases | COG0500 |
| DN1_orf02411 | Predicted permease | COG0628 |
| DN1_orf02416 | Predicted permease | COG2056 |
| DN1_orf02452 | SAM-dependent methyltransferases | COG0500 |
| DN1_orf02453 | NADPH:quinone reductase and related Zn-dependent oxidoreductases | COG0604 |
| DN1_orf02472 | Predicted hydrolase of the alpha/beta superfamily | COG2819 |
| DN1_orf02483 | Predicted metal-dependent hydrolase with the TIM-barrel fold | COG1574 |
| DN1_orf02486 | Permeases of the major facilitator superfamily | COG0477 |
| DN1_orf02495 | MoxR-like ATPases | COG0714 |
| DN1_orf02507 | Predicted hydrolases or acyltransferases (alpha/beta hydrolase superfamily) | COG0596 |
| DN1_orf02509 | Putative intracellular protease/amidase | COG0693 |
| DN1_orf02525 | Serine/threonine protein kinase | COG0515 |
| DN1_orf02575 | Serine protease inhibitor ecotin | COG4574 |
| DN1_orf02583 | Predicted enzyme of the cupin superfamily | COG3450 |
| DN1_orf02587 | Predicted hydrolases or acyltransferases (alpha/beta hydrolase superfamily) | COG0596 |
| DN1_orf02593 | Predicted epimerase, PhzC/PhzF homolog | COG0384 |
| DN1_orf02596 | Predicted secreted acid phosphatase | COG2503 |
| DN1_orf02637 | Predicted thioesterase | COG0824 |
| DN1_orf02639 | Predicted phosphatase/phosphohexomutase | COG0637 |
| DN1_orf02640 | Predicted HD phosphohydrolase | COG4341 |
| DN1_orf02643 | Enzyme related to GTP cyclohydrolase I | COG0780 |
| DN1_orf02673 | Predicted ATPase (AAA+ superfamily) | COG2607 |
| DN1_orf02685 | SAM-dependent methyltransferases | COG0500 |
| DN1_orf02689 | Permeases of the major facilitator superfamily | COG0477 |
| DN1_orf02710 | Predicted permeases | COG0730 |
| DN1_orf02729 | Predicted acetyltransferases and hydrolases with the alpha/beta hydrolase fold | COG1075 |
| DN1_orf02749 | Uncharacterized conserved protein (some members contain a von Willebrand factor type A (vWA) domain) | COG1721 |
| DN1_orf02750 | MoxR-like ATPases | COG0714 |
| DN1_orf02763 | Predicted metal-dependent membrane protease | COG1266 |
| DN1_orf02767 | Dehydrogenases with different specificities (related to short-chain alcohol dehydrogenases) | COG1028 |
| DN1_orf02778 | Dehydrogenases with different specificities (related to short-chain alcohol dehydrogenases) | COG1028 |
| DN1_orf02816 | Zn-dependent hydrolases, including glyoxylases | COG0491 |
| DN1_orf02821 | Dehydrogenases with different specificities (related to short-chain alcohol dehydrogenases) | COG1028 |
| DN1_orf02826 | Metal-dependent amidase/aminoacylase/carboxypeptidase | COG1473 |
| DN1_orf02844 | Predicted hydrolases or acyltransferases (alpha/beta hydrolase superfamily) | COG0596 |
| DN1_orf02852 | Predicted aminopeptidases | COG2234 |
| DN1_orf02862 | Putative GTPases (G3E family) | COG0523 |
| DN1_orf02868 | Predicted hydrolases or acyltransferases (alpha/beta hydrolase superfamily) | COG0596 |
| DN1_orf02885 | Predicted periplasmic solute-binding protein | COG1559 |
| DN1_orf02893 | Dehydrogenases with different specificities (related to short-chain alcohol dehydrogenases) | COG1028 |
| DN1_orf02897 | Predicted metal-binding, possibly nucleic acid-binding protein | COG1399 |
| DN1_orf02900 | Predicted phosphatases | COG0546 |
| DN1_orf02912 | Predicted hydrolase (metallo-beta-lactamase superfamily) | COG2333 |
| DN1_orf02963 | ATPase components of ABC transporters with duplicated ATPase domains | COG0488 |
| DN1_orf02966 | Uncharacterized Fe-S protein | COG3217 |
| DN1_orf02970 | Sphingosine kinase and enzymes related to eukaryotic diacylglycerol kinase | COG1597 |
| DN1_orf02983 | Predicted flavin-nucleotide-binding protein structurally related to pyridoxine 5'-phosphate oxidase | COG3576 |
| DN1_orf02984 | Zn-dependent hydrolases, including glyoxylases | COG0491 |
|  |  |  |
| DN1_orf03005 | Predicted hydrolases or acyltransferases (alpha/beta hydrolase superfamily) | COG0596 |
| DN1_orf03020 | FOG: TPR repeat | COG0457 |
| DN1_orf03029 | MoxR-like ATPases | COG0714 |
| DN1_orf03031 | Uncharacterized conserved protein (some members contain a von Willebrand factor type A (vWA) domain) | COG1721 |
| DN1_orf03033 | Uncharacterized protein containing a von Willebrand factor type A (vWA) domain | COG2304 |
| DN1_orf03034 | FOG: TPR repeat | COG0457 |
| DN1_orf03042 | Predicted exporters of the RND superfamily | COG1033 |
| DN1_orf03043 | Uncharacterized protein related to plant photosystem II stability/assembly factor | COG4447 |
| DN1_orf03054 | Uncharacterized membrane protein (homolog of Drosophila rhomboid) | COG0705 |
| DN1_orf03063 | Predicted amidohydrolase | COG0388 |
| DN1_orf03084 | Dehydrogenases with different specificities (related to short-chain alcohol dehydrogenases) | COG1028 |
| DN1_orf03087 | Uncharacterized membrane protein, required for colicin V production | COG1286 |
| DN1_orf03103 | SAM-dependent methyltransferases | COG0500 |
| DN1_orf03114 | Dehydrogenases with different specificities (related to short-chain alcohol dehydrogenases) | COG1028 |
| DN1_orf03118 | Predicted thioesterase | COG0824 |
| DN1_orf03127 | Permeases of the major facilitator superfamily | COG0477 |
| DN1_orf03148 | Cytosine deaminase and related metal-dependent hydrolases | COG0402 |
| DN1_orf03151 | Predicted phosphatases | COG0546 |
| DN1_orf03152 | Dehydrogenases with different specificities (related to short-chain alcohol dehydrogenases) | COG1028 |
| DN1_orf03163 | NTP pyrophosphohydrolases including oxidative damage repair enzymes | COG0494 |
| DN1_orf03195 | Predicted metal-dependent phosphoesterases (PHP family) | COG0613 |
| DN1_orf03204 | Predicted Fe-S-cluster oxidoreductase | COG0727 |
| DN1_orf03215 | ABC-type uncharacterized transport system, auxiliary component | COG3218 |
| DN1_orf03224 | EMAP domain | COG0073 |
| DN1_orf03225 | Permeases of the drug/metabolite transporter (DMT) superfamily | COG0697 |
| DN1_orf03227 | Multimeric flavodoxin WrbA | COG0655 |
| DN1_orf03235 | Predicted hydrolases or acyltransferases (alpha/beta hydrolase superfamily) | COG0596 |
| DN1_orf03248 | Predicted symporter | COG4147 |
| DN1_orf03255 | Pirin-related protein | COG1741 |
| DN1_orf03256 | Predicted esterase of the alpha-beta hydrolase superfamily | COG1752 |
| DN1_orf03267 | Uncharacterized proteins of the AP superfamily | COG1524 |
| DN1_orf03269 | ABC-type uncharacterized transport system, permease component | COG4132 |
| DN1_orf03273 | NADPH:quinone reductase and related Zn-dependent oxidoreductases | COG0604 |
| DN1_orf03286 | Predicted Na+-dependent transporter | COG0385 |
| DN1_orf03297 | Na+/proline symporter | COG0591 |
| DN1_orf03303 | Lhr-like helicases | COG1201 |
| DN1_orf03308 | Dehydrogenases with different specificities (related to short-chain alcohol dehydrogenases) | COG1028 |
| DN1_orf03322 | FOG: Ankyrin repeat | COG0666 |
| DN1_orf03333 | Diadenosine tetraphosphate (Ap4A) hydrolase and other HIT family hydrolases | COG0537 |
| DN1_orf03364 | Predicted membrane-associated, metal-dependent hydrolase | COG2194 |
| DN1_orf03384 | Predicted hydrolases or acyltransferases (alpha/beta hydrolase superfamily) | COG0596 |
| DN1_orf03387 | Predicted metal-dependent hydrolase | COG3687 |
| DN1_orf03395 | Dehydrogenases with different specificities (related to short-chain alcohol dehydrogenases) | COG1028 |
| DN1_orf03397 | Ketosteroid isomerase-related protein | COG3631 |
| DN1_orf03407 | Predicted esterase of the alpha-beta hydrolase superfamily | COG1752 |
| DN1_orf03429 | Permeases of the major facilitator superfamily | COG0477 |
| DN1_orf03435 | Permeases of the drug/metabolite transporter (DMT) superfamily | COG0697 |
| DN1_orf03443 | Predicted amidohydrolase | COG0388 |
| DN1_orf03450 | Histone acetyltransferase HPA2 and related acetyltransferases | COG0454 |
| DN1_orf03454 | Metal-dependent hydrolases of the beta-lactamase superfamily I | COG1235 |
| DN1_orf03474 | Dehydrogenases with different specificities (related to short-chain alcohol dehydrogenases) | COG1028 |
| DN1_orf03486 | ABC-type transport system involved in multi-copper enzyme maturation, permease component | COG1277 |
| DN1_orf03504 | ABC-type protease/lipase transport system, ATPase and permease components | COG4618 |
| DN1_orf03535 | Predicted enzyme of the cupin superfamily | COG3450 |
| DN1_orf03537 | Dehydrogenases with different specificities (related to short-chain alcohol dehydrogenases) | COG1028 |
| DN1_orf03539 | Predicted hydrolases or acyltransferases (alpha/beta hydrolase superfamily) | COG0596 |
| DN1_orf03542 | Putative effector of murein hydrolase LrgA | COG1380 |
| DN1_orf03546 | Dehydrogenases with different specificities (related to short-chain alcohol dehydrogenases) | COG1028 |
| DN1_orf03556 | Predicted flavoprotein | COG0431 |
| DN1_orf03563 | Predicted dehydrogenase | COG0579 |
| DN1_orf03578 | Predicted permeases | COG0730 |
| DN1_orf03584 | Acetyltransferases | COG0456 |
| DN1_orf03592 | Permeases of the major facilitator superfamily | COG0477 |
| DN1_orf03598 | Permeases of the major facilitator superfamily | COG0477 |
| DN1_orf03605 | NTP pyrophosphohydrolases including oxidative damage repair enzymes | COG0494 |
| DN1_orf03610 | Permeases of the drug/metabolite transporter (DMT) superfamily | COG0697 |
| DN1_orf03611 | Predicted permeases | COG2962 |
| DN1_orf03616 | Predicted hydrolases or acyltransferases (alpha/beta hydrolase superfamily) | COG0596 |
| DN1_orf03646 | SAM-dependent methyltransferases | COG0500 |
| DN1_orf03667 | Permeases of the major facilitator superfamily | COG0477 |
| DN1_orf03685 | FOG: TPR repeat, SEL1 subfamily | COG0790 |
| DN1_orf03728 | NADPH:quinone reductase and related Zn-dependent oxidoreductases | COG0604 |
| DN1_orf03738 | Permeases of the major facilitator superfamily | COG0477 |
| DN1_orf03745 | Predicted epimerase, PhzC/PhzF homolog | COG0384 |
| DN1_orf03758 | Uncharacterized membrane protein required for alginate biosynthesis | COG3136 |
| DN1_orf03759 | Predicted hydrolases or acyltransferases (alpha/beta hydrolase superfamily) | COG0596 |
| DN1_orf03771 | Permeases of the major facilitator superfamily | COG0477 |
| DN1_orf03776 | Predicted amidohydrolase | COG0388 |
| DN1_orf03785 | Permeases of the drug/metabolite transporter (DMT) superfamily | COG0697 |
| DN1_orf03798 | Predicted Rossmann fold nucleotide-binding protein | COG1611 |
| DN1_orf03800 | Uncharacterized protein conserved in bacteria | COG2137 |
| DN1_orf03804 | Uncharacterized protein (competence- and mitomycin-induced) | COG1546 |
| DN1_orf03812 | Predicted acid phosphatase | COG0496 |
| DN1_orf03818 | Predicted esterase | COG0627 |
| DN1_orf03821 | Predicted transporter component | COG2391 |
| DN1_orf03864 | Predicted Fe-S-cluster oxidoreductase | COG0727 |
| DN1_orf03871 | SufE protein probably involved in Fe-S center assembly | COG2166 |
| DN1_orf03874 | ABC-type transport system involved in multi-copper enzyme maturation, permease component | COG1277 |
| DN1_orf03890 | SAM-dependent methyltransferases | COG0500 |
| DN1_orf03906 | Predicted phosphatase homologous to the C-terminal domain of histone macroH2A1 | COG2110 |
| DN1_orf03908 | Hydrolases of the alpha/beta superfamily | COG1073 |
| DN1_orf03924 | FOG: TPR repeat | COG0457 |
| DN1_orf03928 | Permeases of the major facilitator superfamily | COG0477 |
| DN1_orf03982 | ABC-type uncharacterized transport system, permease component | COG4137 |
| DN1_orf03985 | Permeases of the major facilitator superfamily | COG0477 |
| DN1_orf03990 | Predicted Fe-S protein | COG3313 |
| DN1_orf03991 | Carbonic anhydrases/acetyltransferases, isoleucine patch superfamily | COG0663 |
| DN1_orf03992 | NTP pyrophosphohydrolases including oxidative damage repair enzymes | COG0494 |
| DN1_orf04020 | Predicted ATPase | COG0433 |
| DN1_orf04037 | FOG: CBS domain | COG0517 |
| DN1_orf04044 | Predicted permeases | COG0730 |
| DN1_orf04075 | Predicted amidohydrolase | COG0388 |
| DN1_orf04077 | Predicted GTPases | COG1160 |
| DN1_orf04088 | Predicted Fe-S-cluster redox enzyme | COG0820 |
| DN1_orf04117 | Predicted permeases | COG0795 |
| DN1_orf04118 | Predicted permeases | COG0795 |
| DN1_orf04120 | Hydrolases of the alpha/beta superfamily | COG1073 |
| DN1_orf04132 | ABC-type uncharacterized transport system, periplasmic component | COG2984 |
| DN1_orf04133 | ABC-type uncharacterized transport system, permease component | COG4120 |
| DN1_orf04134 | ABC-type uncharacterized transport system, ATPase component | COG1101 |
| DN1_orf04138 | Predicted SAM-dependent methyltransferase | COG3129 |
| DN1_orf04142 | NADPH:quinone reductase and related Zn-dependent oxidoreductases | COG0604 |
| DN1_orf04148 | TRAP-type uncharacterized transport system, periplasmic component | COG2358 |
| DN1_orf04150 | Nucleoid-associated protein | COG3081 |
| DN1_orf04156 | Acetyltransferase (isoleucine patch superfamily) | COG0110 |
| DN1_orf04164 | Predicted esterase | COG0400 |
| DN1_orf04172 | Predicted dehydrogenase | COG0579 |
| DN1_orf04198 | SAM-dependent methyltransferases | COG0500 |
| DN1_orf04199 | Dehydrogenases with different specificities (related to short-chain alcohol dehydrogenases) | COG1028 |
| DN1_orf04222 | Lactate dehydrogenase and related dehydrogenases | COG1052 |
| DN1_orf04240 | Predicted extracellular nuclease | COG2374 |
| DN1_orf04295 | Predicted phosphatases | COG0546 |
| DN1_orf04309 | Predicted flavoproteins | COG2081 |
| DN1_orf04320 | Dehydrogenases with different specificities (related to short-chain alcohol dehydrogenases) | COG1028 |
| DN1_orf04334 | Predicted Zn-dependent hydrolases of the beta-lactamase fold | COG2220 |
| DN1_orf04350 | FOG: TPR repeat, SEL1 subfamily | COG0790 |
| DN1_orf04355 | Predicted metal-dependent hydrolase | COG0319 |
| DN1_orf04370 | Predicted hydrolases or acyltransferases (alpha/beta hydrolase superfamily) | COG0596 |
| DN1_orf04393 | Uncharacterized protein, similar to the N-terminal domain of Lon protease | COG2802 |
| DN1_orf04395 | Putative effector of murein hydrolase LrgA | COG1380 |
| DN1_orf04400 | Predicted periplasmic lipoprotein | COG5645 |
| DN1_orf04415 | Histone acetyltransferase HPA2 and related acetyltransferases | COG0454 |
| DN1_orf04436 | ABC-type transport system involved in multi-copper enzyme maturation, permease component | COG1277 |
| DN1_orf04443 | L-alanine-DL-glutamate epimerase and related enzymes of enolase superfamily | COG4948 |
| DN1_orf04449 | Predicted aspartyl protease | COG3577 |
| DN1_orf04462 | Predicted methyltransferase | COG3897 |
| DN1_orf04484 | SAM-dependent methyltransferases | COG0500 |
| DN1_orf04489 | Dehydrogenases with different specificities (related to short-chain alcohol dehydrogenases) | COG1028 |
| DN1_orf04503 | Dehydrogenases with different specificities (related to short-chain alcohol dehydrogenases) | COG1028 |
| DN1_orf04506 | Conserved protein/domain typically associated with flavoprotein oxygenases, DIM6/NTAB family | COG1853 |
| DN1_orf04511 | Threonine dehydrogenase and related Zn-dependent dehydrogenases | COG1063 |
| DN1_orf04513 | Dehydrogenases with different specificities (related to short-chain alcohol dehydrogenases) | COG1028 |
| DN1_orf04519 | Dehydrogenases with different specificities (related to short-chain alcohol dehydrogenases) | COG1028 |
| DN1_orf04533 | Histone acetyltransferase HPA2 and related acetyltransferases | COG0454 |
| DN1_orf04534 | Predicted Rossmann fold nucleotide-binding protein | COG1611 |
| DN1_orf04549 | Permeases of the major facilitator superfamily | COG0477 |
| DN1_orf04565 | Permeases of the major facilitator superfamily | COG0477 |
| DN1_orf04585 | Dehydrogenases with different specificities (related to short-chain alcohol dehydrogenases) | COG1028 |
| DN1_orf04589 | Predicted hydrolases or acyltransferases (alpha/beta hydrolase superfamily) | COG0596 |
| DN1_orf04590 | Threonine dehydrogenase and related Zn-dependent dehydrogenases | COG1063 |
| DN1_orf04608 | Histone acetyltransferase HPA2 and related acetyltransferases | COG0454 |
| DN1_orf04609 | Aldo/keto reductases, related to diketogulonate reductase | COG0656 |
| DN1_orf04614 | Putative intracellular protease/amidase | COG0693 |
| DN1_orf04629 | SAM-dependent methyltransferases | COG0500 |
| DN1_orf04645 | Permeases of the major facilitator superfamily | COG0477 |
| DN1_orf04661 | Zn-dependent hydrolases, including glyoxylases | COG0491 |
| DN1_orf04664 | Dioxygenases related to 2-nitropropane dioxygenase | COG2070 |
| DN1_orf04687 | Mu-like prophage major head subunit gpT | COG4397 |
| DN1_orf04690 | Mu-like prophage I protein | COG4388 |
| DN1_orf04694 | Mu-like prophage protein gpG | COG5005 |
| DN1_orf04713 | Mu-like prophage host-nuclease inhibitor protein Gam | COG4396 |
| DN1_orf04725 | Predicted epimerase, PhzC/PhzF homolog | COG0384 |
| DN1_orf04741 | Protein related to penicillin acylase | COG2366 |
| DN1_orf04753 | Histone acetyltransferase HPA2 and related acetyltransferases | COG0454 |
| DN1_orf04763 | Predicted hydrolases of HD superfamily | COG1896 |
| DN1_orf04827 | Predicted Fe-S-cluster redox enzyme | COG0820 |
| DN1_orf04835 | Predicted alpha/beta hydrolase | COG4757 |
| DN1_orf04836 | NADPH:quinone reductase and related Zn-dependent oxidoreductases | COG0604 |
| DN1_orf04840 | Predicted aminoglycoside phosphotransferase | COG3173 |
| DN1_orf04841 | Dehydrogenases with different specificities (related to short-chain alcohol dehydrogenases) | COG1028 |
| DN1_orf04843 | Dehydrogenases with different specificities (related to short-chain alcohol dehydrogenases) | COG1028 |
| DN1_orf04847 | Predicted permeases | COG0730 |
| DN1_orf04860 | Ketosteroid isomerase-related protein | COG3631 |
| DN1_orf04864 | SAM-dependent methyltransferases | COG0500 |
| DN1_orf04865 | Zn-dependent hydrolases, including glyoxylases | COG0491 |
| DN1_orf04871 | ABC-type uncharacterized transport system, permease component | COG4174 |
| DN1_orf04873 | ABC-type uncharacterized transport system, permease component | COG4239 |
| DN1_orf04874 | ABC-type uncharacterized transport system, duplicated ATPase component | COG4172 |
| DN1_orf04902 | Predicted Fe-S protein | COG3313 |
| DN1_orf04915 | Ferredoxin subunits of nitrite reductase and ring-hydroxylating dioxygenases | COG2146 |
| DN1_orf04940 | Predicted double-glycine peptidase | COG3271 |
| DN1_orf04958 | Predicted acetyltransferase | COG2388 |
| DN1_orf04962 | Predicted phosphatase homologous to the C-terminal domain of histone macroH2A1 | COG2110 |
| DN1_orf04978 | Predicted permeases | COG0679 |
| DN1_orf05017 | FOG: TPR repeat | COG0457 |
| DN1_orf05056 | Permeases of the major facilitator superfamily | COG0477 |
| DN1_orf05058 | Hydrolases of the alpha/beta superfamily | COG1073 |
| DN1_orf05069 | Predicted enzyme related to lactoylglutathione lyase | COG3324 |
| DN1_orf05070 | Serine/threonine protein kinase | COG0515 |
| DN1_orf05100 | Predicted Na+-dependent transporter | COG0385 |
| DN1_orf05102 | Dehydrogenases with different specificities (related to short-chain alcohol dehydrogenases) | COG1028 |
| DN1_orf05103 | Putative NADP-dependent oxidoreductases | COG2130 |
| DN1_orf05110 | Conserved protein/domain typically associated with flavoprotein oxygenases, DIM6/NTAB family | COG1853 |
| DN1_orf05113 | Predicted ATPase | COG2603 |
| DN1_orf05116 | Predicted esterase of the alpha-beta hydrolase superfamily | COG1752 |
| DN1_orf05141 | Predicted permease | COG0628 |
| DN1_orf05145 | Predicted hydrolases or acyltransferases (alpha/beta hydrolase superfamily) | COG0596 |
| DN1_orf05146 | Predicted hydrolases or acyltransferases (alpha/beta hydrolase superfamily) | COG0596 |
| DN1_orf05159 | ABC-type uncharacterized transport system, periplasmic component | COG2984 |
| DN1_orf05208 | Predicted Fe-S-cluster oxidoreductase | COG0727 |
| DN1_orf05212 | Inhibitor of the KinA pathway to sporulation, predicted exonuclease | COG5018 |
| DN1_orf05234 | Predicted SAM-dependent methyltransferase | COG2933 |
| DN1_orf05241 | Predicted hydrolase of the alpha/beta-hydrolase fold | COG3571 |
| DN1_orf05263 | Predicted metal-dependent hydrolase | COG3687 |
| DN1_orf05269 | Dehydrogenases with different specificities (related to short-chain alcohol dehydrogenases) | COG1028 |
| DN1_orf05293 | Cytosine deaminase and related metal-dependent hydrolases | COG0402 |
| DN1_orf05297 | Permeases | COG2252 |
| DN1_orf05300 | Transthyretin-like protein | COG2351 |
| DN1_orf05315 | Predicted protein tyrosine phosphatase | COG4551 |
| DN1_orf05318 | Uncharacterized protein, possibly involved in utilization of glycolate and propanediol | COG3193 |
| DN1_orf05319 | Glyoxylate carboligase | COG3960 |
| DN1_orf05339 | Predicted dehydrogenase | COG0579 |
| DN1_orf05341 | Uncharacterized NAD(FAD)-dependent dehydrogenases | COG0446 |
| DN1_orf05368 | Dehydrogenases with different specificities (related to short-chain alcohol dehydrogenases) | COG1028 |
| DN1_orf05394 | Beta-propeller domains of methanol dehydrogenase type | COG1512 |
| DN1_orf05395 | Beta-propeller domains of methanol dehydrogenase type | COG1512 |
| DN1_orf05412 | Predicted metal-binding protein related to the C-terminal domain of SecA | COG3318 |
| DN1_orf05430 | Histone acetyltransferase HPA2 and related acetyltransferases | COG0454 |
| DN1_orf05433 | ATPase components of ABC transporters with duplicated ATPase domains | COG0488 |
| DN1_orf05449 | Na+/proline symporter | COG0591 |
| DN1_orf05455 | Zn-dependent hydrolases, including glyoxylases | COG0491 |
| DN1_orf05459 | Permeases of the major facilitator superfamily | COG0477 |
| DN1_orf05461 | Permeases of the drug/metabolite transporter (DMT) superfamily | COG0697 |
| DN1_orf05469 | Predicted SAM-dependent methyltransferases | COG1092 |
| DN1_orf05471 | Predicted nucleotidyltransferases | COG1708 |
| DN1_orf05476 | Predicted O-methyltransferase | COG4122 |
| DN1_orf05477 | Predicted ester cyclase | COG5485 |
| DN1_orf05498 | Dehydrogenases with different specificities (related to short-chain alcohol dehydrogenases) | COG1028 |
| DN1_orf05504 | Histone acetyltransferase HPA2 and related acetyltransferases | COG0454 |
| DN1_orf05513 | Predicted epimerase, PhzC/PhzF homolog | COG0384 |
| DN1_orf05523 | Predicted permease, DMT superfamily | COG5006 |
| DN1_orf05533 | Permeases of the major facilitator superfamily | COG0477 |
| DN1_orf05545 | Dehydrogenases with different specificities (related to short-chain alcohol dehydrogenases) | COG1028 |
| DN1_orf05562 | SAM-dependent methyltransferases | COG0500 |
| DN1_orf05563 | Hemolysins and related proteins containing CBS domains | COG1253 |
| DN1_orf05567 | Dehydrogenases with different specificities (related to short-chain alcohol dehydrogenases) | COG1028 |
| DN1_orf05568 | Uncharacterized protein, possibly involved in utilization of glycolate and propanediol | COG3193 |
| DN1_orf05591 | Permeases of the major facilitator superfamily | COG0477 |
| DN1_orf05595 | Permeases of the major facilitator superfamily | COG0477 |
| DN1_orf05598 | Predicted phosphatase/phosphohexomutase | COG0637 |
| DN1_orf05604 | Predicted glutamine amidotransferase | COG0121 |
| DN1_orf05605 | Diadenosine tetraphosphate (Ap4A) hydrolase and other HIT family hydrolases | COG0537 |
| DN1_orf05627 | Predicted alpha/beta hydrolase | COG4757 |
| DN1_orf05632 | Permeases of the major facilitator superfamily | COG0477 |
| DN1_orf05640 | Permeases of the major facilitator superfamily | COG0477 |
| DN1_orf05667 | Uncharacterized NAD(FAD)-dependent dehydrogenases | COG0446 |
| DN1_orf05670 | Permeases of the drug/metabolite transporter (DMT) superfamily | COG0697 |
| DN1_orf05673 | Permeases of the major facilitator superfamily | COG0477 |
| DN1_orf05697 | ABC-type protease/lipase transport system, ATPase and permease components | COG4618 |
| DN1_orf05710 | Predicted alpha/beta hydrolase | COG4757 |
| DN1_orf05717 | Permeases of the major facilitator superfamily | COG0477 |
| DN1_orf05734 | Putative NADPH-quinone reductase (modulator of drug activity B) | COG2249 |
| DN1_orf05738 | Predicted amidohydrolase | COG0388 |
| DN1_orf05759 | Predicted hydrolases or acyltransferases (alpha/beta hydrolase superfamily) | COG0596 |
| DN1_orf05760 | Pirin-related protein | COG1741 |
| DN1_orf05769 | Pirin-related protein | COG1741 |
| DN1_orf05770 | Predicted flavoprotein | COG0431 |
| DN1_orf05774 | Predicted O-methyltransferase | COG4122 |
| DN1_orf05832 | SAM-dependent methyltransferases | COG0500 |
| DN1_orf05863 | Uncharacterized protein, possibly involved in glyoxylate utilization | COG3257 |
| DN1_orf05868 | NADPH:quinone reductase and related Zn-dependent oxidoreductases | COG0604 |
| DN1_orf05871 | Putative intracellular protease/amidase | COG0693 |
| DN1_orf05875 | Permeases of the major facilitator superfamily | COG0477 |
| DN1_orf05876 | Predicted glycosyltransferases | COG1216 |
| DN1_orf05910 | Permeases of the major facilitator superfamily | COG0477 |
| DN1_orf05936 | SAM-dependent methyltransferases | COG0500 |
| DN1_orf05938 | Phenylpropionate dioxygenase and related ring-hydroxylating dioxygenases, large terminal subunit | COG4638 |
| DN1_orf05939 | Acetyltransferase (isoleucine patch superfamily) | COG0110 |
| DN1_orf05940 | Dehydrogenases with different specificities (related to short-chain alcohol dehydrogenases) | COG1028 |
| DN1_orf05974 | Dehydrogenases with different specificities (related to short-chain alcohol dehydrogenases) | COG1028 |
| DN1_orf05978 | Histone acetyltransferase HPA2 and related acetyltransferases | COG0454 |
| DN1_orf05981 | Permeases of the drug/metabolite transporter (DMT) superfamily | COG0697 |
| DN1_orf06017 | Protein related to penicillin acylase | COG2366 |
| DN1_orf06032 | Dioxygenases related to 2-nitropropane dioxygenase | COG2070 |
| DN1_orf06034 | Dehydrogenases with different specificities (related to short-chain alcohol dehydrogenases) | COG1028 |
| DN1_orf06040 | Permeases of the major facilitator superfamily | COG0477 |
| DN1_orf06052 | Metal-dependent hydrolases of the beta-lactamase superfamily I | COG1235 |
| DN1_orf06057 | Predicted permease | COG0628 |
| DN1_orf06059 | Putative Zn-dependent protease, contains TPR repeats | COG4783 |
| DN1_orf06065 | Zn-dependent hydrolases, including glyoxylases | COG0491 |
| DN1_orf06079 | Predicted esterase of the alpha-beta hydrolase superfamily | COG1752 |
| DN1_orf06099 | Plasmid stabilization system protein | COG3668 |
| DN1_orf06103 | Dehydrogenases with different specificities (related to short-chain alcohol dehydrogenases) | COG1028 |
| DN1_orf06104 | Dehydrogenases with different specificities (related to short-chain alcohol dehydrogenases) | COG1028 |
| DN1_orf06105 | Dehydrogenases with different specificities (related to short-chain alcohol dehydrogenases) | COG1028 |
| DN1_orf06110 | Zn-dependent hydrolases, including glyoxylases | COG0491 |
| DN1_orf06116 | Predicted PP-loop superfamily ATPase | COG0603 |
| DN1_orf06125 | Predicted thioesterase | COG0824 |
| DN1_orf06148 | Multimeric flavodoxin WrbA | COG0655 |
| DN1_orf06166 | Predicted pyrophosphatase | COG1694 |
| DN1_orf06179 | Lactate dehydrogenase and related dehydrogenases | COG1052 |
| DN1_orf06182 | Predicted hydrolase of alkaline phosphatase superfamily | COG3083 |
| DN1_orf06231 | Predicted deacylase | COG3608 |
| DN1_orf06248 | Uncharacterized protein involved in propionate catabolism | COG2079 |
| DN1_orf06268 | Predicted periplasmic protein | COG3895 |
| DN1_orf06272 | 4-hydroxyphenylpyruvate dioxygenase and related hemolysins | COG3185 |
| DN1_orf06275 | NADPH:quinone reductase and related Zn-dependent oxidoreductases | COG0604 |
| DN1_orf06285 | Predicted sulfurtransferase | COG1054 |
| DN1_orf06291 | Putative NADPH-quinone reductase (modulator of drug activity B) | COG2249 |
| DN1_orf06322 | Zn-dependent hydrolases, including glyoxylases | COG0491 |
| DN1_orf06327 | Predicted metal-dependent hydrolase | COG3687 |
| DN1_orf06328 | Predicted hydrolases or acyltransferases (alpha/beta hydrolase superfamily) | COG0596 |
| DN1_orf06339 | Predicted dioxygenase of extradiol dioxygenase family | COG3565 |
| DN1_orf06354 | Predicted hydrolase (HAD superfamily) | COG1011 |
| DN1_orf06365 | Aldo/keto reductases, related to diketogulonate reductase | COG0656 |
| DN1_orf06375 | SAM-dependent methyltransferases | COG0500 |
| DN1_orf06384 | Uncharacterized protein involved in propionate catabolism | COG2079 |
| DN1_orf06387 | Predicted permeases | COG0730 |
| DN1_orf06398 | Predicted ATPase | COG4637 |
| DN1_orf06404 | Na+/proline symporter | COG0591 |
| DN1_orf06417 | SAM-dependent methyltransferases | COG0500 |
| DN1_orf06419 | SAM-dependent methyltransferases | COG0500 |
| DN1_orf06424 | GTPase | COG1159 |
| DN1_orf06440 | Predicted aminomethyltransferase related to GcvT | COG0354 |
| DN1_orf06453 | Putative ammonia monooxygenase | COG3180 |
| DN1_orf06465 | Putative NADH-flavin reductase | COG2910 |
| DN1_orf06479 | Predicted hydrolases or acyltransferases (alpha/beta hydrolase superfamily) | COG0596 |
| DN1_orf06483 | Predicted acetyltransferase | COG3153 |
| DN1_orf06488 | Acetyltransferase (isoleucine patch superfamily) | COG0110 |
| DN1_orf06491 | Acetyltransferase (isoleucine patch superfamily) | COG0110 |
| DN1_orf06494 | Permeases of the major facilitator superfamily | COG0477 |
| DN1_orf06592 | Predicted ATPase | COG0433 |
| DN1_orf06600 | Predicted deacetylase | COG3233 |
| DN1_orf06613 | MoxR-like ATPases | COG0714 |
| DN1_orf06616 | Uncharacterized conserved protein (some members contain a von Willebrand factor type A (vWA) domain) | COG1721 |
| DN1_orf06637 | Uncharacterized protein related to arylsulfate sulfotransferase involved in siderophore biosynthesis | COG4321 |
| DN1_orf06638 | Putative intracellular protease/amidase | COG0693 |
| DN1_orf06650 | Permeases of the major facilitator superfamily | COG0477 |
| DN1_orf06652 | Metal-dependent amidase/aminoacylase/carboxypeptidase | COG1473 |
| DN1_orf06659 | Putative hemolysin | COG3176 |
| DN1_orf06679 | Lysine efflux permease | COG1279 |
| DN1_orf06689 | Predicted periplasmic lipoprotein | COG3489 |
| DN1_orf06702 | PAP2 (acid phosphatase) superfamily protein | COG3907 |
| DN1_orf06709 | Protein affecting phage T7 exclusion by the F plasmid | COG3030 |
| DN1_orf06710 | Dehydrogenases with different specificities (related to short-chain alcohol dehydrogenases) | COG1028 |
| DN1_orf06715 | Permeases of the major facilitator superfamily | COG0477 |
| DN1_orf06758 | Predicted methyltransferases | COG0313 |
| DN1_orf06759 | Putative lipoprotein | COG3107 |
| DN1_orf06763 | Predicted periplasmic or secreted lipoprotein | COG2823 |
| DN1_orf06764 | Stringent starvation protein B | COG2969 |
| DN1_orf06778 | Predicted ATPase | COG1485 |
| DN1_orf06781 | Predicted hydrolase of the alpha/beta superfamily | COG2945 |
| DN1_orf06803 | Low specificity phosphatase (HAD superfamily) | COG1778 |
| DN1_orf06806 | ABC-type (unclassified) transport system, ATPase component | COG1137 |
| DN1_orf06811 | Predicted P-loop-containing kinase | COG1660 |
| DN1_orf06823 | Bacteriophage capsid portal protein | COG5518 |
| DN1_orf06836 | Phage-related lysozyme (muraminidase) | COG3772 |
| DN1_orf06845 | Phage-related tail fibre protein | COG5301 |
| DN1_orf06859 | Predicted Zn-dependent proteases and their inactivated homologs | COG0312 |
| DN1_orf06862 | Predicted Zn-dependent proteases and their inactivated homologs | COG0312 |
| DN1_orf06863 | Predicted amidohydrolase | COG0388 |
| DN1_orf06883 | Large extracellular alpha-helical protein | COG2373 |
| DN1_orf06916 | Uncharacterized proteins, homologs of lactam utilization protein B | COG1540 |
| DN1_orf06922 | FOG: TPR repeat, SEL1 subfamily | COG0790 |
| DN1_orf06923 | Predicted membrane-associated, metal-dependent hydrolase | COG2194 |
| DN1_orf06947 | Acetyltransferases | COG0456 |
| DN1_orf06952 | Predicted ATPase | COG1485 |
| DN1_orf06970 | Nucleoid-associated protein | COG3081 |
| DN1_orf07051 | DNA uptake lipoprotein | COG4105 |
| DN1_orf07075 | Uncharacterized membrane protein, putative virulence factor | COG0728 |
| DN1_orf07081 | Predicted GTPase | COG0536 |
| DN1_orf07107 | Predicted nucleotidyltransferase | COG3541 |
| DN1_orf07123 | ATPase components of ABC transporters with duplicated ATPase domains | COG0488 |
| DN1_orf07142 | Putative GTPases (G3E family) | COG0523 |
| DN1_orf07154 | FOG: Ankyrin repeat | COG0666 |
| DN1_orf07162 | Uncharacterized MobA-related protein | COG2068 |
| DN1_orf07170 | Permeases of the major facilitator superfamily | COG0477 |
| DN1_orf07179 | Lactate dehydrogenase and related dehydrogenases | COG1052 |
| DN1_orf07198 | Predicted dehydrogenase | COG0579 |
| DN1_orf07215 | Permeases of the major facilitator superfamily | COG0477 |
| DN1_orf07218 | Predicted nucleoside-diphosphate sugar epimerase | COG1090 |
| DN1_orf07219 | Predicted NAD/FAD-dependent oxidoreductase | COG3380 |
| DN1_orf07232 | FOG: TPR repeat | COG0457 |
| DN1_orf07247 | Acetyltransferases | COG0456 |
| DN1_orf07263 | Paraquat-inducible protein B | COG3008 |
| DN1_orf07267 | Sulfite oxidase and related enzymes | COG2041 |
| DN1_orf07292 | Ferredoxin subunits of nitrite reductase and ring-hydroxylating dioxygenases | COG2146 |
| DN1_orf07295 | Predicted metal-binding protein | COG3019 |
| DN1_orf07298 | Predicted epimerase, PhzC/PhzF homolog | COG0384 |
| DN1_orf07299 | Predicted metalloprotease | COG2321 |
| DN1_orf07301 | Permeases | COG2252 |
| DN1_orf07304 | DNA-binding protein, stimulates sugar fermentation | COG1489 |
| DN1_orf07312 | Na+/proline symporter | COG0591 |
| DN1_orf07332 | Predicted periplasmic or secreted lipoprotein | COG2823 |
| DN1_orf07388 | Permeases of the drug/metabolite transporter (DMT) superfamily | COG0697 |
| DN1_orf07394 | Permeases of the drug/metabolite transporter (DMT) superfamily | COG0697 |
| DN1_orf07398 | Dehydrogenases with different specificities (related to short-chain alcohol dehydrogenases) | COG1028 |
| DN1_orf07403 | Predicted pyrophosphatase | COG1694 |
| DN1_orf07404 | SAM-dependent methyltransferases | COG0500 |
| DN1_orf07409 | Predicted peroxiredoxins | COG2044 |
| DN1_orf07417 | Predicted amidohydrolase | COG0388 |
| DN1_orf07441 | Predicted acetyltransferases and hydrolases with the alpha/beta hydrolase fold | COG1075 |
| DN1_orf07449 | Predicted Fe-S-cluster oxidoreductase | COG0727 |
| DN1_orf07471 | Short-chain dehydrogenases of various substrate specificities | COG0300 |
| DN1_orf07472 | Predicted membrane protein, hemolysin III homolog | COG1272 |
| DN1_orf07474 | Permeases of the drug/metabolite transporter (DMT) superfamily | COG0697 |
| DN1_orf07477 | SAM-dependent methyltransferases | COG0500 |
| DN1_orf07484 | NTP pyrophosphohydrolases including oxidative damage repair enzymes | COG0494 |
| DN1_orf07520 | Uncharacterized ABC-type transport system, ATPase component | COG4674 |
| DN1_orf07522 | Histone acetyltransferase HPA2 and related acetyltransferases | COG0454 |
| DN1_orf07539 | Plasmid stabilization system protein | COG3668 |
| DN1_orf07545 | Sulfite oxidase and related enzymes | COG2041 |
| DN1_orf07552 | Permeases of the major facilitator superfamily | COG0477 |
| DN1_orf07572 | Permeases of the major facilitator superfamily | COG0477 |
| DN1_orf07577 | Permeases of the major facilitator superfamily | COG0477 |
| DN1_orf07578 | Phenylpropionate dioxygenase and related ring-hydroxylating dioxygenases, large terminal subunit | COG4638 |
| DN1_orf07582 | Short-chain alcohol dehydrogenase of unknown specificity | COG4221 |
| DN1_orf07599 | Phospholipase/lecithinase/hemolysin | COG3240 |
| DN1_orf07602 | Predicted Rossmann fold nucleotide-binding protein | COG1611 |
| DN1_orf07633 | GTPases | COG2262 |
| DN1_orf07634 | Uncharacterized host factor I protein | COG1923 |
| DN1_orf07642 | Predicted ATPase or kinase | COG0802 |
| DN1_orf07647 | Predicted GTPases | COG1162 |
| DN1_orf07662 | Uncharacterized membrane protein affecting hemolysin expression | COG3726 |
| DN1_orf07663 | Predicted membrane-bound metal-dependent hydrolases | COG1988 |
| DN1_orf07669 | Predicted aspartyl protease | COG3577 |
| DN1_orf07672 | Predicted esterase | COG3150 |
| DN1_orf07674 | Predicted phosphohydrolases | COG1409 |
| DN1_orf07676 | NTP pyrophosphohydrolases including oxidative damage repair enzymes | COG0494 |
| DN1_orf07681 | Putative NADPH-quinone reductase (modulator of drug activity B) | COG2249 |
| DN1_orf07733 | Serine/threonine protein kinase | COG0515 |
| DN1_orf07753 | Protein involved in catabolism of external DNA | COG2961 |
| DN1_orf07765 | Predicted permeases | COG0730 |
| DN1_orf07773 | Permeases of the major facilitator superfamily | COG0477 |
| DN1_orf07775 | Dehydrogenases with different specificities (related to short-chain alcohol dehydrogenases) | COG1028 |
| DN1_orf07779 | NADPH-dependent glutamate synthase beta chain and related oxidoreductases | COG0493 |
| DN1_orf07800 | Putative Zn-dependent protease | COG4784 |
| DN1_orf07814 | Predicted hydrolases or acyltransferases (alpha/beta hydrolase superfamily) | COG0596 |
| DN1_orf07824 | Predicted unusual protein kinase | COG0661 |
| DN1_orf07844 | Predicted hydrolases or acyltransferases (alpha/beta hydrolase superfamily) | COG0596 |
| DN1_orf07877 | Cytosine deaminase and related metal-dependent hydrolases | COG0402 |
| DN1_orf07886 | Phospholipase/lecithinase/hemolysin | COG3240 |
| DN1_orf07916 | Predicted metal-dependent membrane protease | COG1266 |
| DN1_orf07935 | Predicted dehydrogenase | COG0579 |
| DN1_orf07942 | Predicted dehydrogenases and related proteins | COG0673 |
| DN1_orf07944 | Dehydrogenases with different specificities (related to short-chain alcohol dehydrogenases) | COG1028 |
| DN1_orf07958 | Permeases of the major facilitator superfamily | COG0477 |
| DN1_orf07986 | NTP pyrophosphohydrolases including oxidative damage repair enzymes | COG0494 |
| DN1_orf07987 | Predicted hydrolase (HAD superfamily) | COG1011 |
| DN1_orf07999 | Predicted thioesterase | COG0824 |
| DN1_orf08050 | Permeases of the major facilitator superfamily | COG0477 |
| DN1_orf08069 | NADPH:quinone reductase and related Zn-dependent oxidoreductases | COG0604 |
| DN1_orf08097 | ATPase components of ABC transporters with duplicated ATPase domains | COG0488 |
| DN1_orf08137 | Predicted hydrolase (HAD superfamily) | COG1011 |
| DN1_orf08186 | Permeases of the major facilitator superfamily | COG0477 |
| DN1_orf08191 | Predicted enzyme of the cupin superfamily | COG3450 |
| DN1_orf08215 | Predicted thioesterase | COG0824 |
| DN1_orf08240 | Uncharacterized NAD(FAD)-dependent dehydrogenases | COG0446 |
| DN1_orf08244 | Uncharacterized protein, possibly involved in utilization of glycolate and propanediol | COG3193 |
| DN1_orf08258 | Hemolysins and related proteins containing CBS domains | COG1253 |
| DN1_orf08268 | ABC-type uncharacterized transport system, permease component | COG4590 |
| DN1_orf08270 | Permeases of the major facilitator superfamily | COG0477 |
| DN1_orf08292 | Predicted thioesterase | COG0824 |
| DN1_orf08327 | Phenylpropionate dioxygenase and related ring-hydroxylating dioxygenases, large terminal subunit | COG4638 |
| DN1_orf08344 | Threonine dehydrogenase and related Zn-dependent dehydrogenases | COG1063 |
| DN1_orf08354 | Zn-dependent alcohol dehydrogenases | COG1064 |
| DN1_orf08360 | Predicted transporter component | COG2391 |
| DN1_orf08362 | Histone acetyltransferase HPA2 and related acetyltransferases | COG0454 |
| DN1_orf08421 | Predicted permeases | COG0679 |
| DN1_orf08436 | Predicted Zn-dependent peptidases | COG0612 |
| DN1_orf08437 | Histone acetyltransferase HPA2 and related acetyltransferases | COG0454 |
| DN1_orf08438 | Permeases of the major facilitator superfamily | COG0477 |
| DN1_orf08442 | Uncharacterized membrane protein, required for spore maturation in B.subtilis. | COG2715 |
| DN1_orf08454 | Metal-dependent hydrolase | COG3568 |
| DN1_orf08459 | Predicted GTPase | COG0218 |
| DN1_orf08463 | Putative homoserine kinase type II (protein kinase fold) | COG2334 |
| DN1_orf08488 | Predicted hydrolases or acyltransferases (alpha/beta hydrolase superfamily) | COG0596 |
| DN1_orf08498 | Dehydrogenases with different specificities (related to short-chain alcohol dehydrogenases) | COG1028 |
| DN1_orf08502 | Dehydrogenases with different specificities (related to short-chain alcohol dehydrogenases) | COG1028 |
| DN1_orf08511 | Permeases of the major facilitator superfamily | COG0477 |
| DN1_orf08514 | Putative GTPases (G3E family) | COG0523 |
| DN1_orf08518 | Putative GTPases (G3E family) | COG0523 |
| DN1_orf08523 | Carbonic anhydrases/acetyltransferases, isoleucine patch superfamily | COG0663 |
| DN1_orf08527 | TRAP-type uncharacterized transport system, fused permease components | COG4666 |
| DN1_orf08530 | TRAP-type uncharacterized transport system, periplasmic component | COG2358 |
| DN1_orf08563 | Predicted GTPase | COG0486 |
| plasmid_orf00135 | Predicted Fe-S-cluster oxidoreductase | COG0727 |
| plasmid_orf00382 | MoxR-like ATPases | COG0714 |
| plasmid_orf00421 | Arylsulfatase regulator (Fe-S oxidoreductase) | COG0641 |
| plasmid_orf00422 | Arylsulfatase regulator (Fe-S oxidoreductase) | COG0641 |
| plasmid_orf00423 | Arylsulfatase regulator (Fe-S oxidoreductase) | COG0641 |
| plasmid_orf00429 | Arylsulfatase regulator (Fe-S oxidoreductase) | COG0641 |
| plasmid_orf00438 | Arylsulfatase regulator (Fe-S oxidoreductase) | COG0641 |
| plasmid_orf00443 | Arylsulfatase regulator (Fe-S oxidoreductase) | COG0641 |
| plasmid_orf00445 | Arylsulfatase regulator (Fe-S oxidoreductase) | COG0641 |
