# Supplementary material for: Genome Sequence and Metabolic Analysis of a Fluoranthene-Degrading Strain Pseudomonas aeruginosa DN1
Source: Front Microbiol. 2018 Oct 31;9:2595. doi: 10.3389/fmicb.2018.02595 (PMC6220107; doi:10.3389/fmicb.2018.02595)
Supplement: Supplementary file 6 [file Table_6.DOCX]

**Table S6 | Inorganic ion transport and metabolism**

| **Locus Tag** | **Gene Product Name** | **Function ID** |
| --- | --- | --- |
| DN1_orf00020 | K+ transport systems, NAD-binding component | COG0569 |
| DN1_orf00038 | Sulfate permease and related transporters (MFS superfamily) | COG0659 |
| DN1_orf00040 | Arylsulfatase A and related enzymes | COG3119 |
| DN1_orf00154 | Carbonic anhydrase | COG0288 |
| DN1_orf00155 | Sulfate permease and related transporters (MFS superfamily) | COG0659 |
| DN1_orf00187 | Uncharacterized Zn-ribbon-containing protein involved in phosphonate metabolism | COG2824 |
| DN1_orf00219 | Fe2+-dicitrate sensor, membrane component | COG3712 |
| DN1_orf00221 | Outer membrane receptor proteins, mostly Fe transport | COG1629 |
| DN1_orf00269 | Arylsulfatase A and related enzymes | COG3119 |
| DN1_orf00272 | ABC-type nitrate/sulfonate/bicarbonate transport system, ATPase component | COG1116 |
| DN1_orf00273 | ABC-type nitrate/sulfonate/bicarbonate transport system, permease component | COG0600 |
| DN1_orf00274 | ABC-type nitrate/sulfonate/bicarbonate transport systems, periplasmic components | COG0715 |
| DN1_orf00280 | Outer membrane receptor proteins, mostly Fe transport | COG1629 |
| DN1_orf00329 | Permeases of the major facilitator superfamily | COG0477 |
| DN1_orf00338 | Permeases of the major facilitator superfamily | COG0477 |
| DN1_orf00348 | Permeases of the major facilitator superfamily | COG0477 |
| DN1_orf00356 | Permeases of the major facilitator superfamily | COG0477 |
| DN1_orf00403 | Cyanate permease | COG2807 |
| DN1_orf00413 | ABC-type sulfate/molybdate transport systems, ATPase component | COG1118 |
| DN1_orf00415 | ABC-type sulfate transport system, permease component | COG4208 |
| DN1_orf00418 | ABC-type sulfate transport system, periplasmic component | COG1613 |
| DN1_orf00499 | Permeases of the major facilitator superfamily | COG0477 |
| DN1_orf00588 | Co/Zn/Cd efflux system component | COG1230 |
| DN1_orf00641 | Outer membrane receptor proteins, mostly Fe transport | COG1629 |
| DN1_orf00669 | Phosphate/sulphate permeases | COG0306 |
| DN1_orf00683 | Permeases of the major facilitator superfamily | COG0477 |
| DN1_orf00706 | Outer membrane receptor proteins, mostly Fe transport | COG1629 |
| DN1_orf00707 | Fe2+-dicitrate sensor, membrane component | COG3712 |
| DN1_orf00782 | Nitric oxide reductase large subunit | COG3256 |
| DN1_orf00785 | Nitric oxide reductase activation protein | COG4548 |
| DN1_orf00810 | Predicted ferric reductase | COG4097 |
| DN1_orf00878 | Rhodanese-related sulfurtransferase | COG0607 |
| DN1_orf00880 | Uncharacterized protein affecting Mg2+/Co2+ transport | COG2967 |
| DN1_orf01006 | Catalase | COG0753 |
| DN1_orf01008 | Bacterioferritin (cytochrome b1) | COG2193 |
| DN1_orf01012 | Permeases of the major facilitator superfamily | COG0477 |
| DN1_orf01038 | Outer membrane receptor for ferric coprogen and ferric-rhodotorulic acid | COG4773 |
| DN1_orf01043 | Permeases of the major facilitator superfamily | COG0477 |
| DN1_orf01122 | Permeases of the major facilitator superfamily | COG0477 |
| DN1_orf01128 | Outer membrane receptor proteins, mostly Fe transport | COG1629 |
| DN1_orf01129 | Fe2+-dicitrate sensor, membrane component | COG3712 |
| DN1_orf01147 | Outer membrane receptor for ferrienterochelin and colicins | COG4771 |
| DN1_orf01277 | Permeases of the major facilitator superfamily | COG0477 |
| DN1_orf01316 | Siderophore-interacting protein | COG2375 |
| DN1_orf01343 | Fe2+-dicitrate sensor, membrane component | COG3712 |
| DN1_orf01344 | Cyanate lyase | COG1513 |
| DN1_orf01346 | Carbonic anhydrase | COG0288 |
| DN1_orf01353 | Outer membrane receptor proteins, mostly Fe transport | COG1629 |
| DN1_orf01365 | Uncharacterized protein involved in copper resistance | COG3667 |
| DN1_orf01371 | Permeases of the major facilitator superfamily | COG0477 |
| DN1_orf01376 | Outer membrane receptor proteins, mostly Fe transport | COG1629 |
| DN1_orf01400 | Phenylpropionate dioxygenase and related ring-hydroxylating dioxygenases, large terminal subunit | COG4638 |
| DN1_orf01407 | Outer membrane receptor proteins, mostly Fe transport | COG1629 |
| DN1_orf01415 | Fe2+-dicitrate sensor, membrane component | COG3712 |
| DN1_orf01419 | Predicted flavoprotein involved in K+ transport | COG2072 |
| DN1_orf01435 | Permeases of the major facilitator superfamily | COG0477 |
| DN1_orf01470 | NhaP-type Na+/H+ and K+/H+ antiporters | COG0025 |
| DN1_orf01491 | Catalase | COG0753 |
| DN1_orf01577 | Permeases of the major facilitator superfamily | COG0477 |
| DN1_orf01582 | Permeases of the major facilitator superfamily | COG0477 |
| DN1_orf01611 | Predicted flavoprotein involved in K+ transport | COG2072 |
| DN1_orf01677 | Permeases of the major facilitator superfamily | COG0477 |
| DN1_orf01700 | Na+/H+ antiporter NhaD and related arsenite permeases | COG1055 |
| DN1_orf01716 | Outer membrane receptor proteins, mostly Fe transport | COG1629 |
| DN1_orf01746 | ABC-type nitrate/sulfonate/bicarbonate transport system, permease component | COG0600 |
| DN1_orf01747 | ABC-type nitrate/sulfonate/bicarbonate transport system, ATPase component | COG1116 |
| DN1_orf01748 | ABC-type nitrate/sulfonate/bicarbonate transport systems, periplasmic components | COG0715 |
| DN1_orf01776 | ABC-type nitrate/sulfonate/bicarbonate transport system, permease component | COG0600 |
| DN1_orf01778 | ABC-type nitrate/sulfonate/bicarbonate transport systems, periplasmic components | COG0715 |
| DN1_orf01779 | ABC-type nitrate/sulfonate/bicarbonate transport system, ATPase component | COG1116 |
| DN1_orf01809 | ABC-type metal ion transport system, periplasmic component/surface antigen | COG1464 |
| DN1_orf01810 | ABC-type metal ion transport system, ATPase component | COG1135 |
| DN1_orf01812 | ABC-type metal ion transport system, permease component | COG2011 |
| DN1_orf01847 | ABC-type Fe3+ transport system, periplasmic component | COG1840 |
| DN1_orf01859 | Fe2+/Zn2+ uptake regulation proteins | COG0735 |
| DN1_orf01865 | Fe2+-dicitrate sensor, membrane component | COG3712 |
| DN1_orf01876 | ABC-type siderophore export system, fused ATPase and permease components | COG4615 |
| DN1_orf01878 | Outer membrane receptor for ferric coprogen and ferric-rhodotorulic acid | COG4773 |
| DN1_orf01895 | ABC-type metal ion transport system, periplasmic component/surface adhesin | COG0803 |
| DN1_orf01896 | ABC-type Mn/Zn transport systems, ATPase component | COG1121 |
| DN1_orf01897 | ABC-type Mn2+/Zn2+ transport systems, permease components | COG1108 |
| DN1_orf01898 | ABC-type metal ion transport system, periplasmic component/surface adhesin | COG0803 |
| DN1_orf01945 | Cation transport ATPase | COG2217 |
| DN1_orf01975 | Enterochelin esterase and related enzymes | COG2382 |
| DN1_orf01997 | Outer membrane receptor proteins, mostly Fe transport | COG1629 |
| DN1_orf01998 | Fe2+-dicitrate sensor, membrane component | COG3712 |
| DN1_orf02004 | Permeases of the major facilitator superfamily | COG0477 |
| DN1_orf02045 | Cyanate permease | COG2807 |
| DN1_orf02061 | Phenylpropionate dioxygenase and related ring-hydroxylating dioxygenases, large terminal subunit | COG4638 |
| DN1_orf02071 | Phenylpropionate dioxygenase and related ring-hydroxylating dioxygenases, large terminal subunit | COG4638 |
| DN1_orf02077 | Putative silver efflux pump | COG3696 |
| DN1_orf02127 | Membrane protein TerC, possibly involved in tellurium resistance | COG0861 |
| DN1_orf02150 | Sulfate permease and related transporters (MFS superfamily) | COG0659 |
| DN1_orf02260 | Outer membrane receptor proteins, mostly Fe transport | COG1629 |
| DN1_orf02264 | ABC-type nitrate/sulfonate/bicarbonate transport systems, periplasmic components | COG0715 |
| DN1_orf02266 | ABC-type nitrate/sulfonate/bicarbonate transport systems, periplasmic components | COG0715 |
| DN1_orf02267 | ABC-type nitrate/sulfonate/bicarbonate transport systems, periplasmic components | COG0715 |
| DN1_orf02273 | ABC-type nitrate/sulfonate/bicarbonate transport systems, periplasmic components | COG0715 |
| DN1_orf02278 | Rhodanese-related sulfurtransferase | COG0607 |
| DN1_orf02345 | Uncharacterized conserved protein involved in intracellular sulfur reduction | COG1553 |
| DN1_orf02346 | Uncharacterized protein involved in the oxidation of intracellular sulfur | COG2923 |
| DN1_orf02347 | Uncharacterized conserved protein involved in oxidation of intracellular sulfur | COG2168 |
| DN1_orf02348 | Dissimilatory sulfite reductase (desulfoviridin), gamma subunit | COG2920 |
| DN1_orf02405 | NADH:ubiquinone oxidoreductase subunit 5 (chain L)/Multisubunit Na+/H+ antiporter, MnhA subunit | COG1009 |
| DN1_orf02430 | Uncharacterized protein involved in response to NO | COG3213 |
| DN1_orf02470 | Outer membrane receptor for ferrienterochelin and colicins | COG4771 |
| DN1_orf02486 | Permeases of the major facilitator superfamily | COG0477 |
| DN1_orf02511 | Fe2+-dicitrate sensor, membrane component | COG3712 |
| DN1_orf02588 | Predicted iron-dependent peroxidase | COG2837 |
| DN1_orf02605 | Formate/nitrite family of transporters | COG2116 |
| DN1_orf02644 | Uncharacterized copper-binding protein | COG4454 |
| DN1_orf02689 | Permeases of the major facilitator superfamily | COG0477 |
| DN1_orf02800 | Sulfite reductase, beta subunit (hemoprotein) | COG0155 |
| DN1_orf02810 | Outer membrane receptor proteins, mostly Fe transport | COG1629 |
| DN1_orf02811 | ABC-type cobalamin/Fe3+-siderophores transport systems, ATPase components | COG1120 |
| DN1_orf02812 | ABC-type Fe3+-hydroxamate transport system, periplasmic component | COG0614 |
| DN1_orf02815 | ABC-type Fe3+-siderophore transport system, permease component | COG0609 |
| DN1_orf02979 | Cytochrome c peroxidase | COG1858 |
| DN1_orf02988 | Ammonia permease | COG0004 |
| DN1_orf03127 | Permeases of the major facilitator superfamily | COG0477 |
| DN1_orf03200 | P pilus assembly/Cpx signaling pathway, periplasmic inhibitor/zinc-resistance associated protein | COG3678 |
| DN1_orf03205 | Trk-type K+ transport systems, membrane components | COG0168 |
| DN1_orf03266 | ABC-type Fe3+ transport system, periplasmic component | COG1840 |
| DN1_orf03288 | Membrane transporters of cations and cationic drugs | COG2076 |
| DN1_orf03293 | Outer membrane receptor for Fe3+-dicitrate | COG4772 |
| DN1_orf03310 | Phosphate-selective porin | COG3746 |
| DN1_orf03313 | Phosphate-selective porin | COG3746 |
| DN1_orf03334 | Alkaline phosphatase | COG1785 |
| DN1_orf03369 | ABC-type phosphate/phosphonate transport system, periplasmic component | COG3221 |
| DN1_orf03370 | ABC-type phosphate/phosphonate transport system, ATPase component | COG3638 |
| DN1_orf03371 | ABC-type phosphate/phosphonate transport system, permease component | COG3639 |
| DN1_orf03372 | ABC-type phosphate/phosphonate transport system, permease component | COG3639 |
| DN1_orf03429 | Permeases of the major facilitator superfamily | COG0477 |
| DN1_orf03455 | Uncharacterized component of phosphonate metabolism | COG3709 |
| DN1_orf03456 | Metal-dependent hydrolase involved in phosphonate metabolism | COG3454 |
| DN1_orf03458 | ABC-type phosphonate transport system, ATPase component | COG4778 |
| DN1_orf03459 | ABC-type phosphonate transport system, ATPase component | COG4107 |
| DN1_orf03461 | Uncharacterized enzyme of phosphonate metabolism | COG3627 |
| DN1_orf03463 | Uncharacterized enzyme of phosphonate metabolism | COG3626 |
| DN1_orf03464 | Uncharacterized enzyme of phosphonate metabolism | COG3625 |
| DN1_orf03465 | Uncharacterized enzyme of phosphonate metabolism | COG3624 |
| DN1_orf03468 | ABC-type phosphate/phosphonate transport system, permease component | COG3639 |
| DN1_orf03469 | ABC-type phosphate/phosphonate transport system, periplasmic component | COG3221 |
| DN1_orf03471 | ABC-type phosphate/phosphonate transport system, ATPase component | COG3638 |
| DN1_orf03483 | Nitrous oxidase accessory protein | COG3420 |
| DN1_orf03509 | Outer membrane receptor proteins, mostly Fe transport | COG1629 |
| DN1_orf03510 | Fe2+-dicitrate sensor, membrane component | COG3712 |
| DN1_orf03552 | ABC-type nitrate/sulfonate/bicarbonate transport system, ATPase component | COG1116 |
| DN1_orf03553 | ABC-type nitrate/sulfonate/bicarbonate transport system, permease component | COG0600 |
| DN1_orf03555 | ABC-type nitrate/sulfonate/bicarbonate transport systems, periplasmic components | COG0715 |
| DN1_orf03557 | ABC-type nitrate/sulfonate/bicarbonate transport system, ATPase component | COG1116 |
| DN1_orf03558 | ABC-type nitrate/sulfonate/bicarbonate transport system, permease component | COG0600 |
| DN1_orf03560 | ABC-type nitrate/sulfonate/bicarbonate transport systems, periplasmic components | COG0715 |
| DN1_orf03592 | Permeases of the major facilitator superfamily | COG0477 |
| DN1_orf03598 | Permeases of the major facilitator superfamily | COG0477 |
| DN1_orf03652 | Copper chaperone | COG2608 |
| DN1_orf03664 | Bacterioferritin-associated ferredoxin | COG2906 |
| DN1_orf03665 | Bacterioferritin (cytochrome b1) | COG2193 |
| DN1_orf03667 | Permeases of the major facilitator superfamily | COG0477 |
| DN1_orf03738 | Permeases of the major facilitator superfamily | COG0477 |
| DN1_orf03771 | Permeases of the major facilitator superfamily | COG0477 |
| DN1_orf03861 | NhaP-type Na+/H+ and K+/H+ antiporters | COG0025 |
| DN1_orf03865 | Arsenate reductase and related proteins, glutaredoxin family | COG1393 |
| DN1_orf03902 | Cation transport ATPase | COG2217 |
| DN1_orf03928 | Permeases of the major facilitator superfamily | COG0477 |
| DN1_orf03973 | NhaP-type Na+/H+ and K+/H+ antiporters | COG0025 |
| DN1_orf03983 | Putative Mg2+ and Co2+ transporter CorB | COG4536 |
| DN1_orf03985 | Permeases of the major facilitator superfamily | COG0477 |
| DN1_orf04065 | Outer membrane receptor proteins, mostly Fe transport | COG1629 |
| DN1_orf04135 | Di- and tricarboxylate transporters | COG0471 |
| DN1_orf04178 | Outer membrane receptor proteins, mostly Fe transport | COG1629 |
| DN1_orf04190 | Nitrate/nitrite transporter | COG2223 |
| DN1_orf04192 | Nitrate/nitrite transporter | COG2223 |
| DN1_orf04207 | NhaP-type Na+/H+ and K+/H+ antiporters | COG0025 |
| DN1_orf04227 | Fe2+-dicitrate sensor, membrane component | COG3712 |
| DN1_orf04228 | Outer membrane receptor for Fe3+-dicitrate | COG4772 |
| DN1_orf04241 | Phosphodiesterase/alkaline phosphatase D | COG3540 |
| DN1_orf04259 | Cation transport ATPase | COG2217 |
| DN1_orf04279 | ABC-type metal ion transport system, periplasmic component/surface antigen | COG1464 |
| DN1_orf04290 | ABC-type nitrate/sulfonate/bicarbonate transport system, permease component | COG0600 |
| DN1_orf04291 | ABC-type taurine transport system, ATPase component | COG4525 |
| DN1_orf04292 | ABC-type taurine transport system, periplasmic component | COG4521 |
| DN1_orf04328 | Predicted Co/Zn/Cd cation transporters | COG0053 |
| DN1_orf04356 | Putative Mg2+ and Co2+ transporter CorC | COG4535 |
| DN1_orf04445 | ABC-type Fe3+-hydroxamate transport system, periplasmic component | COG0614 |
| DN1_orf04536 | Heme oxygenase | COG3230 |
| DN1_orf04549 | Permeases of the major facilitator superfamily | COG0477 |
| DN1_orf04554 | Sulfite reductase, beta subunit (hemoprotein) | COG0155 |
| DN1_orf04565 | Permeases of the major facilitator superfamily | COG0477 |
| DN1_orf04593 | Outer membrane receptor proteins, mostly Fe transport | COG1629 |
| DN1_orf04596 | ABC-type cobalamin/Fe3+-siderophores transport systems, ATPase components | COG1120 |
| DN1_orf04597 | ABC-type Fe2+-enterobactin transport system, periplasmic component | COG4592 |
| DN1_orf04598 | ABC-type Fe3+-siderophore transport system, permease component | COG0609 |
| DN1_orf04601 | ABC-type enterobactin transport system, permease component | COG4779 |
| DN1_orf04610 | Outer membrane receptor for ferric coprogen and ferric-rhodotorulic acid | COG4773 |
| DN1_orf04627 | Putative silver efflux pump | COG3696 |
| DN1_orf04645 | Permeases of the major facilitator superfamily | COG0477 |
| DN1_orf04758 | Membrane transporters of cations and cationic drugs | COG2076 |
| DN1_orf04775 | Mg2+ and Co2+ transporters | COG0598 |
| DN1_orf04790 | ABC-type molybdate transport system, periplasmic component | COG0725 |
| DN1_orf04791 | ABC-type molybdate transport system, permease component | COG4149 |
| DN1_orf04793 | ABC-type molybdate transport system, ATPase component | COG4148 |
| DN1_orf04797 | Trk-type K+ transport systems, membrane components | COG0168 |
| DN1_orf04830 | Sulfite reductase, beta subunit (hemoprotein) | COG0155 |
| DN1_orf04853 | Na+/H+ antiporter | COG3067 |
| DN1_orf04904 | ABC-type nitrate/sulfonate/bicarbonate transport systems, periplasmic components | COG0715 |
| DN1_orf04908 | Nitrate/nitrite transporter | COG2223 |
| DN1_orf04915 | Ferredoxin subunits of nitrite reductase and ring-hydroxylating dioxygenases | COG2146 |
| DN1_orf04925 | Mg2+ and Co2+ transporters | COG0598 |
| DN1_orf05056 | Permeases of the major facilitator superfamily | COG0477 |
| DN1_orf05068 | Hemerythrin | COG2703 |
| DN1_orf05104 | Sulfate permease and related transporters (MFS superfamily) | COG0659 |
| DN1_orf05123 | K+-transporting ATPase, c chain | COG2156 |
| DN1_orf05125 | High-affinity K+ transport system, ATPase chain B | COG2216 |
| DN1_orf05128 | K+-transporting ATPase, A chain | COG2060 |
| DN1_orf05139 | Cyanate permease | COG2807 |
| DN1_orf05255 | Cation transport ATPase | COG2217 |
| DN1_orf05256 | Uncharacterized protein, possibly involved in nitrogen fixation | COG3197 |
| DN1_orf05264 | Membrane transporters of cations and cationic drugs | COG2076 |
| DN1_orf05267 | Predicted flavoprotein involved in K+ transport | COG2072 |
| DN1_orf05330 | Kef-type K+ transport systems, predicted NAD-binding component | COG1226 |
| DN1_orf05334 | ABC-type sulfate transport system, periplasmic component | COG1613 |
| DN1_orf05429 | Cation transport ATPase | COG0474 |
| DN1_orf05459 | Permeases of the major facilitator superfamily | COG0477 |
| DN1_orf05516 | Outer membrane receptor proteins, mostly Fe transport | COG1629 |
| DN1_orf05517 | Fe2+-dicitrate sensor, membrane component | COG3712 |
| DN1_orf05533 | Permeases of the major facilitator superfamily | COG0477 |
| DN1_orf05579 | Outer membrane receptor for monomeric catechols | COG4774 |
| DN1_orf05591 | Permeases of the major facilitator superfamily | COG0477 |
| DN1_orf05595 | Permeases of the major facilitator superfamily | COG0477 |
| DN1_orf05612 | Outer membrane receptor proteins, mostly Fe transport | COG1629 |
| DN1_orf05613 | Fe2+-dicitrate sensor, membrane component | COG3712 |
| DN1_orf05617 | Co/Zn/Cd efflux system component | COG1230 |
| DN1_orf05625 | Rhodanese-related sulfurtransferase | COG2897 |
| DN1_orf05632 | Permeases of the major facilitator superfamily | COG0477 |
| DN1_orf05640 | Permeases of the major facilitator superfamily | COG0477 |
| DN1_orf05673 | Permeases of the major facilitator superfamily | COG0477 |
| DN1_orf05717 | Permeases of the major facilitator superfamily | COG0477 |
| DN1_orf05761 | Rhodanese-related sulfurtransferase | COG0607 |
| DN1_orf05763 | Predicted flavoprotein involved in K+ transport | COG2072 |
| DN1_orf05766 | Kef-type K+ transport systems, predicted NAD-binding component | COG1226 |
| DN1_orf05810 | Uncharacterized protein involved in formation of periplasmic nitrate reductase | COG3062 |
| DN1_orf05817 | Membrane protein TerC, possibly involved in tellurium resistance | COG0861 |
| DN1_orf05875 | Permeases of the major facilitator superfamily | COG0477 |
| DN1_orf05910 | Permeases of the major facilitator superfamily | COG0477 |
| DN1_orf05938 | Phenylpropionate dioxygenase and related ring-hydroxylating dioxygenases, large terminal subunit | COG4638 |
| DN1_orf05982 | Multisubunit Na+/H+ antiporter, MnhG subunit | COG1320 |
| DN1_orf05983 | Multisubunit Na+/H+ antiporter, MnhF subunit | COG2212 |
| DN1_orf05984 | Multisubunit Na+/H+ antiporter, MnhE subunit | COG1863 |
| DN1_orf05986 | Multisubunit Na+/H+ antiporter, MnhC subunit | COG1006 |
| DN1_orf05987 | Formate hydrogenlyase subunit 3/Multisubunit Na+/H+ antiporter, MnhD subunit | COG0651 |
| DN1_orf05990 | NADH:ubiquinone oxidoreductase subunit 5 (chain L)/Multisubunit Na+/H+ antiporter, MnhA subunit | COG1009 |
| DN1_orf06040 | Permeases of the major facilitator superfamily | COG0477 |
| DN1_orf06102 | Kef-type K+ transport systems, predicted NAD-binding component | COG1226 |
| DN1_orf06132 | DNA-binding ferritin-like protein (oxidative damage protectant) | COG0783 |
| DN1_orf06146 | Arsenate reductase and related proteins, glutaredoxin family | COG1393 |
| DN1_orf06174 | Outer membrane receptor for ferrienterochelin and colicins | COG4771 |
| DN1_orf06194 | K+ transporter | COG3158 |
| DN1_orf06204 | Mg/Co/Ni transporter MgtE (contains CBS domain) | COG2239 |
| DN1_orf06357 | Mn2+ and Fe2+ transporters of the NRAMP family | COG1914 |
| DN1_orf06399 | Membrane transporters of cations and cationic drugs | COG2076 |
| DN1_orf06410 | Outer membrane receptor proteins, mostly Fe transport | COG1629 |
| DN1_orf06464 | Kef-type K+ transport systems, predicted NAD-binding component | COG1226 |
| DN1_orf06494 | Permeases of the major facilitator superfamily | COG0477 |
| DN1_orf06522 | ABC-type phosphate transport system, periplasmic component | COG0226 |
| DN1_orf06537 | Fe2+-dicitrate sensor, membrane component | COG3712 |
| DN1_orf06541 | Heme oxygenase | COG3230 |
| DN1_orf06562 | Chromate transport protein ChrA | COG2059 |
| DN1_orf06568 | Phosphate/sulphate permeases | COG0306 |
| DN1_orf06633 | Mn2+ and Fe2+ transporters of the NRAMP family | COG1914 |
| DN1_orf06650 | Permeases of the major facilitator superfamily | COG0477 |
| DN1_orf06670 | Fe2+ transport system protein B | COG0370 |
| DN1_orf06671 | Fe2+ transport system protein A | COG1918 |
| DN1_orf06680 | Superoxide dismutase | COG0605 |
| DN1_orf06686 | Uncharacterized iron-regulated protein | COG3487 |
| DN1_orf06711 | Putative heme iron utilization protein | COG0748 |
| DN1_orf06715 | Permeases of the major facilitator superfamily | COG0477 |
| DN1_orf06785 | Adenylylsulfate kinase and related kinases | COG0529 |
| DN1_orf06853 | Predicted divalent heavy-metal cations transporter | COG0428 |
| DN1_orf06854 | Superoxide dismutase | COG0605 |
| DN1_orf06905 | ABC-type dipeptide/oligopeptide/nickel transport systems, permease components | COG0601 |
| DN1_orf06906 | ABC-type dipeptide/oligopeptide/nickel transport systems, permease components | COG1173 |
| DN1_orf06907 | ABC-type dipeptide/oligopeptide/nickel transport system, ATPase component | COG0444 |
| DN1_orf06919 | Sulfite reductase, alpha subunit (flavoprotein) | COG0369 |
| DN1_orf06920 | Outer membrane receptor for monomeric catechols | COG4774 |
| DN1_orf07110 | Cytochrome c peroxidase | COG1858 |
| DN1_orf07156 | Catalase | COG0753 |
| DN1_orf07170 | Permeases of the major facilitator superfamily | COG0477 |
| DN1_orf07215 | Permeases of the major facilitator superfamily | COG0477 |
| DN1_orf07243 | Outer membrane receptor proteins, mostly Fe transport | COG1629 |
| DN1_orf07245 | Carbonic anhydrase | COG0288 |
| DN1_orf07259 | ABC-type Fe3+ transport system, periplasmic component | COG1840 |
| DN1_orf07261 | ABC-type Fe3+ transport system, permease component | COG1178 |
| DN1_orf07286 | ABC-type hemin transport system, ATPase component | COG4559 |
| DN1_orf07287 | ABC-type Fe3+-siderophore transport system, permease component | COG0609 |
| DN1_orf07288 | ABC-type hemin transport system, periplasmic component | COG4558 |
| DN1_orf07290 | Outer membrane receptor proteins, mostly Fe transport | COG1629 |
| DN1_orf07291 | Putative heme degradation protein | COG3720 |
| DN1_orf07292 | Ferredoxin subunits of nitrite reductase and ring-hydroxylating dioxygenases | COG2146 |
| DN1_orf07366 | Fe2+/Zn2+ uptake regulation proteins | COG0735 |
| DN1_orf07458 | Na+/phosphate symporter | COG1283 |
| DN1_orf07463 | Cation transport ATPase | COG0474 |
| DN1_orf07478 | Outer membrane receptor proteins, mostly Fe transport | COG1629 |
| DN1_orf07543 | Bacterioferritin (cytochrome b1) | COG2193 |
| DN1_orf07552 | Permeases of the major facilitator superfamily | COG0477 |
| DN1_orf07563 | Fe2+-dicitrate sensor, membrane component | COG3712 |
| DN1_orf07566 | Outer membrane receptor proteins, mostly Fe transport | COG1629 |
| DN1_orf07572 | Permeases of the major facilitator superfamily | COG0477 |
| DN1_orf07577 | Permeases of the major facilitator superfamily | COG0477 |
| DN1_orf07578 | Phenylpropionate dioxygenase and related ring-hydroxylating dioxygenases, large terminal subunit | COG4638 |
| DN1_orf07653 | Rhodanese-related sulfurtransferase | COG2897 |
| DN1_orf07707 | Membrane transporters of cations and cationic drugs | COG2076 |
| DN1_orf07759 | NhaP-type Na+/H+ and K+/H+ antiporters with a unique C-terminal domain | COG3263 |
| DN1_orf07773 | Permeases of the major facilitator superfamily | COG0477 |
| DN1_orf07841 | Periplasmic glucans biosynthesis protein | COG3131 |
| DN1_orf07871 | ABC-type phosphate/phosphonate transport system, periplasmic component | COG3221 |
| DN1_orf07895 | Rhodanese-related sulfurtransferase | COG0607 |
| DN1_orf07913 | Rhodanese-related sulfurtransferase | COG0607 |
| DN1_orf07958 | Permeases of the major facilitator superfamily | COG0477 |
| DN1_orf07985 | 3'-Phosphoadenosine 5'-phosphosulfate (PAPS) 3'-phosphatase | COG1218 |
| DN1_orf08031 | Phosphate/sulphate permeases | COG0306 |
| DN1_orf08032 | Phosphate transport regulator (distant homolog of PhoU) | COG1392 |
| DN1_orf08047 | ABC-type Fe3+ transport system, permease component | COG1178 |
| DN1_orf08048 | ABC-type Fe3+ transport system, periplasmic component | COG1840 |
| DN1_orf08050 | Permeases of the major facilitator superfamily | COG0477 |
| DN1_orf08081 | Exopolyphosphatase | COG0248 |
| DN1_orf08082 | Polyphosphate kinase | COG0855 |
| DN1_orf08090 | High-affinity Fe2+/Pb2+ permease | COG0672 |
| DN1_orf08092 | Membrane protein TerC, possibly involved in tellurium resistance | COG0861 |
| DN1_orf08120 | Mg2+ and Co2+ transporters | COG0598 |
| DN1_orf08129 | Protein implicated in iron transport, frataxin homolog | COG1965 |
| DN1_orf08148 | Ammonia permease | COG0004 |
| DN1_orf08186 | Permeases of the major facilitator superfamily | COG0477 |
| DN1_orf08263 | Phosphate uptake regulator | COG0704 |
| DN1_orf08264 | ABC-type phosphate transport system, ATPase component | COG1117 |
| DN1_orf08265 | ABC-type phosphate transport system, permease component | COG0581 |
| DN1_orf08269 | ABC-type phosphate transport system, periplasmic component | COG0226 |
| DN1_orf08270 | Permeases of the major facilitator superfamily | COG0477 |
| DN1_orf08304 | Predicted flavoprotein involved in K+ transport | COG2072 |
| DN1_orf08327 | Phenylpropionate dioxygenase and related ring-hydroxylating dioxygenases, large terminal subunit | COG4638 |
| DN1_orf08427 | Membrane protein TerC, possibly involved in tellurium resistance | COG0861 |
| DN1_orf08433 | Na+/phosphate symporter | COG1283 |
| DN1_orf08438 | Permeases of the major facilitator superfamily | COG0477 |
| DN1_orf08467 | Fe2+/Zn2+ uptake regulation proteins | COG0735 |
| DN1_orf08468 | ABC-type Zn2+ transport system, periplasmic component/surface adhesin | COG4531 |
| DN1_orf08469 | ABC-type Mn/Zn transport systems, ATPase component | COG1121 |
| DN1_orf08470 | ABC-type Mn2+/Zn2+ transport systems, permease components | COG1108 |
| DN1_orf08473 | ABC-type metal ion transport system, ATPase component | COG1135 |
| DN1_orf08475 | ABC-type metal ion transport system, permease component | COG2011 |
| DN1_orf08476 | ABC-type metal ion transport system, periplasmic component/surface antigen | COG1464 |
| DN1_orf08493 | Kef-type K+ transport systems, predicted NAD-binding component | COG1226 |
| DN1_orf08510 | Kef-type K+ transport systems, membrane components | COG0475 |
| DN1_orf08511 | Permeases of the major facilitator superfamily | COG0477 |
