# Supplementary material for: Genome Sequence and Metabolic Analysis of a Fluoranthene-Degrading Strain Pseudomonas aeruginosa DN1
Source: Front Microbiol. 2018 Oct 31;9:2595. doi: 10.3389/fmicb.2018.02595 (PMC6220107; doi:10.3389/fmicb.2018.02595)
Supplement: Supplementary file 7 [file Table_7.DOCX]

**Table S7 | Intracellular trafficking, secretion, and vesicular transport**

| **Locus Tag** | **Gene Product Name** | **Function ID** |
| --- | --- | --- |
| DN1_orf00026 | Predicted Rossmann fold nucleotide-binding protein involved in DNA uptake | COG0758 |
| DN1_orf00053 | Hemolysin activation/secretion protein | COG2831 |
| DN1_orf00059 | Large exoproteins involved in heme utilization or adhesion | COG3210 |
| DN1_orf00293 | Biopolymer transport proteins | COG0811 |
| DN1_orf00295 | Biopolymer transport protein | COG0848 |
| DN1_orf00553 | Signal recognition particle GTPase | COG0552 |
| DN1_orf00585 | Tfp pilus assembly protein, pilus retraction ATPase PilT | COG2805 |
| DN1_orf00587 | Tfp pilus assembly protein, ATPase PilU | COG5008 |
| DN1_orf00630 | Outer membrane protein | COG1538 |
| DN1_orf00998 | Preprotein translocase subunit SecY | COG0201 |
| DN1_orf01058 | Outer membrane protein | COG1538 |
| DN1_orf01151 | Biopolymer transport proteins | COG0811 |
| DN1_orf01460 | P pilus assembly protein, pilin FimA | COG3539 |
| DN1_orf01462 | P pilus assembly protein, chaperone PapD | COG3121 |
| DN1_orf01463 | P pilus assembly protein, porin PapC | COG3188 |
| DN1_orf01467 | P pilus assembly protein, chaperone PapD | COG3121 |
| DN1_orf01547 | Protease subunit of ATP-dependent Clp proteases | COG0740 |
| DN1_orf01869 | Outer membrane protein | COG1538 |
| DN1_orf01990 | Large exoproteins involved in heme utilization or adhesion | COG3210 |
| DN1_orf01991 | Hemolysin activation/secretion protein | COG2831 |
| DN1_orf02040 | Outer membrane protein | COG1538 |
| DN1_orf02080 | Outer membrane protein | COG1538 |
| DN1_orf02087 | Outer membrane protein | COG1538 |
| DN1_orf02443 | Type II secretory pathway, pseudopilin PulG | COG2165 |
| DN1_orf02445 | Type II secretory pathway, component PulJ | COG4795 |
| DN1_orf02446 | Type II secretory pathway, pseudopilin PulG | COG2165 |
| DN1_orf02447 | Type II secretory pathway, pseudopilin PulG | COG2165 |
| DN1_orf02448 | Type II secretory pathway, component PulF | COG1459 |
| DN1_orf02450 | Type II secretory pathway, ATPase PulE/Tfp pilus assembly pathway, ATPase PilB | COG2804 |
| DN1_orf02692 | Outer membrane protein | COG1538 |
| DN1_orf02881 | Tfp pilus assembly protein PilZ | COG3215 |
| DN1_orf02899 | Periplasmic serine proteases (ClpP class) | COG0616 |
| DN1_orf02909 | Biopolymer transport protein | COG0848 |
| DN1_orf02910 | Biopolymer transport proteins | COG0811 |
| DN1_orf03067 | Type II secretory pathway, component PulM | COG3149 |
| DN1_orf03070 | Type II secretory pathway, component PulL | COG3297 |
| DN1_orf03071 | Type II secretory pathway, component PulK | COG3156 |
| DN1_orf03072 | Type II secretory pathway, component PulJ | COG4795 |
| DN1_orf03075 | Type II secretory pathway, pseudopilin PulG | COG2165 |
| DN1_orf03076 | Type II secretory pathway, pseudopilin PulG | COG2165 |
| DN1_orf03077 | Type II secretory pathway, component PulF | COG1459 |
| DN1_orf03080 | Type II secretory pathway, ATPase PulE/Tfp pilus assembly pathway, ATPase PilB | COG2804 |
| DN1_orf03083 | Type II secretory pathway, component PulD | COG1450 |
| DN1_orf03097 | Tfp pilus assembly protein FimV | COG3170 |
| DN1_orf03200 | P pilus assembly/Cpx signaling pathway, periplasmic inhibitor/zinc-resistance associated protein | COG3678 |
| DN1_orf03388 | Protease subunit of ATP-dependent Clp proteases | COG0740 |
| DN1_orf03409 | Tfp pilus assembly protein FimV | COG3170 |
| DN1_orf03425 | Negative regulator of flagellin synthesis (anti-sigma28 factor) | COG2747 |
| DN1_orf03426 | Flagellar biosynthesis/type III secretory pathway chaperone | COG3418 |
| DN1_orf03500 | Outer membrane protein | COG1538 |
| DN1_orf03653 | Outer membrane protein | COG1538 |
| DN1_orf03805 | Periplasmic component of the Tol biopolymer transport system | COG0823 |
| DN1_orf03981 | Signal recognition particle GTPase | COG0541 |
| DN1_orf04086 | Tfp pilus assembly protein PilF | COG3063 |
| DN1_orf04105 | Preprotein translocase subunit SecF | COG0341 |
| DN1_orf04106 | Preprotein translocase subunit SecD | COG0342 |
| DN1_orf04108 | Preprotein translocase subunit YajC | COG1862 |
| DN1_orf04219 | Outer membrane protein | COG1538 |
| DN1_orf04492 | P pilus assembly protein, pilin FimA | COG3539 |
| DN1_orf04493 | Large exoproteins involved in heme utilization or adhesion | COG3210 |
| DN1_orf04495 | P pilus assembly protein, chaperone PapD | COG3121 |
| DN1_orf04497 | P pilus assembly protein, porin PapC | COG3188 |
| DN1_orf04498 | P pilus assembly protein, chaperone PapD | COG3121 |
| DN1_orf04499 | P pilus assembly protein, pilin FimA | COG3539 |
| DN1_orf04770 | Outer membrane protein | COG1538 |
| DN1_orf04780 | Type II secretory pathway, component PulD | COG1450 |
| DN1_orf04837 | Periplasmic serine proteases (ClpP class) | COG0616 |
| DN1_orf04883 | Protease subunit of ATP-dependent Clp proteases | COG0740 |
| DN1_orf04995 | Flagellar biosynthesis/type III secretory pathway protein | COG1317 |
| DN1_orf04997 | Type III secretory pathway, lipoprotein EscJ | COG4669 |
| DN1_orf05007 | Type II secretory pathway, component PulD | COG1450 |
| DN1_orf05022 | Type III secretory pathway, component EscV | COG4789 |
| DN1_orf05032 | Flagellar biosynthesis/type III secretory pathway ATPase | COG1157 |
| DN1_orf05036 | Flagellar motor switch/type III secretory pathway protein | COG1886 |
| DN1_orf05038 | Type III secretory pathway, component EscR | COG4790 |
| DN1_orf05040 | Type III secretory pathway, component EscS | COG4794 |
| DN1_orf05041 | Type III secretory pathway, component EscT | COG4791 |
| DN1_orf05043 | Type III secretory pathway, component EscU | COG4792 |
| DN1_orf05359 | Heme exporter protein D | COG3114 |
| DN1_orf05392 | Flagellar biosynthesis pathway, component FlhA | COG1298 |
| DN1_orf05396 | Flagellar biosynthesis pathway, component FlhB | COG1377 |
| DN1_orf05398 | Flagellar biosynthesis pathway, component FliR | COG1684 |
| DN1_orf05399 | Flagellar biosynthesis pathway, component FliQ | COG1987 |
| DN1_orf05401 | Flagellar biosynthesis pathway, component FliP | COG1338 |
| DN1_orf05405 | Flagellar motor switch/type III secretory pathway protein | COG1886 |
| DN1_orf05610 | Signal peptidase I | COG0681 |
| DN1_orf05693 | Outer membrane protein | COG1538 |
| DN1_orf05712 | Outer membrane protein | COG1538 |
| DN1_orf05913 | Flagellar biosynthesis chaperone | COG2882 |
| DN1_orf05915 | Flagellar biosynthesis/type III secretory pathway ATPase | COG1157 |
| DN1_orf05916 | Flagellar biosynthesis/type III secretory pathway protein | COG1317 |
| DN1_orf05919 | Flagellar biosynthesis/type III secretory pathway lipoprotein | COG1766 |
| DN1_orf05921 | Flagellar hook-basal body protein | COG1677 |
| DN1_orf05928 | Flagellin-specific chaperone FliS | COG1516 |
| DN1_orf05929 | Flagellin-specific chaperone FliS | COG1516 |
| DN1_orf06014 | Preprotein translocase subunit SecA (ATPase, RNA helicase) | COG0653 |
| DN1_orf06074 | P pilus assembly protein, porin PapC | COG3188 |
| DN1_orf06076 | P pilus assembly protein, pilin FimA | COG3539 |
| DN1_orf06121 | Periplasmic component of the Tol biopolymer transport system | COG0823 |
| DN1_orf06123 | Biopolymer transport protein | COG0848 |
| DN1_orf06124 | Biopolymer transport proteins | COG0811 |
| DN1_orf06190 | Type IV secretory pathway, VirJ component | COG3946 |
| DN1_orf06428 | Signal peptidase I | COG0681 |
| DN1_orf06508 | Biopolymer transport protein | COG0848 |
| DN1_orf06510 | Biopolymer transport proteins | COG0811 |
| DN1_orf06513 | Hemolysin activation/secretion protein | COG2831 |
| DN1_orf06520 | Large exoproteins involved in heme utilization or adhesion | COG3210 |
| DN1_orf06524 | Type II secretory pathway, component PulF | COG1459 |
| DN1_orf06526 | Type II secretory pathway, ATPase PulE/Tfp pilus assembly pathway, ATPase PilB | COG2804 |
| DN1_orf06528 | Type II secretory pathway, component PulD | COG1450 |
| DN1_orf06529 | Type II secretory pathway, component PulM | COG3149 |
| DN1_orf06531 | Type II secretory pathway, component PulK | COG3156 |
| DN1_orf06532 | Type II secretory pathway, pseudopilin PulG | COG2165 |
| DN1_orf06533 | Type II secretory pathway, pseudopilin PulG | COG2165 |
| DN1_orf06535 | Type II secretory pathway, pseudopilin PulG | COG2165 |
| DN1_orf06536 | Type II secretory pathway, component PulJ | COG4795 |
| DN1_orf06572 | Flp pilus assembly protein TadG | COG4961 |
| DN1_orf06579 | Flp pilus assembly protein TadC | COG2064 |
| DN1_orf06580 | Flp pilus assembly protein TadD, contains TPR repeats | COG5010 |
| DN1_orf06581 | Flp pilus assembly protein TadB | COG4965 |
| DN1_orf06583 | Flp pilus assembly protein, ATPase CpaE | COG4963 |
| DN1_orf06585 | Flp pilus assembly protein, ATPase CpaF | COG4962 |
| DN1_orf06586 | Flp pilus assembly protein, secretin CpaC | COG4964 |
| DN1_orf06587 | Flp pilus assembly protein CpaB | COG3745 |
| DN1_orf06588 | Flp pilus assembly protein, pilin Flp | COG3847 |
| DN1_orf06731 | Preprotein translocase subunit SecA (ATPase, RNA helicase) | COG0653 |
| DN1_orf06935 | Tfp pilus assembly protein, major pilin PilA | COG4969 |
| DN1_orf06936 | Type II secretory pathway, ATPase PulE/Tfp pilus assembly pathway, ATPase PilB | COG2804 |
| DN1_orf06937 | Type II secretory pathway, component PulF | COG1459 |
| DN1_orf06938 | Type II secretory pathway, prepilin signal peptidase PulO and related peptidases | COG1989 |
| DN1_orf06953 | Hemolysin activation/secretion protein | COG2831 |
| DN1_orf06954 | Large exoproteins involved in heme utilization or adhesion | COG3210 |
| DN1_orf06995 | Type II secretory pathway, ATPase PulE/Tfp pilus assembly pathway, ATPase PilB | COG2804 |
| DN1_orf06996 | Type II secretory pathway, component PulF | COG1459 |
| DN1_orf06999 | Tfp pilus assembly protein, ATPase PilU | COG5008 |
| DN1_orf07059 | Tfp pilus assembly protein FimT | COG4970 |
| DN1_orf07060 | Tfp pilus assembly protein PilV | COG4967 |
| DN1_orf07061 | Tfp pilus assembly protein PilW | COG4966 |
| DN1_orf07062 | Tfp pilus assembly protein PilX | COG4726 |
| DN1_orf07064 | Tfp pilus assembly protein, tip-associated adhesin PilY1 | COG3419 |
| DN1_orf07066 | Tfp pilus assembly protein PilE | COG4968 |
| DN1_orf07069 | Lipoprotein signal peptidase | COG0597 |
| DN1_orf07127 | Outer membrane protein | COG1538 |
| DN1_orf07173 | Hemolysin activation/secretion protein | COG2831 |
| DN1_orf07177 | Large exoproteins involved in heme utilization or adhesion | COG3210 |
| DN1_orf07211 | P pilus assembly protein, chaperone PapD | COG3121 |
| DN1_orf07213 | P pilus assembly protein, porin PapC | COG3188 |
| DN1_orf07300 | Preprotein translocase subunit SecD | COG0342 |
| DN1_orf07513 | Multiple antibiotic transporter | COG2095 |
| DN1_orf07680 | Outer membrane protein | COG1538 |
| DN1_orf07789 | Type II secretory pathway, component HofQ | COG4796 |
| DN1_orf07790 | Tfp pilus assembly protein PilP | COG3168 |
| DN1_orf07791 | Tfp pilus assembly protein PilO | COG3167 |
| DN1_orf07793 | Tfp pilus assembly protein PilN | COG3166 |
| DN1_orf07794 | Tfp pilus assembly protein, ATPase PilM | COG4972 |
| DN1_orf07827 | Sec-independent protein secretion pathway components | COG1826 |
| DN1_orf07829 | Sec-independent protein secretion pathway component TatC | COG0805 |
| DN1_orf07911 | Preprotein translocase subunit SecB | COG1952 |
| DN1_orf07954 | Outer membrane protein | COG1538 |
| DN1_orf08028 | Multiple antibiotic transporter | COG2095 |
| DN1_orf08036 | Type II secretory pathway, ATPase PulE/Tfp pilus assembly pathway, ATPase PilB | COG2804 |
| DN1_orf08037 | Membrane protein implicated in regulation of membrane protease activity | COG1585 |
| DN1_orf08142 | P pilus assembly protein, pilin FimA | COG3539 |
| DN1_orf08452 | Multiple antibiotic transporter | COG2095 |
| DN1_orf08565 | Preprotein translocase subunit YidC | COG0706 |
| plasmid_orf00407 | Flp pilus assembly protein, secretin CpaC | COG4964 |
| plasmid_orf00414 | Type II secretory pathway, ATPase PulE/Tfp pilus assembly pathway, ATPase PilB | COG2804 |
| plasmid_orf00417 | Type II secretory pathway, component PulF | COG1459 |
| plasmid_orf00446 | Tfp pilus assembly protein, pilus retraction ATPase PilT | COG2805 |
| plasmid_orf00610 | Type IV secretory pathway, VirB4 components | COG3451 |
