# Supplementary material for: Genome Sequence and Metabolic Analysis of a Fluoranthene-Degrading Strain Pseudomonas aeruginosa DN1
Source: Front Microbiol. 2018 Oct 31;9:2595. doi: 10.3389/fmicb.2018.02595 (PMC6220107; doi:10.3389/fmicb.2018.02595)
Supplement: Supplementary file 8 [file Table_8.DOCX]

**Table S8 | Unknown function**

| **Locus Tag** | **Gene Product Name** | **Function ID** |
| --- | --- | --- |
| DN1_orf00024 | Uncharacterized protein containing LysM domain | COG1652 |
| DN1_orf00051 | Uncharacterized conserved protein | COG3360 |
| DN1_orf00063 | Predicted membrane protein | COG3174 |
| DN1_orf00067 | Uncharacterized protein conserved in bacteria | COG4259 |
| DN1_orf00068 | Uncharacterized protein conserved in bacteria | COG4380 |
| DN1_orf00076 | Uncharacterized conserved protein, contains double-stranded beta-helix domain | COG1917 |
| DN1_orf00077 | Uncharacterized protein conserved in bacteria | COG3812 |
| DN1_orf00099 | Uncharacterized conserved protein | COG1262 |
| DN1_orf00109 | Uncharacterized protein conserved in bacteria | COG3523 |
| DN1_orf00110 | Uncharacterized protein conserved in bacteria | COG3913 |
| DN1_orf00113 | Uncharacterized protein conserved in bacteria | COG3522 |
| DN1_orf00114 | Uncharacterized protein conserved in bacteria | COG3521 |
| DN1_orf00120 | Uncharacterized protein conserved in bacteria | COG3515 |
| DN1_orf00121 | Uncharacterized protein conserved in bacteria | COG3516 |
| DN1_orf00124 | Uncharacterized protein conserved in bacteria | COG3517 |
| DN1_orf00126 | Hemolysin-coregulated protein (uncharacterized) | COG3157 |
| DN1_orf00129 | Uncharacterized protein conserved in bacteria | COG3518 |
| DN1_orf00131 | Uncharacterized protein conserved in bacteria | COG3519 |
| DN1_orf00133 | Uncharacterized protein conserved in bacteria | COG3520 |
| DN1_orf00137 | Uncharacterized protein conserved in bacteria | COG3501 |
| DN1_orf00142 | Uncharacterized conserved protein | COG5435 |
| DN1_orf00144 | Uncharacterized protein conserved in bacteria | COG3501 |
| DN1_orf00147 | Uncharacterized protein conserved in bacteria | COG5351 |
| DN1_orf00165 | Uncharacterized conserved protein | COG3346 |
| DN1_orf00208 | Uncharacterized conserved protein | COG2326 |
| DN1_orf00246 | Uncharacterized conserved protein | COG1739 |
| DN1_orf00334 | Uncharacterized homolog of gamma-carboxymuconolactone decarboxylase subunit | COG0599 |
| DN1_orf00366 | Predicted membrane protein | COG3619 |
| DN1_orf00397 | Uncharacterized conserved protein | COG2128 |
| DN1_orf00398 | Uncharacterized conserved protein, contains double-stranded beta-helix domain | COG1917 |
| DN1_orf00399 | Uncharacterized conserved protein | COG1359 |
| DN1_orf00407 | Predicted membrane protein | COG4539 |
| DN1_orf00419 | Uncharacterized small protein | COG5583 |
| DN1_orf00475 | Uncharacterized protein conserved in bacteria | COG3204 |
| DN1_orf00476 | Uncharacterized conserved protein | COG3111 |
| DN1_orf00486 | Uncharacterized protein conserved in bacteria | COG3204 |
| DN1_orf00491 | Uncharacterized conserved protein | COG3422 |
| DN1_orf00494 | Predicted integral membrane protein | COG5528 |
| DN1_orf00497 | Predicted integral membrane protein | COG5528 |
| DN1_orf00506 | Uncharacterized conserved protein | COG3332 |
| DN1_orf00560 | Uncharacterized small membrane protein | COG2363 |
| DN1_orf00581 | Predicted integral membrane protein | COG0762 |
| DN1_orf00589 | Predicted membrane protein | COG2314 |
| DN1_orf00619 | Uncharacterized protein conserved in bacteria | COG1385 |
| DN1_orf00625 | Uncharacterized conserved protein | COG2353 |
| DN1_orf00771 | Uncharacterized conserved protein | COG3391 |
| DN1_orf00791 | Uncharacterized protein conserved in bacteria | COG2258 |
| DN1_orf00801 | Uncharacterized conserved protein | COG1704 |
| DN1_orf00805 | Predicted membrane protein | COG3686 |
| DN1_orf00833 | Predicted membrane protein | COG3152 |
| DN1_orf00835 | Uncharacterized homolog of gamma-carboxymuconolactone decarboxylase subunit | COG0599 |
| DN1_orf00837 | Uncharacterized homolog of Blt101 | COG0401 |
| DN1_orf00862 | Uncharacterized conserved protein | COG1610 |
| DN1_orf00865 | Predicted membrane protein | COG0344 |
| DN1_orf00873 | Uncharacterized conserved protein | COG2719 |
| DN1_orf00874 | Uncharacterized conserved protein | COG2718 |
| DN1_orf00944 | Predicted membrane protein | COG3152 |
| DN1_orf00946 | Predicted membrane protein | COG1981 |
| DN1_orf00951 | Uncharacterized conserved protein | COG0316 |
| DN1_orf01041 | Uncharacterized iron-regulated membrane protein | COG3182 |
| DN1_orf01063 | Predicted membrane protein | COG2259 |
| DN1_orf01080 | Mu-like prophage protein gp16 | COG4382 |
| DN1_orf01090 | Mu-like prophage protein gp29 | COG4383 |
| DN1_orf01092 | Uncharacterized protein, homolog of phage Mu protein gp30 | COG2369 |
| DN1_orf01103 | Mu-like prophage protein gp36 | COG4387 |
| DN1_orf01125 | Uncharacterized iron-regulated membrane protein | COG3182 |
| DN1_orf01153 | Uncharacterized conserved protein | COG4744 |
| DN1_orf01155 | Uncharacterized conserved protein | COG1944 |
| DN1_orf01221 | Uncharacterized conserved protein | COG3287 |
| DN1_orf01236 | Uncharacterized low-complexity proteins | COG1357 |
| DN1_orf01255 | Uncharacterized protein conserved in bacteria | COG2315 |
| DN1_orf01256 | Uncharacterized protein conserved in bacteria | COG3554 |
| DN1_orf01294 | Uncharacterized protein conserved in bacteria | COG3204 |
| DN1_orf01310 | Uncharacterized protein conserved in bacteria | COG4519 |
| DN1_orf01321 | Predicted membrane protein | COG4392 |
| DN1_orf01333 | Uncharacterized conserved protein | COG0759 |
| DN1_orf01338 | Uncharacterized conserved protein | COG1359 |
| DN1_orf01363 | Uncharacterized conserved protein | COG3673 |
| DN1_orf01369 | Uncharacterized conserved protein | COG1434 |
| DN1_orf01426 | Predicted outer membrane protein | COG3652 |
| DN1_orf01439 | Uncharacterized conserved protein | COG4336 |
| DN1_orf01469 | Predicted outer membrane protein | COG3652 |
| DN1_orf01494 | Uncharacterized membrane protein | COG1285 |
| DN1_orf01496 | Uncharacterized conserved protein | COG1273 |
| DN1_orf01505 | Predicted integral membrane protein | COG0392 |
| DN1_orf01508 | Uncharacterized conserved protein | COG1801 |
| DN1_orf01544 | Uncharacterized conserved protein | COG2170 |
| DN1_orf01546 | Predicted small integral membrane protein | COG5478 |
| DN1_orf01560 | Uncharacterized conserved protein | COG1359 |
| DN1_orf01575 | Uncharacterized protein conserved in bacteria | COG3333 |
| DN1_orf01576 | Uncharacterized protein conserved in bacteria | COG3181 |
| DN1_orf01586 | Uncharacterized protein conserved in bacteria | COG3802 |
| DN1_orf01590 | Uncharacterized paraquat-inducible protein A | COG2995 |
| DN1_orf01602 | Predicted membrane protein | COG3162 |
| DN1_orf01627 | Uncharacterized protein conserved in bacteria | COG2258 |
| DN1_orf01628 | Uncharacterized protein conserved in bacteria | COG4764 |
| DN1_orf01723 | Uncharacterized protein conserved in bacteria | COG3536 |
| DN1_orf01732 | Uncharacterized conserved protein | COG2606 |
| DN1_orf01783 | Uncharacterized conserved protein | COG2128 |
| DN1_orf01823 | Uncharacterized protein conserved in bacteria | COG3515 |
| DN1_orf01825 | Uncharacterized protein conserved in bacteria | COG3523 |
| DN1_orf01826 | Uncharacterized protein conserved in bacteria | COG3455 |
| DN1_orf01827 | Uncharacterized protein conserved in bacteria | COG3522 |
| DN1_orf01829 | Uncharacterized protein conserved in bacteria | COG3516 |
| DN1_orf01831 | Uncharacterized protein conserved in bacteria | COG3517 |
| DN1_orf01834 | Hemolysin-coregulated protein (uncharacterized) | COG3157 |
| DN1_orf01835 | Uncharacterized protein conserved in bacteria | COG3518 |
| DN1_orf01836 | Uncharacterized protein conserved in bacteria | COG3519 |
| DN1_orf01837 | Uncharacterized protein conserved in bacteria | COG3520 |
| DN1_orf01841 | Uncharacterized protein conserved in bacteria | COG3501 |
| DN1_orf01844 | Uncharacterized protein conserved in bacteria | COG2849 |
| DN1_orf01874 | Uncharacterized conserved protein | COG1262 |
| DN1_orf01891 | Uncharacterized iron-regulated membrane protein | COG3182 |
| DN1_orf01901 | Uncharacterized protein conserved in bacteria | COG3251 |
| DN1_orf01905 | Predicted membrane protein | COG4244 |
| DN1_orf01930 | Uncharacterized conserved protein | COG2326 |
| DN1_orf01935 | Predicted membrane protein | COG1289 |
| DN1_orf01994 | Uncharacterized iron-regulated membrane protein | COG3182 |
| DN1_orf02041 | Uncharacterized protein conserved in bacteria | COG2318 |
| DN1_orf02050 | Uncharacterized protein conserved in bacteria | COG4859 |
| DN1_orf02118 | Uncharacterized protein conserved in bacteria | COG2911 |
| DN1_orf02142 | Uncharacterized membrane protein | COG1285 |
| DN1_orf02179 | Phage-related protein, tail component | COG4733 |
| DN1_orf02184 | Phage-related protein | COG4672 |
| DN1_orf02186 | Phage-related protein | COG4718 |
| DN1_orf02189 | Phage-related minor tail protein | COG5281 |
| DN1_orf02209 | Uncharacterized conserved protein | COG1598 |
| DN1_orf02286 | Phage-related protein, tail component | COG4733 |
| DN1_orf02287 | Phage-related protein, tail component | COG4723 |
| DN1_orf02289 | Phage-related protein | COG4672 |
| DN1_orf02292 | Phage-related minor tail protein | COG5281 |
| DN1_orf02302 | Uncharacterized protein conserved in bacteria | COG4834 |
| DN1_orf02305 | Uncharacterized protein conserved in bacteria | COG3566 |
| DN1_orf02306 | Uncharacterized protein conserved in bacteria | COG3567 |
| DN1_orf02367 | Uncharacterized conserved protein | COG2127 |
| DN1_orf02381 | Uncharacterized conserved protein | COG2850 |
| DN1_orf02367 | Uncharacterized conserved protein | COG2127 |
| DN1_orf02381 | Uncharacterized conserved protein | COG2850 |
| DN1_orf02424 | Predicted membrane protein | COG3212 |
| DN1_orf02425 | Predicted membrane protein | COG3212 |
| DN1_orf02465 | Uncharacterized protein conserved in bacteria | COG3501 |
| DN1_orf02482 | Uncharacterized protein conserved in bacteria | COG4317 |
| DN1_orf02492 | Uncharacterized protein conserved in bacteria | COG3825 |
| DN1_orf02493 | Uncharacterized conserved protein | COG3791 |
| DN1_orf02513 | Uncharacterized protein conserved in bacteria | COG3865 |
| DN1_orf02514 | Uncharacterized conserved protein | COG3791 |
| DN1_orf02566 | Predicted membrane protein | COG1295 |
| DN1_orf02568 | Predicted membrane protein | COG1238 |
| DN1_orf02570 | Uncharacterized conserved protein | COG4575 |
| DN1_orf02577 | Predicted membrane protein | COG4125 |
| DN1_orf02695 | Uncharacterized conserved protein | COG3384 |
| DN1_orf02706 | Uncharacterized protein conserved in bacteria | COG3214 |
| DN1_orf02708 | Uncharacterized conserved small protein | COG5457 |
| DN1_orf02717 | Uncharacterized protein conserved in bacteria | COG4394 |
| DN1_orf02719 | Uncharacterized protein conserved in bacteria | COG1376 |
| DN1_orf02732 | Predicted membrane protein | COG2259 |
| DN1_orf02740 | Uncharacterized protein conserved in bacteria | COG3915 |
| DN1_orf02745 | Uncharacterized conserved protein | COG5607 |
| DN1_orf02754 | Uncharacterized protein conserved in bacteria | COG3803 |
| DN1_orf02756 | Predicted membrane protein | COG4125 |
| DN1_orf02805 | Predicted membrane protein | COG1971 |
| DN1_orf02869 | Uncharacterized paraquat-inducible protein B | COG3007 |
| DN1_orf02875 | Uncharacterized protein conserved in bacteria | COG3816 |
| DN1_orf02907 | Uncharacterized conserved protein | COG2835 |
| DN1_orf02914 | Uncharacterized protein conserved in bacteria | COG3216 |
| DN1_orf02925 | Uncharacterized protein conserved in bacteria | COG2991 |
| DN1_orf02953 | Uncharacterized protein conserved in bacteria | COG4728 |
| DN1_orf02965 | Uncharacterized protein conserved in bacteria | COG3755 |
| DN1_orf02989 | Uncharacterized conserved protein | COG4575 |
| DN1_orf02990 | Predicted membrane protein | COG5393 |
| DN1_orf02997 | Uncharacterized protein conserved in bacteria | COG3171 |
| DN1_orf03010 | Predicted membrane protein | COG4267 |
| DN1_orf03024 | Uncharacterized conserved protein | COG3868 |
| DN1_orf03052 | Uncharacterized protein conserved in bacteria | COG3139 |
| DN1_orf03057 | Uncharacterized protein conserved in bacteria | COG3782 |
| DN1_orf03089 | Uncharacterized protein conserved in bacteria | COG3147 |
| DN1_orf03193 | Uncharacterized conserved protein | COG1354 |
| DN1_orf03197 | Uncharacterized protein conserved in bacteria | COG2350 |
| DN1_orf03218 | Predicted membrane protein | COG4323 |
| DN1_orf03221 | Uncharacterized protein conserved in bacteria | COG2908 |
| DN1_orf03241 | Uncharacterized protein conserved in bacteria | COG3146 |
| DN1_orf03249 | Predicted membrane protein | COG3162 |
| DN1_orf03251 | Predicted membrane protein | COG3205 |
| DN1_orf03306 | Uncharacterized conserved protein | COG1742 |
| DN1_orf03314 | Uncharacterized protein conserved in bacteria | COG3219 |
| DN1_orf03315 | Predicted membrane protein | COG2259 |
| DN1_orf03316 | Uncharacterized protein conserved in bacteria | COG3220 |
| DN1_orf03317 | Uncharacterized low-complexity protein | COG3767 |
| DN1_orf03330 | Uncharacterized protein conserved in bacteria | COG3501 |
| DN1_orf03352 | Predicted membrane protein | COG1289 |
| DN1_orf03378 | Uncharacterized protein conserved in bacteria | COG3238 |
| DN1_orf03428 | Uncharacterized protein conserved in bacteria | COG3222 |
| DN1_orf03475 | Uncharacterized conserved protein | COG1720 |
| DN1_orf03477 | Uncharacterized conserved protein | COG1359 |
| DN1_orf03498 | Predicted membrane protein | COG3223 |
| DN1_orf03515 | Uncharacterized protein conserved in bacteria | COG3141 |
| DN1_orf03525 | Uncharacterized conserved protein | COG2912 |
| DN1_orf03545 | Uncharacterized protein conserved in bacteria | COG3224 |
| DN1_orf03549 | Uncharacterized protein conserved in bacteria | COG3492 |
| DN1_orf03566 | Uncharacterized protein conserved in bacteria | COG3132 |
| DN1_orf03572 | Uncharacterized conserved protein | COG2326 |
| DN1_orf03574 | Uncharacterized conserved protein | COG4121 |
| DN1_orf03590 | Uncharacterized protein conserved in bacteria | COG3089 |
| DN1_orf03629 | Uncharacterized protein conserved in bacteria | COG3501 |
| DN1_orf03672 | Uncharacterized protein with a C-terminal OMP (outer membrane protein) domain | COG4625 |
| DN1_orf03678 | Uncharacterized protein conserved in bacteria | COG3022 |
| DN1_orf03727 | Uncharacterized conserved protein | COG1359 |
| DN1_orf03749 | Uncharacterized conserved protein | COG2606 |
| DN1_orf03786 | Uncharacterized conserved protein | COG3148 |
| DN1_orf03793 | Uncharacterized protein conserved in bacteria | COG3530 |
| DN1_orf03815 | Uncharacterized conserved protein | COG0585 |
| DN1_orf03880 | Uncharacterized protein conserved in bacteria | COG3126 |
| DN1_orf03891 | Uncharacterized protein conserved in bacteria | COG3921 |
| DN1_orf03893 | Uncharacterized conserved protein | COG1801 |
| DN1_orf03909 | Uncharacterized conserved protein | COG2013 |
| DN1_orf03911 | Uncharacterized protein conserved in bacteria | COG3930 |
| DN1_orf03913 | Uncharacterized protein conserved in bacteria | COG3226 |
| DN1_orf03933 | Uncharacterized protein conserved in bacteria | COG2979 |
| DN1_orf03953 | Uncharacterized protein conserved in bacteria | COG4681 |
| DN1_orf03959 | Uncharacterized protein conserved in bacteria | COG2268 |
| DN1_orf03962 | Uncharacterized protein conserved in bacteria | COG3789 |
| DN1_orf03994 | Uncharacterized protein conserved in bacteria | COG3034 |
| DN1_orf04004 | Uncharacterized protein conserved in bacteria | COG3323 |
| DN1_orf04010 | Uncharacterized protein conserved in bacteria | COG3644 |
| DN1_orf04059 | Uncharacterized protein conserved in bacteria | COG2847 |
| DN1_orf04064 | Uncharacterized iron-regulated membrane protein | COG3182 |
| DN1_orf04073 | Uncharacterized protein conserved in bacteria | COG3753 |
| DN1_orf04079 | FOG: WD40-like repeat | COG1520 |
| DN1_orf04080 | Uncharacterized protein conserved in bacteria | COG2976 |
| DN1_orf04085 | Uncharacterized protein conserved in bacteria | COG1426 |
| DN1_orf04090 | Uncharacterized protein conserved in bacteria | COG2975 |
| DN1_orf04095 | Uncharacterized conserved protein | COG0316 |
| DN1_orf04104 | Predicted outer membrane lipoprotein | COG3134 |
| DN1_orf04116 | Predicted membrane protein/domain | COG1714 |
| DN1_orf04152 | Uncharacterized membrane protein | COG1814 |
| DN1_orf04174 | Uncharacterized protein conserved in bacteria | COG2964 |
| DN1_orf04196 | Uncharacterized conserved protein | COG4273 |
| DN1_orf04220 | Predicted membrane protein | COG1289 |
| DN1_orf04234 | Uncharacterized conserved protein | COG4104 |
| DN1_orf04254 | Uncharacterized conserved protein | COG5607 |
| DN1_orf04287 | Predicted membrane protein | COG1297 |
| DN1_orf04312 | Uncharacterized conserved protein | COG1432 |
| DN1_orf04313 | Uncharacterized protein conserved in bacteria | COG3416 |
| DN1_orf04351 | Uncharacterized protein conserved in bacteria | COG4517 |
| DN1_orf04359 | Uncharacterized conserved protein | COG1434 |
| DN1_orf04367 | Uncharacterized protein conserved in bacteria | COG3036 |
| DN1_orf04374 | Uncharacterized conserved protein | COG2921 |
| DN1_orf04381 | Uncharacterized conserved protein | COG1576 |
| DN1_orf04383 | Uncharacterized homolog of plant Iojap protein | COG0799 |
| DN1_orf04391 | Uncharacterized membrane-associated protein | COG0586 |
| DN1_orf04398 | Uncharacterized protein conserved in bacteria | COG4642 |
| DN1_orf04421 | Uncharacterized membrane-associated protein | COG0586 |
| DN1_orf04422 | Uncharacterized protein conserved in bacteria | COG3228 |
| DN1_orf04428 | Uncharacterized protein conserved in bacteria | COG4254 |
| DN1_orf04461 | Uncharacterized protein conserved in bacteria | COG3126 |
| DN1_orf04470 | Uncharacterized protein conserved in bacteria | COG3495 |
| DN1_orf04504 | Uncharacterized protein conserved in bacteria | COG3553 |
| DN1_orf04540 | Predicted periplasmic protein | COG3904 |
| DN1_orf04547 | Uncharacterized conserved protein | COG3384 |
| DN1_orf04553 | Uncharacterized protein conserved in bacteria | COG3749 |
| DN1_orf04586 | Uncharacterized conserved protein | COG3199 |
| DN1_orf04621 | Uncharacterized conserved protein | COG3791 |
| DN1_orf04628 | Uncharacterized conserved protein | COG5569 |
| DN1_orf04636 | Uncharacterized conserved protein | COG3382 |
| DN1_orf04684 | Mu-like prophage protein gp36 | COG4387 |
| DN1_orf04695 | Uncharacterized protein, homolog of phage Mu protein gp30 | COG2369 |
| DN1_orf04697 | Mu-like prophage protein gp29 | COG4383 |
| DN1_orf04707 | Mu-like prophage protein gp16 | COG4382 |
| DN1_orf04799 | Uncharacterized protein conserved in bacteria | COG2354 |
| DN1_orf04810 | Uncharacterized protein conserved in bacteria | COG3813 |
| DN1_orf04812 | Uncharacterized conserved protein | COG0316 |
| DN1_orf04823 | Uncharacterized protein conserved in bacteria | COG3171 |
| DN1_orf04825 | Uncharacterized conserved protein | COG3760 |
| DN1_orf04832 | Uncharacterized protein conserved in bacteria | COG3749 |
| DN1_orf04897 | Uncharacterized protein conserved in bacteria | COG2908 |
| DN1_orf04931 | Uncharacterized protein conserved in bacteria | COG1806 |
| DN1_orf04983 | Uncharacterized protein conserved in bacteria | COG2307 |
| DN1_orf04984 | Uncharacterized conserved protein | COG2308 |
| DN1_orf05016 | Uncharacterized conserved protein | COG5613 |
| DN1_orf05048 | Uncharacterized conserved protein | COG3391 |
| DN1_orf05054 | Uncharacterized conserved protein, contains double-stranded beta-helix domain | COG1791 |
| DN1_orf05066 | Uncharacterized protein conserved in bacteria | COG2840 |
| DN1_orf05073 | Uncharacterized protein conserved in bacteria | COG3523 |
| DN1_orf05074 | Uncharacterized protein conserved in bacteria | COG3455 |
| DN1_orf05076 | Uncharacterized protein conserved in bacteria | COG3522 |
| DN1_orf05078 | Uncharacterized protein conserved in bacteria | COG3521 |
| DN1_orf05086 | Uncharacterized protein conserved in bacteria | COG3520 |
| DN1_orf05087 | Uncharacterized protein conserved in bacteria | COG3519 |
| DN1_orf05088 | Uncharacterized protein conserved in bacteria | COG3518 |
| DN1_orf05090 | Uncharacterized protein conserved in bacteria | COG3517 |
| DN1_orf05092 | Uncharacterized protein conserved in bacteria | COG3516 |
| DN1_orf05093 | Uncharacterized protein conserved in bacteria | COG3515 |
| DN1_orf05111 | Uncharacterized conserved protein | COG3791 |
| DN1_orf05190 | Predicted membrane protein | COG3821 |
| DN1_orf05210 | Uncharacterized protein conserved in bacteria | COG3169 |
| DN1_orf05213 | Uncharacterized protein conserved in bacteria | COG3123 |
| DN1_orf05214 | Uncharacterized protein conserved in bacteria | COG3738 |
| DN1_orf05216 | Uncharacterized conserved protein | COG3199 |
| DN1_orf05254 | Uncharacterized protein conserved in bacteria | COG3198 |
| DN1_orf05258 | Uncharacterized conserved protein | COG2836 |
| DN1_orf05270 | Uncharacterized protein conserved in bacteria | COG3196 |
| DN1_orf05274 | Uncharacterized protein conserved in bacteria | COG0718 |
| DN1_orf05277 | Uncharacterized conserved protein | COG4748 |
| DN1_orf05302 | Uncharacterized protein conserved in bacteria | COG3195 |
| DN1_orf05305 | Predicted membrane protein | COG3748 |
| DN1_orf05307 | Hemolysin-coregulated protein (uncharacterized) | COG3157 |
| DN1_orf05309 | Uncharacterized protein conserved in bacteria | COG4253 |
| DN1_orf05312 | Uncharacterized conserved protein | COG4104 |
| DN1_orf05331 | Uncharacterized protein conserved in bacteria | COG4339 |
| DN1_orf05366 | Uncharacterized homolog of the cytoplasmic domain of flagellar protein FhlB | COG2257 |
| DN1_orf05369 | Uncharacterized conserved protein | COG1496 |
| DN1_orf05413 | Uncharacterized protein conserved in bacteria | COG2832 |
| DN1_orf05422 | Predicted periplasmic protein | COG3672 |
| DN1_orf05436 | Uncharacterized conserved protein | COG3148 |
| DN1_orf05490 | Uncharacterized protein conserved in bacteria | COG3501 |
| DN1_orf05526 | Uncharacterized protein conserved in bacteria | COG3865 |
| DN1_orf05528 | Uncharacterized conserved protein | COG2606 |
| DN1_orf05531 | Uncharacterized protein conserved in bacteria | COG3795 |
| DN1_orf05532 | Uncharacterized protein conserved in bacteria | COG2764 |
| DN1_orf05536 | Uncharacterized protein conserved in bacteria | COG4312 |
| DN1_orf05537 | Uncharacterized protein conserved in bacteria | COG3795 |
| DN1_orf05574 | Ketosteroid isomerase homolog | COG4319 |
| DN1_orf05577 | Uncharacterized conserved protein | COG4575 |
| DN1_orf05594 | Uncharacterized protein conserved in bacteria | COG4460 |
| DN1_orf05615 | Uncharacterized conserved protein | COG2983 |
| DN1_orf05616 | Uncharacterized protein conserved in bacteria | COG1937 |
| DN1_orf05620 | Uncharacterized protein conserved in bacteria | COG3100 |
| DN1_orf05662 | Predicted membrane protein | COG1289 |
| DN1_orf05677 | Uncharacterized conserved protein | COG1786 |
| DN1_orf05724 | Predicted membrane protein | COG1289 |
| DN1_orf05787 | Uncharacterized protein conserved in bacteria | COG3091 |
| DN1_orf05846 | Uncharacterized protein conserved in bacteria | COG4893 |
| DN1_orf05864 | Uncharacterized protein with SCP/PR1 domains | COG2340 |
| DN1_orf05872 | Uncharacterized protein conserved in bacteria | COG3011 |
| DN1_orf05897 | Uncharacterized protein conserved in bacteria | COG2996 |
| DN1_orf05901 | Uncharacterized protein conserved in bacteria, putative lipoprotein | COG4461 |
| DN1_orf05905 | Uncharacterized conserved protein | COG4628 |
| DN1_orf05963 | Uncharacterized conserved small protein | COG5626 |
| DN1_orf05975 | Uncharacterized conserved protein | COG3189 |
| DN1_orf05976 | Predicted membrane protein | COG3650 |
| DN1_orf06003 | Predicted integral membrane protein | COG5615 |
| DN1_orf06006 | Predicted membrane protein | COG3776 |
| DN1_orf06009 | Uncharacterized protein conserved in bacteria | COG3012 |
| DN1_orf06011 | Predicted membrane protein | COG2860 |
| DN1_orf06013 | Conserved secreted protein | COG5608 |
| DN1_orf06020 | Uncharacterized protein conserved in bacteria | COG1322 |
| DN1_orf06118 | Uncharacterized protein conserved in bacteria | COG1729 |
| DN1_orf06129 | Uncharacterized conserved protein | COG0217 |
| DN1_orf06145 | Predicted membrane protein | COG1295 |
| DN1_orf06150 | Predicted membrane protein | COG3308 |
| DN1_orf06152 | Uncharacterized protein conserved in bacteria | COG3249 |
| DN1_orf06163 | Uncharacterized protein conserved in bacteria | COG3122 |
| DN1_orf06188 | Uncharacterized conserved protein | COG2898 |
| DN1_orf06199 | Uncharacterized protein conserved in bacteria | COG4807 |
| DN1_orf06251 | Uncharacterized conserved protein | COG3777 |
| DN1_orf06255 | Predicted membrane protein | COG1289 |
| DN1_orf06274 | Uncharacterized protein conserved in bacteria | COG4317 |
| DN1_orf06287 | Uncharacterized protein conserved in bacteria | COG3184 |
| DN1_orf06293 | Uncharacterized protein conserved in bacteria | COG3397 |
| DN1_orf06309 | Uncharacterized protein conserved in bacteria | COG1307 |
| DN1_orf06332 | Predicted membrane protein | COG2314 |
| DN1_orf06333 | Uncharacterized conserved protein | COG4104 |
| DN1_orf06360 | Uncharacterized conserved protein | COG3832 |
| DN1_orf06363 | Predicted membrane protein | COG4270 |
| DN1_orf06366 | Uncharacterized protein conserved in bacteria | COG2764 |
| DN1_orf06369 | Uncharacterized iron-regulated membrane protein | COG3182 |
| DN1_orf06382 | Uncharacterized protein conserved in bacteria | COG2828 |
| DN1_orf06414 | Predicted secreted protein | COG5513 |
| DN1_orf06439 | Uncharacterized conserved protein | COG2938 |
| DN1_orf06447 | Uncharacterized protein conserved in bacteria | COG3181 |
| DN1_orf06450 | Uncharacterized protein conserved in bacteria | COG3333 |
| DN1_orf06469 | Predicted membrane protein | COG3776 |
| DN1_orf06477 | Uncharacterized protein conserved in bacteria | COG1376 |
| DN1_orf06482 | Uncharacterized protein conserved in bacteria | COG3811 |
| DN1_orf06485 | Uncharacterized conserved protein | COG1359 |
| DN1_orf06575 | Predicted membrane protein | COG4655 |
| DN1_orf06601 | Predicted integral membrane protein | COG0392 |
| DN1_orf06607 | Predicted membrane protein/domain | COG1714 |
| DN1_orf06608 | Uncharacterized membrane protein | COG1300 |
| DN1_orf06663 | Uncharacterized protein conserved in bacteria | COG3124 |
| DN1_orf06675 | Uncharacterized conserved protein | COG3268 |
| DN1_orf06678 | Uncharacterized protein conserved in bacteria | COG3602 |
| DN1_orf06685 | Uncharacterized conserved protein | COG1704 |
| DN1_orf06690 | Uncharacterized protein conserved in bacteria | COG3490 |
| DN1_orf06705 | Uncharacterized protein conserved in bacteria | COG4318 |
| DN1_orf06718 | Uncharacterized protein conserved in bacteria | COG1666 |
| DN1_orf06726 | Uncharacterized conserved protein | COG2096 |
| DN1_orf06782 | Uncharacterized protein conserved in bacteria | COG3105 |
| DN1_orf06788 | Uncharacterized conserved protein | COG0327 |
| DN1_orf06804 | Uncharacterized protein conserved in bacteria | COG3117 |
| DN1_orf06805 | Uncharacterized protein conserved in bacteria | COG1934 |
| DN1_orf06824 | Uncharacterized conserved protein | COG5484 |
| DN1_orf06851 | Uncharacterized conserved protein | COG1598 |
| DN1_orf06860 | Uncharacterized protein conserved in bacteria | COG3028 |
| DN1_orf06865 | Predicted membrane protein | COG3164 |
| DN1_orf06877 | Uncharacterized homolog of gamma-carboxymuconolactone decarboxylase subunit | COG0599 |
| DN1_orf06878 | Uncharacterized protein conserved in bacteria | COG4676 |
| DN1_orf06880 | Predicted secreted protein | COG5445 |
| DN1_orf06884 | Uncharacterized protein conserved in bacteria | COG3234 |
| DN1_orf06885 | Uncharacterized protein conserved in bacteria | COG4685 |
| DN1_orf06887 | Uncharacterized protein conserved in bacteria | COG4676 |
| DN1_orf06891 | Predicted periplasmic/secreted protein | COG3471 |
| DN1_orf06921 | Uncharacterized iron-regulated protein | COG3128 |
| DN1_orf06941 | Uncharacterized protein conserved in bacteria | COG3024 |
| DN1_orf06944 | Predicted membrane protein | COG3235 |
| DN1_orf06948 | Uncharacterized protein conserved in bacteria | COG2258 |
| DN1_orf06981 | Uncharacterized phage-encoded protein | COG3646 |
| DN1_orf07002 | Uncharacterized conserved protein | COG5464 |
| DN1_orf07049 | Uncharacterized conserved protein | COG1496 |
| DN1_orf07077 | Uncharacterized protein conserved in bacteria | COG3045 |
| DN1_orf07091 | Predicted membrane protein | COG2862 |
| DN1_orf07099 | Uncharacterized protein conserved in bacteria | COG3236 |
| DN1_orf07105 | Uncharacterized conserved protein | COG1690 |
| DN1_orf07144 | Uncharacterized small protein | COG2879 |
| DN1_orf07153 | Uncharacterized protein conserved in bacteria | COG2841 |
| DN1_orf07183 | Predicted membrane protein | COG2119 |
| DN1_orf07190 | Uncharacterized conserved protein | COG2135 |
| DN1_orf07191 | Uncharacterized membrane protein | COG1285 |
| DN1_orf07200 | Uncharacterized protein conserved in bacteria | COG2764 |
| DN1_orf07208 | Uncharacterized secreted protein | COG5430 |
| DN1_orf07209 | Uncharacterized secreted protein | COG5430 |
| DN1_orf07210 | Uncharacterized secreted protein | COG5430 |
| DN1_orf07214 | Uncharacterized secreted protein | COG5430 |
| DN1_orf07220 | Uncharacterized conserved protein | COG3272 |
| DN1_orf07264 | Uncharacterized paraquat-inducible protein A | COG2995 |
| DN1_orf07265 | Uncharacterized paraquat-inducible protein A | COG2995 |
| DN1_orf07266 | Predicted membrane protein | COG2717 |
| DN1_orf07276 | Uncharacterized protein conserved in bacteria | COG3098 |
| DN1_orf07279 | Membrane carboxypeptidase (penicillin-binding protein) | COG0744 |
| DN1_orf07281 | Uncharacterized protein conserved in bacteria | COG2187 |
| DN1_orf07282 | Uncharacterized low-complexity proteins | COG1357 |
| DN1_orf07285 | Uncharacterized iron-regulated protein | COG3016 |
| DN1_orf07325 | Uncharacterized protein conserved in bacteria | COG3662 |
| DN1_orf07331 | Uncharacterized protein conserved in bacteria | COG3237 |
| DN1_orf07341 | Uncharacterized protein conserved in bacteria | COG0779 |
| DN1_orf07369 | Uncharacterized protein conserved in bacteria | COG2914 |
| DN1_orf07389 | Uncharacterized protein conserved in bacteria | COG3575 |
| DN1_orf07411 | Uncharacterized conserved protein | COG2320 |
| DN1_orf07444 | Uncharacterized protein conserved in bacteria | COG3238 |
| DN1_orf07453 | Predicted membrane protein | COG2246 |
| DN1_orf07466 | Predicted membrane protein | COG3556 |
| DN1_orf07475 | Uncharacterized protein conserved in bacteria | COG4408 |
| DN1_orf07480 | Predicted membrane protein | COG3503 |
| DN1_orf07487 | Uncharacterized protein conserved in bacteria | COG5345 |
| DN1_orf07537 | Uncharacterized protein conserved in bacteria | COG2258 |
| DN1_orf07607 | Uncharacterized conserved protein | COG2308 |
| DN1_orf07629 | Uncharacterized protein conserved in bacteria | COG3242 |
| DN1_orf07664 | Uncharacterized protein conserved in bacteria | COG3009 |
| DN1_orf07675 | Uncharacterized protein conserved in bacteria | COG3151 |
| DN1_orf07764 | Uncharacterized conserved protein | COG0397 |
| DN1_orf07784 | Uncharacterized protein conserved in bacteria | COG3266 |
| DN1_orf07811 | Uncharacterized protein conserved in bacteria | COG3536 |
| DN1_orf07822 | Uncharacterized protein conserved in bacteria | COG3165 |
| DN1_orf07831 | Uncharacterized protein conserved in bacteria | COG1385 |
| DN1_orf07854 | Uncharacterized protein conserved in bacteria | COG3501 |
| DN1_orf07878 | Uncharacterized protein conserved in bacteria | COG3758 |
| DN1_orf07881 | Uncharacterized protein conserved in bacteria | COG3803 |
| DN1_orf07890 | Predicted membrane protein | COG5373 |
| DN1_orf07921 | Uncharacterized protein conserved in bacteria | COG2861 |
| DN1_orf07945 | Uncharacterized conserved protein | COG2928 |
| DN1_orf07988 | Uncharacterized protein containing LysM domain | COG1652 |
| DN1_orf08033 | Uncharacterized conserved protein | COG3025 |
| DN1_orf08057 | Uncharacterized protein conserved in bacteria | COG3079 |
| DN1_orf08059 | Uncharacterized protein conserved in bacteria | COG3027 |
| DN1_orf08061 | Uncharacterized conserved protein | COG2947 |
| DN1_orf08085 | Uncharacterized membrane-associated protein | COG0586 |
| DN1_orf08088 | Uncharacterized protein conserved in bacteria | COG1671 |
| DN1_orf08098 | Uncharacterized conserved protein | COG5589 |
| DN1_orf08117 | Uncharacterized protein conserved in bacteria | COG3501 |
| DN1_orf08119 | Hemolysin-coregulated protein (uncharacterized) | COG3157 |
| DN1_orf08126 | Uncharacterized conserved protein | COG4278 |
| DN1_orf08134 | Uncharacterized protein conserved in bacteria | COG3159 |
| DN1_orf08145 | Uncharacterized conserved protein | COG0432 |
| DN1_orf08151 | Uncharacterized protein conserved in bacteria | COG2960 |
| DN1_orf08178 | Uncharacterized protein conserved in bacteria | COG3784 |
| DN1_orf08219 | Uncharacterized protein conserved in bacteria | COG3296 |
| DN1_orf08221 | Uncharacterized stress-induced protein | COG1561 |
| DN1_orf08237 | Uncharacterized conserved protein | COG2606 |
| DN1_orf08254 | Uncharacterized protein conserved in bacteria | COG4315 |
| DN1_orf08291 | Predicted membrane protein | COG2855 |
| DN1_orf08296 | Uncharacterized conserved protein | COG3246 |
| DN1_orf08301 | Uncharacterized conserved protein | COG3342 |
| DN1_orf08306 | Uncharacterized protein conserved in bacteria | COG3558 |
| DN1_orf08325 | Uncharacterized conserved protein | COG3247 |
| DN1_orf08349 | Uncharacterized protein conserved in bacteria | COG3495 |
| DN1_orf08388 | Predicted membrane protein | COG2510 |
| DN1_orf08391 | Uncharacterized conserved small protein | COG5457 |
| DN1_orf08412 | Uncharacterized conserved small protein | COG5457 |
| DN1_orf08420 | Uncharacterized conserved protein | COG3791 |
| DN1_orf08430 | Uncharacterized conserved protein | COG1690 |
| DN1_orf08492 | Predicted membrane protein | COG1238 |
| DN1_orf08507 | Uncharacterized protein conserved in bacteria | COG4395 |
| DN1_orf08522 | Uncharacterized conserved protein | COG1469 |
| DN1_orf08529 | Uncharacterized conserved protein | COG4729 |
| DN1_orf01473 | Predicted membrane protein | COG2323 |
| plasmid_orf00269 | Uncharacterized protein conserved in bacteria | COG3141 |
| plasmid_orf00277 | Predicted periplasmic protein | COG3904 |
| plasmid_orf00310 | Uncharacterized protein conserved in bacteria | COG3089 |
| plasmid_orf00375 | Uncharacterized protein conserved in bacteria | COG4643 |
| plasmid_orf00554 | Uncharacterized protein with a C-terminal OMP (outer membrane protein) domain | COG4625 |
