# Supplementary material for: Genome Sequence and Metabolic Analysis of a Fluoranthene-Degrading Strain Pseudomonas aeruginosa DN1
Source: Front Microbiol. 2018 Oct 31;9:2595. doi: 10.3389/fmicb.2018.02595 (PMC6220107; doi:10.3389/fmicb.2018.02595)
Supplement: Supplementary file 9 [file Table_9.DOCX]

**Table S9 |** Genomic comparisons with closely related bacteria

| Organism | Accession number | Isolation source | Degration | Reference |
| --- | --- | --- | --- | --- |
| DN1 | CP017099 | oily-sludge-contaminated soil | Fluoranthene | LU WEI, et al.2015 |
| PAO1 | AE004091.2 | human infected wound | N/A | [Stover CK, et al.2015](https://www.ncbi.nlm.nih.gov/pubmed/?term=Stover%20CK%5BAuthor%5D&cauthor=true&cauthor_uid=10984043) |
| KF702 | ZN-BBQK00000000.1 | Biphenyl-Contaminated Soil | Polychlorinated Biphenyl | Watanabe, T, et al.2015 |
| N002 | ZN-ALBV00000000.2 | crude oil contaminated soil | crude oil | Das, D, et al.2015 |
| DSM50071 | CP012001.1 | oil contaminated soil | crude oil | Nakano, K., et al.2015 |

N/A：Barely reported
