# Supplementary material for: Genome Sequence and Metabolic Analysis of a Fluoranthene-Degrading Strain Pseudomonas aeruginosa DN1
Source: Front Microbiol. 2018 Oct 31;9:2595. doi: 10.3389/fmicb.2018.02595 (PMC6220107; doi:10.3389/fmicb.2018.02595)
Supplement: Supplementary file 10 [file Table_10.DOCX]

**Table S10 |**Comparing of gene contents across genomes

| COG categories | DN1 | PAO1 | KF702 | N002 | DSM50071 |
| --- | --- | --- | --- | --- | --- |
| Amino acid transport and metabolism | 549 | 488 | 501 | 479 | 491 |
| Carbohydrate transport and metabolism | 226 | 217 | 228 | 214 | 219 |
| Cell cycle control, cell division, chromosome partitioning | 33 | 41 | 44 | 41 | 42 |
| Cell motility | 149 | 140 | 151 | 132 | 142 |
| Cell wall/membrane/envelope biogenesis | 252 | 270 | 268 | 248 | 270 |
| Chromatin structure and dynamics | 3 | 3 | 3 | 3 | 3 |
| Coenzyme transport and metabolism | 185 | 248 | 253 | 242 | 248 |
| Cytoskeleton | 0 | 1 | 1 | 1 | 1 |
| Defense mechanisms | 70 | 120 | 135 | 116 | 125 |
| Energy production and conversion | 327 | 311 | 343 | 315 | 318 |
| Extracellular structures | 0 | 54 | 56 | 43 | 53 |
| Function unknown | 496 | 303 | 319 | 312 | 320 |
| General function prediction only | 716 | 454 | 501 | 457 | 481 |
| Inorganic ion transport and metabolism | 353 | 304 | 325 | 285 | 307 |
| Intracellular trafficking, secretion, and vesicular transport | 165 | 146 | 146 | 136 | 140 |
| Lipid transport and metabolism | 245 | 268 | 284 | 267 | 275 |
| Nucleotide transport and metabolism | 98 | 116 | 114 | 115 | 114 |
| Posttranslational modification, protein turnover, chaperones | 196 | 191 | 221 | 188 | 193 |
| RNA processing and modification | 2 | 2 | 2 | 2 | 2 |
| Replication, recombination and repair | 173 | 115 | 147 | 109 | 113 |
| Secondary metabolites biosynthesis, transport and catabolism | 198 | 160 | 194 | 166 | 177 |
| Signal transduction mechanisms | 326 | 328 | 342 | 311 | 329 |
| Transcription | 495 | 457 | 505 | 456 | 466 |
| Translation, ribosomal structure and biogenesis | 194 | 255 | 261 | 252 | 259 |
