# Supplementary material for: Genome Sequence and Metabolic Analysis of a Fluoranthene-Degrading Strain Pseudomonas aeruginosa DN1
Source: Front Microbiol. 2018 Oct 31;9:2595. doi: 10.3389/fmicb.2018.02595 (PMC6220107; doi:10.3389/fmicb.2018.02595)
Supplement: Supplementary file 11 [file Table_11.DOCX]

**Table S11 | Predicted genomic islands (GIs) and genes in the GIs**

GIs predicted using different methods (Sheet 1); genes in the GIs predicted using the SIGI-HMM method (Sheet 2)

**Sheet 1(Excel)**

| **Start** | **End** | **Size** | **CDS No.** | **GI Prediction Program** |
| --- | --- | --- | --- | --- |
| **Chromosome** | |  |  |  |
| 4346597 | 4355562 | 8965 | 9 | IslandPath-DIMOB |
| 5403715 | 5426422 | 22707 | 19 | IslandPath-DIMOB |
| 55330 | 63077 | 7747 | 2 | SIGI-HMM |
| 284700 | 294820 | 10120 | 20 | SIGI-HMM |
| 628743 | 645447 | 16704 | 8 | SIGI-HMM |
| 692042 | 697559 | 5517 | 7 | SIGI-HMM |
| 722664 | 737296 | 14632 | 11 | SIGI-HMM |
| 750507 | 764144 | 13637 | 28 | SIGI-HMM |
| 819100 | 827666 | 8566 | 20 | SIGI-HMM |
| 836923 | 843155 | 6232 | 4 | SIGI-HMM |
| 1213548 | 1220416 | 6868 | 6 | SIGI-HMM |
| 1226487 | 1234332 | 7845 | 6 | SIGI-HMM |
| 1696645 | 1701233 | 4588 | 7 | SIGI-HMM |
| 1712727 | 1720073 | 7346 | 11 | SIGI-HMM |
| 1726607 | 1735568 | 8961 | 13 | SIGI-HMM |
| 1745301 | 1749833 | 4532 | 7 | SIGI-HMM |
| 1751407 | 1757440 | 6033 | 5 | SIGI-HMM |
| 1793903 | 1800788 | 6885 | 7 | SIGI-HMM |
| 1816369 | 1820559 | 4190 | 3 | SIGI-HMM |
| 1903491 | 1912310 | 8819 | 11 | SIGI-HMM |
| 1977237 | 2001579 | 24342 | 14 | SIGI-HMM |
| 2235359 | 2241783 | 6424 | 10 | SIGI-HMM |
| 2243075 | 2248120 | 5045 | 4 | SIGI-HMM |
| 2440499 | 2446798 | 6299 | 2 | SIGI-HMM |
| 2510058 | 2514671 | 4613 | 6 | SIGI-HMM |
| 2682701 | 2687350 | 4649 | 5 | SIGI-HMM |
| 3116839 | 3128351 | 11512 | 7 | SIGI-HMM |
| 3133107 | 3143125 | 10018 | 6 | SIGI-HMM |
| 3199192 | 3203256 | 4064 | 3 | SIGI-HMM |
| 3216007 | 3223664 | 7657 | 2 | SIGI-HMM |
| 3632591 | 3638810 | 6219 | 4 | SIGI-HMM |
| 3650541 | 3656633 | 6092 | 17 | SIGI-HMM |
| 4222821 | 4229951 | 7130 | 6 | SIGI-HMM |
| 4479190 | 4486799 | 7609 | 5 | SIGI-HMM |
| 4657800 | 4713603 | 55803 | 40 | SIGI-HMM |
| 4714948 | 4719730 | 4782 | 8 | SIGI-HMM |
| 4730286 | 4736978 | 6692 | 10 | SIGI-HMM |
| 4873096 | 4878923 | 5827 | 8 | SIGI-HMM |
| 5246852 | 5252649 | 5797 | 8 | SIGI-HMM |
| 5362668 | 5374555 | 11887 | 14 | SIGI-HMM |
| 5375574 | 5390150 | 14576 | 16 | SIGI-HMM |
| 5401375 | 5427763 | 26388 | 23 | SIGI-HMM |
| 5447362 | 5453405 | 6043 | 2 | SIGI-HMM |
| 5631650 | 5639824 | 8174 | 3 | SIGI-HMM |
| 5699905 | 5704102 | 4197 | 4 | SIGI-HMM |
| 6303213 | 6307684 | 4471 | 3 | SIGI-HMM |
| 284611 | 288864 | 4253 | 4 | IslandPick |
| 289097 | 295209 | 6112 | 10 | IslandPick |
| 630468 | 636535 | 6067 | 1 | IslandPick |
| 812338 | 850465 | 38127 | 52 | IslandPick |
| 1016941 | 1022211 | 5270 | 4 | IslandPick |
| 1060652 | 1072837 | 12185 | 13 | IslandPick |
| 1313038 | 1317058 | 4020 | 2 | IslandPick |
| 1903809 | 1909978 | 6169 | 9 | IslandPick |
| 1978769 | 1988311 | 9542 | 6 | IslandPick |
| 1988505 | 1999472 | 10967 | 5 | IslandPick |
| 3133943 | 3139200 | 5257 | 4 | IslandPick |
| 3625551 | 3663360 | 37809 | 51 | IslandPick |
| 4233449 | 4237746 | 4297 | 6 | IslandPick |
| 4403984 | 4417320 | 13336 | 11 | IslandPick |
| 4673901 | 4689535 | 15634 | 7 | IslandPick |
| 5412685 | 5422997 | 10312 | 3 | IslandPick |
| 6101173 | 6105637 | 4464 | 2 | IslandPick |
| Plasmid | |  |  |  |
| 273903 | 279751 | 5848 | 7 | SIGI-HMM |

**Sheet 2(Excel)**

| **Start** | | **End** | | **Size** | **Locus_DN1_** | **Product Name** |
| --- | --- | --- | --- | --- | --- | --- |
| **Chromosome** | | | | |  |  |
| 55330 | 63077 | | 7747 | | DN1_orf00060 | hemagglutinin |
| 55330 | 63077 | | 7747 | | DN1_orf00062 | hypothetical protein |
| 284700 | 294820 | | 10120 | | DN1_orf00367 | hypothetical protein |
| 284700 | 294820 | | 10120 | | DN1_orf00368 | hypothetical protein |
| 284700 | 294820 | | 10120 | | DN1_orf00369 | ATP-dependent endonuclease of the OLD family-like protein |
| 284700 | 294820 | | 10120 | | DN1_orf00371 | hypothetical protein |
| 284700 | 294820 | | 10120 | | DN1_orf00372 | hypothetical protein |
| 284700 | 294820 | | 10120 | | DN1_orf00373 | hypothetical protein |
| 284700 | 294820 | | 10120 | | DN1_orf00374 | hypothetical protein |
| 284700 | 294820 | | 10120 | | DN1_orf00375 | hypothetical protein |
| 284700 | 294820 | | 10120 | | DN1_orf00376 | hypothetical protein |
| 284700 | 294820 | | 10120 | | DN1_orf00377 | hypothetical protein |
| 284700 | 294820 | | 10120 | | DN1_orf00378 | hypothetical protein |
| 284700 | 294820 | | 10120 | | DN1_orf00379 | hypothetical protein |
| 628743 | 645447 | | 16704 | | DN1_orf00846 | hypothetical protein |
| 628743 | 645447 | | 16704 | | DN1_orf00847 | hypothetical protein |
| 628743 | 645447 | | 16704 | | DN1_orf00848 | putative transposase |
| 628743 | 645447 | | 16704 | | DN1_orf00849 | transposase |
| 628743 | 645447 | | 16704 | | DN1_orf00850 | hypothetical protein |
| 628743 | 645447 | | 16704 | | DN1_orf00851 | hypothetical protein |
| 628743 | 645447 | | 16704 | | DN1_orf00852 | replication protein |
| 628743 | 645447 | | 16704 | | DN1_orf00853 | site-specific recombinase, phage integrase family |
| 692042 | 697559 | | 5517 | | DN1_orf00910 | hypothetical protein |
| 692042 | 697559 | | 5517 | | DN1_orf00911 | putative baseplate assembly protein V |
| 692042 | 697559 | | 5517 | | DN1_orf00912 | bacteriophage protein |
| 692042 | 697559 | | 5517 | | DN1_orf00913 | bacteriophage protein |
| 692042 | 697559 | | 5517 | | DN1_orf00914 | putative phage tail protein |
| 692042 | 697559 | | 5517 | | DN1_orf00916 | tail fiber |
| 692042 | 697559 | | 5517 | | DN1_orf00918 | tail fiber assembly protein |
| 722664 | 737296 | | 14632 | | DN1_orf00955 | tyrosyl-tRNA synthetase |
| 722664 | 737296 | | 14632 | | DN1_orf00957 | Biotin-protein ligase / Biotin operon repressor |
| 722664 | 737296 | | 14632 | | DN1_orf00958 | pantothenate kinase |
| 722664 | 737296 | | 14632 | | DN1_orf00959 | hypothetical protein |
| 722664 | 737296 | | 14632 | | DN1_orf00960 | elongation factor Tu |
| 722664 | 737296 | | 14632 | | DN1_orf00961 | transcription antitermination protein NusG |
| 722664 | 737296 | | 14632 | | DN1_orf00962 | secretion protein SecE |
| 722664 | 737296 | | 14632 | | DN1_orf00963 | 50S ribosomal protein L11 |
| 722664 | 737296 | | 14632 | | DN1_orf00964 | 50S ribosomal protein L1 |
| 722664 | 737296 | | 14632 | | DN1_orf00965 | 50S ribosomal protein L10 |
| 722664 | 737296 | | 14632 | | DN1_orf00966 | 50S ribosomal protein L7/L12 |
| 750507 | 764144 | | 13637 | | DN1_orf00976 | 30S ribosomal protein S10 |
| 750507 | 764144 | | 13637 | | DN1_orf00977 | 50S ribosomal protein L3 |
| 750507 | 764144 | | 13637 | | DN1_orf00978 | 50S ribosomal protein L4 |
| 750507 | 764144 | | 13637 | | DN1_orf00979 | 50S ribosomal protein L23 |
| 750507 | 764144 | | 13637 | | DN1_orf00981 | 50S ribosomal protein L2 |
| 750507 | 764144 | | 13637 | | DN1_orf00982 | 30S ribosomal protein S19 |
| 750507 | 764144 | | 13637 | | DN1_orf00983 | 50S ribosomal protein L22 |
| 750507 | 764144 | | 13637 | | DN1_orf00984 | 30S ribosomal protein S3 |
| 750507 | 764144 | | 13637 | | DN1_orf00985 | 50S ribosomal protein L16 |
| 750507 | 764144 | | 13637 | | DN1_orf00986 | 50S ribosomal protein L29 |
| 750507 | 764144 | | 13637 | | DN1_orf00987 | 30S ribosomal protein S17 |
| 750507 | 764144 | | 13637 | | DN1_orf00988 | 50S ribosomal protein L14 |
| 750507 | 764144 | | 13637 | | DN1_orf00989 | 50S ribosomal protein L24 |
| 750507 | 764144 | | 13637 | | DN1_orf00990 | 50S ribosomal protein L5 |
| 750507 | 764144 | | 13637 | | DN1_orf00991 | 30S ribosomal protein S14 |
| 750507 | 764144 | | 13637 | | DN1_orf00992 | 30S ribosomal protein S8 |
| 750507 | 764144 | | 13637 | | DN1_orf00993 | 50S ribosomal protein L6 |
| 750507 | 764144 | | 13637 | | DN1_orf00994 | 50S ribosomal protein L18 |
| 750507 | 764144 | | 13637 | | DN1_orf00995 | 30S ribosomal protein S5 |
| 750507 | 764144 | | 13637 | | DN1_orf00996 | 50S ribosomal protein L30 |
| 750507 | 764144 | | 13637 | | DN1_orf00997 | 50S ribosomal protein L15 |
| 750507 | 764144 | | 13637 | | DN1_orf00998 | preprotein translocase subunit SecY |
| 750507 | 764144 | | 13637 | | DN1_orf01000 | Preprotein translocase subunit secY |
| 750507 | 764144 | | 13637 | | DN1_orf01001 | 30S ribosomal protein S13 |
| 750507 | 764144 | | 13637 | | DN1_orf01002 | hypothetical protein |
| 750507 | 764144 | | 13637 | | DN1_orf01003 | 30S ribosomal protein S4 |
| 750507 | 764144 | | 13637 | | DN1_orf01004 | DNA-directed RNA polymerase subunit alpha |
| 750507 | 764144 | | 13637 | | DN1_orf01005 | 50S ribosomal protein L17 |
| 819100 | 827666 | | 8566 | | DN1_orf01070 | hypothetical protein |
| 819100 | 827666 | | 8566 | | DN1_orf01071 | hypothetical protein |
| 819100 | 827666 | | 8566 | | DN1_orf01072 | hypothetical protein |
| 819100 | 827666 | | 8566 | | DN1_orf01073 | hypothetical protein |
| 819100 | 827666 | | 8566 | | DN1_orf01074 | hypothetical protein |
| 819100 | 827666 | | 8566 | | DN1_orf01075 | hypothetical protein |
| 819100 | 827666 | | 8566 | | DN1_orf01076 | hypothetical protein |
| 819100 | 827666 | | 8566 | | DN1_orf01077 | hypothetical protein |
| 819100 | 827666 | | 8566 | | DN1_orf01078 | hypothetical protein |
| 819100 | 827666 | | 8566 | | DN1_orf01079 | hypothetical protein predicted by Glimmer/Critica |
| 819100 | 827666 | | 8566 | | DN1_orf01080 | hypothetical protein |
| 819100 | 827666 | | 8566 | | DN1_orf01081 | hypothetical protein |
| 819100 | 827666 | | 8566 | | DN1_orf01082 | hypothetical protein |
| 819100 | 827666 | | 8566 | | DN1_orf01083 | putative structural protein |
| 819100 | 827666 | | 8566 | | DN1_orf01084 | hypothetical protein |
| 819100 | 827666 | | 8566 | | DN1_orf01085 | hypothetical protein |
| 819100 | 827666 | | 8566 | | DN1_orf01086 | hypothetical protein |
| 819100 | 827666 | | 8566 | | DN1_orf01087 | hypothetical protein predicted by Glimmer/Critica |
| 819100 | 827666 | | 8566 | | DN1_orf01088 | DNA-binding protein |
| 819100 | 827666 | | 8566 | | DN1_orf01089 | hypothetical protein |
| 836923 | 843155 | | 6232 | | DN1_orf01107 | hypothetical protein |
| 836923 | 843155 | | 6232 | | DN1_orf01108 | tape measure domain-containing protein |
| 836923 | 843155 | | 6232 | | DN1_orf01109 | hypothetical protein |
| 836923 | 843155 | | 6232 | | DN1_orf01110 | hypothetical protein |
| 1213548 | 1220416 | | 6868 | | DN1_orf01610 | hydrolase |
| 1213548 | 1220416 | | 6868 | | DN1_orf01611 | aromatic-ring hyroxylase |
| 1213548 | 1220416 | | 6868 | | DN1_orf01612 | hypothetical protein |
| 1213548 | 1220416 | | 6868 | | DN1_orf01613 | hypothetical protein |
| 1213548 | 1220416 | | 6868 | | DN1_orf01614 | phytoene dehydrogenase |
| 1213548 | 1220416 | | 6868 | | DN1_orf01615 | hypothetical protein |
| 1226487 | 1234332 | | 7845 | | DN1_orf01620 | hypothetical protein |
| 1226487 | 1234332 | | 7845 | | DN1_orf01621 | hypothetical protein |
| 1226487 | 1234332 | | 7845 | | DN1_orf01622 | hypothetical protein |
| 1226487 | 1234332 | | 7845 | | DN1_orf01623 | hypothetical protein |
| 1226487 | 1234332 | | 7845 | | DN1_orf01624 | hypothetical protein |
| 1226487 | 1234332 | | 7845 | | DN1_orf01625 | hypothetical protein |
| 1696645 | 1701233 | | 4588 | | DN1_orf02170 | hypothetical protein |
| 1696645 | 1701233 | | 4588 | | DN1_orf02171 | hypothetical protein |
| 1696645 | 1701233 | | 4588 | | DN1_orf02172 | hypothetical protein |
| 1696645 | 1701233 | | 4588 | | DN1_orf02174 | hypothetical protein |
| 1696645 | 1701233 | | 4588 | | DN1_orf02175 | hypothetical phage protein |
| 1696645 | 1701233 | | 4588 | | DN1_orf02176 | hypothetical protein |
| 1696645 | 1701233 | | 4588 | | DN1_orf02177 | lytic enzyme |
| 1712727 | 1720073 | | 7346 | | DN1_orf02184 | phage minor tail protein L |
| 1712727 | 1720073 | | 7346 | | DN1_orf02186 | phage minor tail protein |
| 1712727 | 1720073 | | 7346 | | DN1_orf02189 | phage tail length tape measure protein |
| 1712727 | 1720073 | | 7346 | | DN1_orf02190 | phage protein |
| 1712727 | 1720073 | | 7346 | | DN1_orf02191 | hypothetical protein |
| 1712727 | 1720073 | | 7346 | | DN1_orf02192 | phage tail protein |
| 1712727 | 1720073 | | 7346 | | DN1_orf02193 | hypothetical protein |
| 1712727 | 1720073 | | 7346 | | DN1_orf02194 | hypothetical protein |
| 1712727 | 1720073 | | 7346 | | DN1_orf02195 | hypothetical protein |
| 1712727 | 1720073 | | 7346 | | DN1_orf02196 | hypothetical protein |
| 1712727 | 1720073 | | 7346 | | DN1_orf02197 | hypothetical protein |
| 1726607 | 1735568 | | 8961 | | DN1_orf02206 | hypothetical protein |
| 1726607 | 1735568 | | 8961 | | DN1_orf02207 | hypothetical protein |
| 1726607 | 1735568 | | 8961 | | DN1_orf02208 | hypothetical protein |
| 1726607 | 1735568 | | 8961 | | DN1_orf02209 | hypothetical protein |
| 1726607 | 1735568 | | 8961 | | DN1_orf02210 | hypothetical protein |
| 1726607 | 1735568 | | 8961 | | DN1_orf02212 | endodeoxyribonuclease RusA |
| 1726607 | 1735568 | | 8961 | | DN1_orf02213 | hypothetical protein |
| 1726607 | 1735568 | | 8961 | | DN1_orf02214 | hypothetical protein |
| 1726607 | 1735568 | | 8961 | | DN1_orf02215 | hypothetical protein |
| 1726607 | 1735568 | | 8961 | | DN1_orf02216 | hypothetical protein |
| 1726607 | 1735568 | | 8961 | | DN1_orf02217 | Cro/CI family transcriptional regulator |
| 1726607 | 1735568 | | 8961 | | DN1_orf02218 | DNA-damage-inducible protein d |
| 1726607 | 1735568 | | 8961 | | DN1_orf02219 | hypothetical protein |
| 1745301 | 1749833 | | 4532 | | DN1_orf02231 | hypothetical protein |
| 1745301 | 1749833 | | 4532 | | DN1_orf02234 | DNA-cytosine methyltransferase |
| 1745301 | 1749833 | | 4532 | | DN1_orf02235 | hypothetical protein |
| 1745301 | 1749833 | | 4532 | | DN1_orf02236 | hypothetical protein |
| 1745301 | 1749833 | | 4532 | | DN1_orf02237 | hypothetical protein |
| 1745301 | 1749833 | | 4532 | | DN1_orf02238 | hypothetical protein |
| 1745301 | 1749833 | | 4532 | | DN1_orf02240 | hypothetical protein |
| 1751407 | 1757440 | | 6033 | | DN1_orf02243 | hypothetical transport protein |
| 1751407 | 1757440 | | 6033 | | DN1_orf02244 | integrase |
| 1751407 | 1757440 | | 6033 | | DN1_orf02245 | osmoprotectant transporter activator protein |
| 1751407 | 1757440 | | 6033 | | DN1_orf02248 | hypothetical protein |
| 1751407 | 1757440 | | 6033 | | DN1_orf02249 | hypothetical protein |
| 1793903 | 1800788 | | 6885 | | DN1_orf02287 | hypothetical protein |
| 1793903 | 1800788 | | 6885 | | DN1_orf02288 | hypothetical protein |
| 1793903 | 1800788 | | 6885 | | DN1_orf02289 | phage minor tail protein L |
| 1793903 | 1800788 | | 6885 | | DN1_orf02290 | phage minor tail protein |
| 1793903 | 1800788 | | 6885 | | DN1_orf02292 | hypothetical protein |
| 1793903 | 1800788 | | 6885 | | DN1_orf02293 | hypothetical protein |
| 1793903 | 1800788 | | 6885 | | DN1_orf02294 | hypothetical protein |
| 1816369 | 1820559 | | 4190 | | DN1_orf02317 | putative DNA-binding protein |
| 1816369 | 1820559 | | 4190 | | DN1_orf02318 | hypothetical protein, partial |
| 1816369 | 1820559 | | 4190 | | DN1_orf02320 | hypothetical protein |
| 1903491 | 1912310 | | 8819 | | DN1_orf02439 | hypothetical protein |
| 1903491 | 1912310 | | 8819 | | DN1_orf02440 | hypothetical protein |
| 1903491 | 1912310 | | 8819 | | DN1_orf02441 | hypothetical protein |
| 1903491 | 1912310 | | 8819 | | DN1_orf02442 | hypothetical protein |
| 1903491 | 1912310 | | 8819 | | DN1_orf02443 | hypothetical protein |
| 1903491 | 1912310 | | 8819 | | DN1_orf02445 | type II secretion system protein |
| 1903491 | 1912310 | | 8819 | | DN1_orf02446 | type II secretion system protein |
| 1903491 | 1912310 | | 8819 | | DN1_orf02447 | type II secretion system protein |
| 1903491 | 1912310 | | 8819 | | DN1_orf02448 | type II secretion system protein |
| 1903491 | 1912310 | | 8819 | | DN1_orf02450 | type II secretion protein |
| 1903491 | 1912310 | | 8819 | | DN1_orf02451 | ABC-2 transporter permease |
| 1977237 | 2001579 | | 24342 | | DN1_orf02530 | DNA-cytosine methyltransferase |
| 1977237 | 2001579 | | 24342 | | DN1_orf02531 | hypothetical protein |
| 1977237 | 2001579 | | 24342 | | DN1_orf02532 | hypothetical protein |
| 1977237 | 2001579 | | 24342 | | DN1_orf02533 | hypothetical protein |
| 1977237 | 2001579 | | 24342 | | DN1_orf02534 | transposase |
| 1977237 | 2001579 | | 24342 | | DN1_orf02535 | hypothetical protein |
| 1977237 | 2001579 | | 24342 | | DN1_orf02536 | hypothetical protein |
| 1977237 | 2001579 | | 24342 | | DN1_orf02537 | hypothetical protein |
| 1977237 | 2001579 | | 24342 | | DN1_orf02538 | hypothetical protein |
| 1977237 | 2001579 | | 24342 | | DN1_orf02539 | hypothetical protein |
| 1977237 | 2001579 | | 24342 | | DN1_orf02540 | hypothetical protein |
| 1977237 | 2001579 | | 24342 | | DN1_orf02541 | phage integrase |
| 1977237 | 2001579 | | 24342 | | DN1_orf02542 | transcriptional regulator |
| 1977237 | 2001579 | | 24342 | | DN1_orf02543 | integration host factor subunit alpha |
| 2235359 | 2241783 | | 6424 | | DN1_orf02891 | acyl carrier protein |
| 2235359 | 2241783 | | 6424 | | DN1_orf02893 | 3-ketoacyl-ACP reductase |
| 2235359 | 2241783 | | 6424 | | DN1_orf02894 | malonyl-CoA-ACP transacylase |
| 2235359 | 2241783 | | 6424 | | DN1_orf02895 | Phosphate acyltransferase/ Acyl-ACP phosphotransacylase |
| 2235359 | 2241783 | | 6424 | | DN1_orf02896 | 50S ribosomal protein L32 |
| 2235359 | 2241783 | | 6424 | | DN1_orf02897 | hypothetical protein |
| 2235359 | 2241783 | | 6424 | | DN1_orf02898 | Maf-like protein |
| 2235359 | 2241783 | | 6424 | | DN1_orf02899 | peptidase |
| 2235359 | 2241783 | | 6424 | | DN1_orf02900 | putative hydrolase |
| 2235359 | 2241783 | | 6424 | | DN1_orf02901 | ribosomal large subunit pseudouridine synthase C |
| 2243075 | 2248120 | | 5045 | | DN1_orf02902 | ribonuclease E |
| 2243075 | 2248120 | | 5045 | | DN1_orf02903 | hypothetical protein |
| 2243075 | 2248120 | | 5045 | | DN1_orf02904 | UDP-N-acetylenolpyruvoylglucosamine reductase |
| 2243075 | 2248120 | | 5045 | | DN1_orf02905 | phosphotyrosine protein phosphatase |
| 2440499 | 2446798 | | 6299 | | DN1_orf03135 | hypothetical protein |
| 2440499 | 2446798 | | 6299 | | DN1_orf03136 | integration host factor subunit beta |
| 2510058 | 2514671 | | 4613 | | DN1_orf03226 | LysR family transcriptional regulator |
| 2510058 | 2514671 | | 4613 | | DN1_orf03227 | putative flavodoxin |
| 2510058 | 2514671 | | 4613 | | DN1_orf03228 | glutathione S-transferase |
| 2510058 | 2514671 | | 4613 | | DN1_orf03229 | hypothetical protein |
| 2510058 | 2514671 | | 4613 | | DN1_orf03230 | hypothetical protein |
| 2510058 | 2514671 | | 4613 | | DN1_orf03231 | protein-disulfide isomerase |
| 2682701 | 2687350 | | 4649 | | DN1_orf03444 | hypothetical protein, partial |
| 2682701 | 2687350 | | 4649 | | DN1_orf03445 | hypothetical protein |
| 2682701 | 2687350 | | 4649 | | DN1_orf03446 | NADH oxidase |
| 2682701 | 2687350 | | 4649 | | DN1_orf03447 | hypothetical protein |
| 2682701 | 2687350 | | 4649 | | DN1_orf03449 | hypothetical protein |
| 3116839 | 3128351 | | 11512 | | DN1_orf04016 | hypothetical protein |
| 3116839 | 3128351 | | 11512 | | DN1_orf04017 | DNA-binding protein |
| 3116839 | 3128351 | | 11512 | | DN1_orf04018 | hypothetical protein |
| 3116839 | 3128351 | | 11512 | | DN1_orf04020 | hypothetical protein |
| 3116839 | 3128351 | | 11512 | | DN1_orf04021 | antirestriction protein family protein |
| 3116839 | 3128351 | | 11512 | | DN1_orf04022 | cobyrinic acid a,c-diamide synthase |
| 3116839 | 3128351 | | 11512 | | DN1_orf04023 | hypothetical protein |
| 3133107 | 3143125 | | 10018 | | DN1_orf04026 | abortive infection bacteriophage resistance protein |
| 3133107 | 3143125 | | 10018 | | DN1_orf04027 | type I restriction-modification system, M subunit |
| 3133107 | 3143125 | | 10018 | | DN1_orf04028 | Type I restriction-modification system, specificity subunit S |
| 3133107 | 3143125 | | 10018 | | DN1_orf04030 | AAA ATPase domain protein |
| 3133107 | 3143125 | | 10018 | | DN1_orf04031 | phage transcriptional regulator AlpA |
| 3133107 | 3143125 | | 10018 | | DN1_orf04032 | phage integrase family protein |
| 3199192 | 3203256 | | 4064 | | DN1_orf04111 | S-adenosylmethionine:trna ribosyltransferase-isomerase |
| 3199192 | 3203256 | | 4064 | | DN1_orf04112 | S-adenosylmethionine--tRNA ribosyltransferase-isomerase |
| 3199192 | 3203256 | | 4064 | | DN1_orf04113 | transposase |
| 3216007 | 3223664 | | 7657 | | DN1_orf04128 | hypothetical protein |
| 3216007 | 3223664 | | 7657 | | DN1_orf04131 | rhs family protein |
| 3632591 | 3638810 | | 6219 | | DN1_orf04676 | hypothetical protein |
| 3632591 | 3638810 | | 6219 | | DN1_orf04677 | hypothetical protein |
| 3632591 | 3638810 | | 6219 | | DN1_orf04679 | hypothetical protein |
| 3632591 | 3638810 | | 6219 | | DN1_orf04680 | hypothetical protein |
| 3650541 | 3656633 | | 6092 | | DN1_orf04701 | hypothetical protein |
| 3650541 | 3656633 | | 6092 | | DN1_orf04702 | hypothetical protein |
| 3650541 | 3656633 | | 6092 | | DN1_orf04703 | hypothetical protein |
| 3650541 | 3656633 | | 6092 | | DN1_orf04704 | putative structural protein |
| 3650541 | 3656633 | | 6092 | | DN1_orf04705 | hypothetical protein |
| 3650541 | 3656633 | | 6092 | | DN1_orf04706 | hypothetical protein |
| 3650541 | 3656633 | | 6092 | | DN1_orf04707 | hypothetical protein |
| 3650541 | 3656633 | | 6092 | | DN1_orf04708 | hypothetical protein |
| 3650541 | 3656633 | | 6092 | | DN1_orf04709 | hypothetical protein |
| 3650541 | 3656633 | | 6092 | | DN1_orf04710 | hypothetical protein |
| 3650541 | 3656633 | | 6092 | | DN1_orf04711 | hypothetical protein |
| 3650541 | 3656633 | | 6092 | | DN1_orf04712 | hypothetical protein |
| 3650541 | 3656633 | | 6092 | | DN1_orf04713 | Mu-like prophage host-nuclease inhibitor protein |
| 3650541 | 3656633 | | 6092 | | DN1_orf04714 | hypothetical protein |
| 3650541 | 3656633 | | 6092 | | DN1_orf04715 | hypothetical protein |
| 3650541 | 3656633 | | 6092 | | DN1_orf04716 | hypothetical protein |
| 3650541 | 3656633 | | 6092 | | DN1_orf04717 | hypothetical protein |
| 4222821 | 4229951 | | 7130 | | DN1_orf05485 | hypothetical protein |
| 4222821 | 4229951 | | 7130 | | DN1_orf05486 | lipase family protein |
| 4222821 | 4229951 | | 7130 | | DN1_orf05487 | lipoprotein |
| 4222821 | 4229951 | | 7130 | | DN1_orf05488 | hypothetical protein |
| 4222821 | 4229951 | | 7130 | | DN1_orf05489 | hypothetical protein |
| 4222821 | 4229951 | | 7130 | | DN1_orf05490 | Rhs element Vgr protein |
| 4479190 | 4486799 | | 7609 | | DN1_orf05842 | hypothetical protein |
| 4479190 | 4486799 | | 7609 | | DN1_orf05843 | hypothetical protein |
| 4479190 | 4486799 | | 7609 | | DN1_orf05845 | pyocin S2 |
| 4479190 | 4486799 | | 7609 | | DN1_orf05846 | hypothetical protein |
| 4479190 | 4486799 | | 7609 | | DN1_orf05847 | hypothetical protein |
| 4657800 | 4713603 | | 55803 | | DN1_orf06072 | Methylated-DNA--protein-cysteine methyltransferase |
| 4657800 | 4713603 | | 55803 | | DN1_orf06074 | usher CupC3 |
| 4657800 | 4713603 | | 55803 | | DN1_orf06076 | fimbrial subunit CupC1 |
| 4657800 | 4713603 | | 55803 | | DN1_orf06077 | hypothetical protein |
| 4657800 | 4713603 | | 55803 | | DN1_orf06078 | hypothetical protein |
| 4657800 | 4713603 | | 55803 | | DN1_orf06079 | ExoU |
| 4657800 | 4713603 | | 55803 | | DN1_orf06080 | putative transposase |
| 4657800 | 4713603 | | 55803 | | DN1_orf06081 | hypothetical protein |
| 4657800 | 4713603 | | 55803 | | DN1_orf06083 | conserved hypothetical protein |
| 4657800 | 4713603 | | 55803 | | DN1_orf06084 | hypothetical protein |
| 4657800 | 4713603 | | 55803 | | DN1_orf06085 | hypothetical protein |
| 4657800 | 4713603 | | 55803 | | DN1_orf06086 | hypothetical protein |
| 4657800 | 4713603 | | 55803 | | DN1_orf06087 | transposase, mutator type |
| 4657800 | 4713603 | | 55803 | | DN1_orf06088 | prolyl-tRNA synthetase |
| 4657800 | 4713603 | | 55803 | | DN1_orf06089 | transporter, major facilitator family |
| 4657800 | 4713603 | | 55803 | | DN1_orf06092 | amino acid adenylation enzyme/thioester reductase family protein |
| 4657800 | 4713603 | | 55803 | | DN1_orf06093 | amino acid adenylation domain protein |
| 4657800 | 4713603 | | 55803 | | DN1_orf06095 | ISPsy2, transposase, partial |
| 4657800 | 4713603 | | 55803 | | DN1_orf06096 | transposase |
| 4657800 | 4713603 | | 55803 | | DN1_orf06097 | putative transposase |
| 4657800 | 4713603 | | 55803 | | DN1_orf06098 | addiction module antidote family protein |
| 4657800 | 4713603 | | 55803 | | DN1_orf06099 | plasmid stablization protein |
| 4657800 | 4713603 | | 55803 | | DN1_orf06101 | hypothetical protein |
| 4657800 | 4713603 | | 55803 | | DN1_orf06102 | glutathione-regulated potassium-proton antiporter |
| 4657800 | 4713603 | | 55803 | | DN1_orf06103 | short chain dehydrogenase |
| 4657800 | 4713603 | | 55803 | | DN1_orf06104 | short chain dehydrogenase/reductase family oxidoreductase |
| 4657800 | 4713603 | | 55803 | | DN1_orf06105 | short chain dehydrogenase/reductase family oxidoreductase |
| 4657800 | 4713603 | | 55803 | | DN1_orf06106 | aldehyde dehydrogenase |
| 4657800 | 4713603 | | 55803 | | DN1_orf06107 | class II aldolase |
| 4657800 | 4713603 | | 55803 | | DN1_orf06108 | LysR family transcriptional regulator |
| 4657800 | 4713603 | | 55803 | | DN1_orf06109 | choline dehydrogenase |
| 4657800 | 4713603 | | 55803 | | DN1_orf06110 | Zinc-dependent hydroxylase |
| 4657800 | 4713603 | | 55803 | | DN1_orf06111 | gentisate 1,2-dioxygenase |
| 4657800 | 4713603 | | 55803 | | DN1_orf06113 | 2-polyprenyl-6-methoxyphenol hydroxylase-like oxidoreductase |
| 4657800 | 4713603 | | 55803 | | DN1_orf06114 | relaxase |
| 4657800 | 4713603 | | 55803 | | DN1_orf06115 | phage integrase family site specific recombinase |
| 4657800 | 4713603 | | 55803 | | DN1_orf06116 | hypothetical protein |
| 4657800 | 4713603 | | 55803 | | DN1_orf06117 | radical activating enzyme |
| 4657800 | 4713603 | | 55803 | | DN1_orf06118 | hypothetical protein |
| 4657800 | 4713603 | | 55803 | | DN1_orf06120 | Peptidoglycan associated lipoprotein OprL precursor |
| 4714948 | 4719730 | | 4782 | | DN1_orf06121 | translocation protein TolB |
| 4714948 | 4719730 | | 4782 | | DN1_orf06122 | TolA protein |
| 4714948 | 4719730 | | 4782 | | DN1_orf06123 | TolR protein |
| 4714948 | 4719730 | | 4782 | | DN1_orf06124 | TolQ protein |
| 4714948 | 4719730 | | 4782 | | DN1_orf06125 | hypothetical protein |
| 4714948 | 4719730 | | 4782 | | DN1_orf06126 | Holliday junction DNA helicase RuvB |
| 4714948 | 4719730 | | 4782 | | DN1_orf06127 | Holliday junction DNA helicase RuvA |
| 4714948 | 4719730 | | 4782 | | DN1_orf06128 | Holliday junction resolvase, partial |
| 4730286 | 4736978 | | 6692 | | DN1_orf06141 | putative acylphosphatase |
| 4730286 | 4736978 | | 6692 | | DN1_orf06142 | thioredoxin, partial |
| 4730286 | 4736978 | | 6692 | | DN1_orf06143 | hypothetical protein |
| 4730286 | 4736978 | | 6692 | | DN1_orf06144 | hypothetical protein |
| 4730286 | 4736978 | | 6692 | | DN1_orf06145 | ribonuclease |
| 4730286 | 4736978 | | 6692 | | DN1_orf06146 | putative arsenate reductase |
| 4730286 | 4736978 | | 6692 | | DN1_orf06148 | Trp repressor binding protein |
| 4730286 | 4736978 | | 6692 | | DN1_orf06150 | hypothetical protein |
| 4730286 | 4736978 | | 6692 | | DN1_orf06151 | DNA replication initiation factor |
| 4730286 | 4736978 | | 6692 | | DN1_orf06152 | hypothetical protein |
| 4873096 | 4878923 | | 5827 | | DN1_orf06331 | hypothetical protein |
| 4873096 | 4878923 | | 5827 | | DN1_orf06332 | hypothetical protein |
| 4873096 | 4878923 | | 5827 | | DN1_orf06333 | hypothetical protein |
| 4873096 | 4878923 | | 5827 | | DN1_orf06334 | hypothetical protein |
| 4873096 | 4878923 | | 5827 | | DN1_orf06335 | hypothetical protein |
| 4873096 | 4878923 | | 5827 | | DN1_orf06336 | transposase |
| 4873096 | 4878923 | | 5827 | | DN1_orf06337 | putative transposase |
| 4873096 | 4878923 | | 5827 | | DN1_orf06338 | hypothetical protein |
| 5246852 | 5252649 | | 5797 | | DN1_orf06817 | hypothetical protein |
| 5246852 | 5252649 | | 5797 | | DN1_orf06818 | hypothetical protein |
| 5246852 | 5252649 | | 5797 | | DN1_orf06819 | hypothetical protein |
| 5246852 | 5252649 | | 5797 | | DN1_orf06820 | hypothetical protein |
| 5246852 | 5252649 | | 5797 | | DN1_orf06821 | hypothetical protein |
| 5246852 | 5252649 | | 5797 | | DN1_orf06822 | hypothetical protein |
| 5246852 | 5252649 | | 5797 | | DN1_orf06823 | PBSX family phage portal protein |
| 5246852 | 5252649 | | 5797 | | DN1_orf06824 | hypothetical protein |
| 5362668 | 5374555 | | 11887 | | DN1_orf06955 | hypothetical protein |
| 5362668 | 5374555 | | 11887 | | DN1_orf06956 | hypothetical protein |
| 5362668 | 5374555 | | 11887 | | DN1_orf06957 | hypothetical protein |
| 5362668 | 5374555 | | 11887 | | DN1_orf06958 | hypothetical protein |
| 5362668 | 5374555 | | 11887 | | DN1_orf06959 | conserved hypothetical protein |
| 5362668 | 5374555 | | 11887 | | DN1_orf06960 | hypothetical protein |
| 5362668 | 5374555 | | 11887 | | DN1_orf06961 | hypothetical protein |
| 5362668 | 5374555 | | 11887 | | DN1_orf06963 | replicative DNA helicase |
| 5362668 | 5374555 | | 11887 | | DN1_orf06964 | hypothetical protein |
| 5362668 | 5374555 | | 11887 | | DN1_orf06965 | hypothetical protein |
| 5362668 | 5374555 | | 11887 | | DN1_orf06966 | hypothetical protein |
| 5362668 | 5374555 | | 11887 | | DN1_orf06967 | hypothetical protein |
| 5362668 | 5374555 | | 11887 | | DN1_orf06968 | hypothetical protein |
| 5362668 | 5374555 | | 11887 | | DN1_orf06969 | hypothetical protein |
| 5375574 | 5390150 | | 14576 | | DN1_orf06970 | nucleoid-associated protein NdpA |
| 5375574 | 5390150 | | 14576 | | DN1_orf06971 | DNA adenine methyltransferase |
| 5375574 | 5390150 | | 14576 | | DN1_orf06972 | DNA binding protein |
| 5375574 | 5390150 | | 14576 | | DN1_orf06973 | conserved hypothetical protein |
| 5375574 | 5390150 | | 14576 | | DN1_orf06974 | hypothetical protein |
| 5375574 | 5390150 | | 14576 | | DN1_orf06976 | hypothetical protein |
| 5375574 | 5390150 | | 14576 | | DN1_orf06977 | hypothetical protein |
| 5375574 | 5390150 | | 14576 | | DN1_orf06978 | hypothetical protein |
| 5375574 | 5390150 | | 14576 | | DN1_orf06979 | hypothetical protein |
| 5375574 | 5390150 | | 14576 | | DN1_orf06981 | hypothetical protein |
| 5375574 | 5390150 | | 14576 | | DN1_orf06982 | single-stranded DNA-binding protein |
| 5375574 | 5390150 | | 14576 | | DN1_orf06983 | hypothetical protein |
| 5375574 | 5390150 | | 14576 | | DN1_orf06984 | plasmid DNA topoisomerase I |
| 5375574 | 5390150 | | 14576 | | DN1_orf06985 | hypothetical protein |
| 5375574 | 5390150 | | 14576 | | DN1_orf06986 | hypothetical protein |
| 5375574 | 5390150 | | 14576 | | DN1_orf06987 | hypothetical protein |
| 5401375 | 5427763 | | 26388 | | DN1_orf06999 | Tfp pilus assembly protein ATPase PilU |
| 5401375 | 5427763 | | 26388 | | DN1_orf07000 | type IV pilus protein PilV |
| 5401375 | 5427763 | | 26388 | | DN1_orf07001 | type IV B pilus protein |
| 5401375 | 5427763 | | 26388 | | DN1_orf07002 | putative transposase |
| 5401375 | 5427763 | | 26388 | | DN1_orf07003 | hypothetical protein |
| 5401375 | 5427763 | | 26388 | | DN1_orf07004 | hypothetical protein |
| 5401375 | 5427763 | | 26388 | | DN1_orf07005 | hypothetical protein |
| 5401375 | 5427763 | | 26388 | | DN1_orf07006 | hypothetical protein |
| 5401375 | 5427763 | | 26388 | | DN1_orf07007 | hypothetical protein |
| 5401375 | 5427763 | | 26388 | | DN1_orf07008 | hypothetical protein |
| 5401375 | 5427763 | | 26388 | | DN1_orf07009 | hypothetical protein |
| 5401375 | 5427763 | | 26388 | | DN1_orf07010 | hypothetical protein |
| 5401375 | 5427763 | | 26388 | | DN1_orf07012 | hypothetical protein |
| 5401375 | 5427763 | | 26388 | | DN1_orf07013 | hypothetical protein |
| 5401375 | 5427763 | | 26388 | | DN1_orf07014 | class III aminotransferase |
| 5401375 | 5427763 | | 26388 | | DN1_orf07015 | transposase, IS4 family protein |
| 5401375 | 5427763 | | 26388 | | DN1_orf07016 | hypothetical protein |
| 5401375 | 5427763 | | 26388 | | DN1_orf07017 | transposase |
| 5401375 | 5427763 | | 26388 | | DN1_orf07018 | conserved hypothetical protein |
| 5401375 | 5427763 | | 26388 | | DN1_orf07019 | hypothetical protein |
| 5401375 | 5427763 | | 26388 | | DN1_orf07020 | hypothetical protein |
| 5401375 | 5427763 | | 26388 | | DN1_orf07021 | hypothetical protein |
| 5401375 | 5427763 | | 26388 | | DN1_orf07022 | hypothetical protein |
| 5447362 | 5453405 | | 6043 | | DN1_orf07042 | hypothetical protein |
| 5447362 | 5453405 | | 6043 | | DN1_orf07043 | hypothetical protein |
| 5447362 | 5453405 | | 6043 | | DN1_orf07045 | hypothetical protein |
| 5631650 | 5639824 | | 8174 | | DN1_orf07265 | paraquat-inducible protein |
| 5631650 | 5639824 | | 8174 | | DN1_orf07266 | putative sulfite oxidase subunit YedZ |
| 5631650 | 5639824 | | 8174 | | DN1_orf07267 | putative sulfite oxidase subunit YedY |
| 5699905 | 5704102 | | 4197 | | DN1_orf07341 | hypothetical protein |
| 5699905 | 5704102 | | 4197 | | DN1_orf07342 | triosephosphate isomerase |
| 5699905 | 5704102 | | 4197 | | DN1_orf07344 | phosphoglucosamine mutase |
| 5699905 | 5704102 | | 4197 | | DN1_orf07345 | dihydropteroate synthase |
| 6303213 | 6307684 | | 4471 | | DN1_orf08114 | hypothetical protein |
| 6303213 | 6307684 | | 4471 | | DN1_orf08115 | hypothetical protein |
| 6303213 | 6307684 | | 4471 | | DN1_orf08117 | hypothetical protein |
| Plasmid | | | | |  |  |
| 273903 | 279751 | | 5848 | | DN1_orf00615 | hypothetical protein |
| 273903 | 279751 | | 5848 | | DN1_orf00616 | hypothetical protein |
| 273903 | 279751 | | 5848 | | DN1_orf00618 | hypothetical protein |
| 273903 | 279751 | | 5848 | | DN1_orf00619 | hypothetical protein |
| 273903 | 279751 | | 5848 | | DN1_orf00620 | hypothetical protein |
| 273903 | 279751 | | 5848 | | DN1_orf00621 | hypothetical protein |
| 273903 | 279751 | | 5848 | | DN1_orf00622 | hypothetical protein |
