# Supplementary material for: Genome Sequence and Metabolic Analysis of a Fluoranthene-Degrading Strain Pseudomonas aeruginosa DN1
Source: Front Microbiol. 2018 Oct 31;9:2595. doi: 10.3389/fmicb.2018.02595 (PMC6220107; doi:10.3389/fmicb.2018.02595)
Supplement: Supplementary file 12 [file Table_12.DOCX]

**Table S12 |** **Genes involved in biosurfactant rhamnolipid synthesis and regulation**

| **Start** | **End** | **Size** | **Locus_DN1_** | **Product Name** |
| --- | --- | --- | --- | --- |
| 2798792 | 2799679 | 295 | orf03616 | rhamnosyltransferase chain A (RhlA), |
| 3248318 | 3249889 | 523 | orf04168 | ATP-dependent RNA helicase RhlB, |
| 2797446 | 2798726 | 426 | orf03615 | Rhamnosyltransferase chain B (RhlC) |
| 2701360 | 2702130 | 256 | orf03474 | beta-ketoacyl reductase (RhlG) |
| 2796596 | 2797300 | 234 | orf03614 | transcriptional regulator RhlR |
| 6367953 | 6370559 | 868 | orf08205 | phosphomannomutase （algC） |
| 6188130 | 6189011 | 293 | orf07964 | glucose-1-phosphate thymidylyltransferase (RmlA) |
| 6186182 | 6187228 | 348 | orf07960 | dTDP-D-glucose 4,6-dehydratase (RmlB)： |
| 6189011 | 6189556 | 181 | orf07965 | dTDP-4-dehydrorhamnose 3,5-epimerase (RmlC) |
| 6187225 | 6188133 | 302 | orf07963 | dTDP-4-dehydrorhamnose reductase (RmlD) |
| 254968 | 256173 | 401 | orf00327 | 3-ketoacyl-CoA thiolase（FadA） |
| 2284846 | 2286993 | 715 | orf02955 | multifunctional fatty acid oxidation complex subunit alpha (FadB) |
| 2599126 | 2600814 | 562 | orf03340 | long-chain-fatty-acid-CoA ligase (FadD) |
| / | / | / | orf00759、orf07690etc. | acyl-CoA dehydrogenase (FadE) |
| 3779134 | 3779931 | 265 | orf04875 | NADH-dependent enoyl-ACP reductase (FadI) : |
| 2973463 | 2973903 | 146 | orf03839 | (3R)-hydroxymyristoyl-ACP dehydratase (FadZ) |
| 4648211 | 4649209 | 332 | orf06061 | MvfR |
| 1269987 | 1270925 | 312 | orf01671 | transcriptional regulator PtxR |
| 6299623 | 6300369 | 248 | orf08111 | AlgR |
| 4779854 | 4780039 | 61 | orf06212 | RsmA |
