# Supplementary material for: Genome Sequence and Metabolic Analysis of a Fluoranthene-Degrading Strain Pseudomonas aeruginosa DN1
Source: Front Microbiol. 2018 Oct 31;9:2595. doi: 10.3389/fmicb.2018.02595 (PMC6220107; doi:10.3389/fmicb.2018.02595)
Supplement: Supplementary file 13 [file Table_13.DOCX]

**Table S13 | Partial genes involved in PAHs degradation**

| **Locus Tag** | **Genomic location** | **Gene Product Name** |
| --- | --- | --- |
| **Region I** |  |  |
| DN1_orf00224 | 181326-182045 | Protocatechuate 3,4-dioxygenase, beta subunit |
| DN1_orf00226 | 182056-182661 | protocatechuate 3,4-dioxygenase, alpha subunit |
| DN1_orf00325 | 253341-254192 | glutaconate CoA-transferase, subunit A |
| DN1_orf00326 | 254189-254971 | glutaconate CoA-transferase, subunit B |
| DN1_orf00327 | 254968-256173 | 3-oxoadipyl-CoA thiolase |
| DN1_orf00331 | 257644-259023 | 3-carboxy-cis,cis-muconate cycloisomerase |
| DN1_orf00333 | 259038-259829 | 3-oxoadipate enol-lactonase |
| DN1_orf00334 | 259840-260241 | 4-carboxymuconolactone decarboxylase |
| DN1_orf00357 | 277085-278269 | p-hydroxybenzoate 3-monooxygenase |
| **Region II** |  |  |
| DN1_orf01207 | 919361-919732 | 5-carboxymethyl-2-hydroxymuconate isomerase |
| DN1_orf01267 | 956966-958147 | acetyl-CoA C-acetyltransferase |
| DN1_orf01278 | 965393-966031 | maleylacetoacetate isomerase |
| DN1_orf01280 | 966028-967326 | fumarylacetoacetase |
| DN1_orf01281 | 967331-968629 | homogentisate 1,2-dioxygenase |
| DN1_orf01400 | 1057811-1059085 | benzoate/toluate 1,2-dioxygenase subunit alpha |
| **Region III** |  |  |
| DN1_orf02006 | 1570759-1571403 | maleylacetoacetate isomerase |
| DN1_orf02054 | 1604194-1605126 | catechol 1,2-dioxygenase |
| DN1_orf02055 | 1605171-1605461 | muconolactone D-isomerase |
| DN1_orf02056 | 1605493-1606602 | muconate cycloisomerase |
| DN1_orf02061 | 1608975-1610369 | anthranilate 1,2-dioxygenase large subunit |
| DN1_orf02063 | 1610366-1610857 | anthranilate 1,2-dioxygenase small subunit |
| DN1_orf02064 | 1610876-1611898 | anthranilate 1,2-dioxygenase reductase component |
| DN1_orf02066 | 1611904-1612665 | dihydroxycyclohexadiene carboxylate dehydrogenase |
| DN1_orf02068 | 1612691-1613704 | benzoate/toluate 1,2-dioxygenase reductase component |
| DN1_orf02070 | 1613738-1614226 | benzoate/toluate 1,2-dioxygenase subunit beta |
| DN1_orf02071 | 1614223-1615590 | benzoate/toluate 1,2-dioxygenase subunit alpha |
| DN1_orf02135 | 1664859-1666049 | acetyl-CoA C-acetyltransferase |
| DN1_orf02456 | 1916005-1917246 | carboxymethylenebutenolidase |
| **Region IV** |  |  |
| DN1_orf02955 | 2284846-2286993 | 3-hydroxyacyl-CoA dehydrogenase |
| DN1_orf03334 | 2593261-2594649 | alkaline phosphatase |
| DN1_orf03443 | 2681194-2682234 | amidase |
| DN1_orf03536 | 2744584-2745354 | enoyl-CoA hydratase |
| DN1_orf03763 | 2912477-2913682 | acetyl-CoA C-acetyltransferase |
| DN1_orf03764 | 2913705-2915237 | 3-hydroxybutyryl-CoA dehydrogenase |
| DN1_orf03819 | 2954380-2955492 | S-(hydroxymethyl)glutathione dehydrogenase |
| **Region V** |  |  |
| DN1_orf04482 | 3474080-3475567 | phenylacetaldehyde dehydrogenase |
| DN1_orf04505 | 3496690-3498255 | 4-hydroxyphenylacetate 3-monooxygenase |
| DN1_orf04506 | 3498285-3498797 | flavin reductase (NADH) |
| DN1_orf04509 | 3500603-3501136 | 2,4'-dihydroxyacetophenone dioxygenase |
| DN1_orf04543 | 3527853-3528512 | 5-oxopent-3-ene-1,2,5-tricarboxylate decarboxylase |
| DN1_orf04544 | 3528523-3529302 | 5-oxopent-3-ene-1,2,5-tricarboxylate decarboxylase |
| DN1_orf04545 | 3529299-3530759 | 5-carboxymethyl-2-hydroxymuconic-semialdehyde dehydrogenase |
| DN1_orf04547 | 3530891-3531814 | 3,4-dihydroxyphenylacetate 2,3-dioxygenase |
| DN1_orf04548 | 3531825-3532217 | 5-carboxymethyl-2-hydroxymuconate isomerase |
| DN1_orf04602 | 3575908-3577617 | amidase |
| DN1_orf04851 | 3760181-3760993 | enoyl-CoA hydratase |
| DN1_orf04959 | 3851141-3851830 | enoyl-CoA hydratase |
| DN1_orf04976 | 3858949-3861093 | 3-hydroxyacyl-CoA dehydrogenase |
| DN1_orf04977 | 3861107-3862312 | acetyl-CoA C-acetyltransferase |
| DN1_orf05136 | 3968646-3970175 | 3-hydroxybutyryl-CoA dehydrogenase |
| **Region VI** |  |  |
| DN1_orf06113 | 4705323-4706552 | salicylate hydroxylase |
| DN1_orf06125 | 4717154-4717600 | 4-hydroxybenzoyl-CoA thioesterase |
| DN1_orf06141 | 4730286-4730546 | acylphosphatase |
| DN1_orf06493 | 4984384-4985778 | amidase |
| DN1_orf06649 | 5116031-5117512 | amidase |
| DN1_orf06877 | 5292694-5293080 | 4-carboxymuconolactone decarboxylase |
| DN1_orf07396 | 5740987-5742264 | acetyl-CoA C-acetyltransferase |
| DN1_orf07573 | 5872929-5874527 | benzoylformate decarboxylase |
| DN1_orf07578 | 5877239-5878294 | vanillate monooxygenase |
| DN1_orf07579 | 5878309-5879262 | vanillate monooxygenase ferredoxin subunit |
| DN1_orf08354 | 6483994-6485022 | alcohol dehydrogenase, propanol-preferring |
