# Supplementary material for: Genome Sequence and Metabolic Analysis of a Fluoranthene-Degrading Strain Pseudomonas aeruginosa DN1
Source: Front Microbiol. 2018 Oct 31;9:2595. doi: 10.3389/fmicb.2018.02595 (PMC6220107; doi:10.3389/fmicb.2018.02595)
Supplement: Supplementary file 14 [file Table_14.DOCX]

**Table S14︱Genes involved in the citrate acid (TCA) cycle**

| **Locus_DN1_** | **EC** |  | **Product Name** |
| --- | --- | --- | --- |
| orf05206 | 2.3.3.1 | K01647 | citrate synthase |
| orf05235 | 4.2.1.3 | K01681 | aconitate hydratase |
| orf06380 | 4.2.1.3 | K01681 | aconitate hydratase |
| orf04903 | 4.2.1.3 | K01682 | aconitate hydratase 2 / 2-methylisocitrate dehydratase |
| orf04903 | 4.2.1.99 | K01682 | aconitate hydratase 2 / 2-methylisocitrate dehydratase |
| orf02369 | 1.1.1.42 | K00031 | isocitrate dehydrogenase |
| orf02370 | 1.1.1.42 | K00031 | isocitrate dehydrogenase |
| orf05200 | 1.2.4.2 | K00164 | 2-oxoglutarate dehydrogenase E1 component |
| orf05198 | 2.3.1.61 | K00658 | 2-oxoglutarate dehydrogenase E2 component (dihydrolipoamide succinyltransferase) |
| orf01659 | 1.8.1.4 | K00382 | dihydrolipoamide dehydrogenase |
| orf05195 | 1.8.1.4 | K00382 | dihydrolipoamide dehydrogenase |
| orf07468 | 1.8.1.4 | K00382 | dihydrolipoamide dehydrogenase |
| orf05193 | 6.2.1.5 | K01902 | succinyl-CoA synthetase alpha subunit |
| orf05194 | 6.2.1.5 | K01903 | succinyl-CoA synthetase beta subunit |
| orf08389 | 2.8.3.18 | K18118 | succinyl-CoA:acetate CoA-transferase |
| orf05202 | 1.3.5.1 | K00239 | succinate dehydrogenase / fumarate reductase, flavoprotein subunit |
| orf05202 | 1.3.5.4 | K00239 | succinate dehydrogenase / fumarate reductase, flavoprotein subunit |
| orf05201 | 1.3.5.1 | K00240 | succinate dehydrogenase / fumarate reductase, iron-sulfur subunit |
| orf05201 | 1.3.5.4 | K00240 | succinate dehydrogenase / fumarate reductase, iron-sulfur subunit |
| orf05205 | \N | K00241 | succinate dehydrogenase / fumarate reductase, cytochrome b subunit |
| orf05203 | \N | K00242 | succinate dehydrogenase / fumarate reductase, membrane anchor subunit |
| orf06629 | 4.2.1.2 | K01676 | fumarate hydratase, class I |
| orf06289 | 4.2.1.2 | K01679 | fumarate hydratase, class II |
| orf06856 | 4.2.1.2 | K01679 | fumarate hydratase, class II |
| orf03563 | 1.1.5.4 | K00116 | malate dehydrogenase (quinone) |
| orf07198 | 1.1.5.4 | K00116 | malate dehydrogenase (quinone) |
| orf08369 | 6.4.1.1 | K01959 | pyruvate carboxylase subunit A |
| orf08366 | 6.4.1.1 | K01960 | pyruvate carboxylase subunit B |
| orf08009 | 4.1.1.49 | K01610 | phosphoenolpyruvate carboxykinase (ATP) |
| orf07744 | 1.2.4.1 | K00163 | pyruvate dehydrogenase E1 component |
| orf03521 | 1.2.4.1 | K00161 | pyruvate dehydrogenase E1 component alpha subunit |
| orf04587 | 1.2.4.1 | K00161 | pyruvate dehydrogenase E1 component alpha subunit |
| orf03519 | 1.2.4.1 | K00162 | pyruvate dehydrogenase E1 component beta subunit |
| orf04588 | 1.2.4.1 | K00162 | pyruvate dehydrogenase E1 component beta subunit |
| orf03518 | 2.3.1.12 | K00627 | pyruvate dehydrogenase E2 component (dihydrolipoamide acetyltransferase) |
| orf04589 | 2.3.1.12 | K00627 | pyruvate dehydrogenase E2 component (dihydrolipoamide acetyltransferase) |
| orf07747 | 2.3.1.12 | K00627 | pyruvate dehydrogenase E2 component (dihydrolipoamide acetyltransferase) |
