# Supplementary figures and images for: Genome Sequence and Metabolic Analysis of a Fluoranthene-Degrading Strain Pseudomonas aeruginosa DN1
Source: Front Microbiol. 2018 Oct 31;9:2595. doi: 10.3389/fmicb.2018.02595 (PMC6220107; doi:10.3389/fmicb.2018.02595)

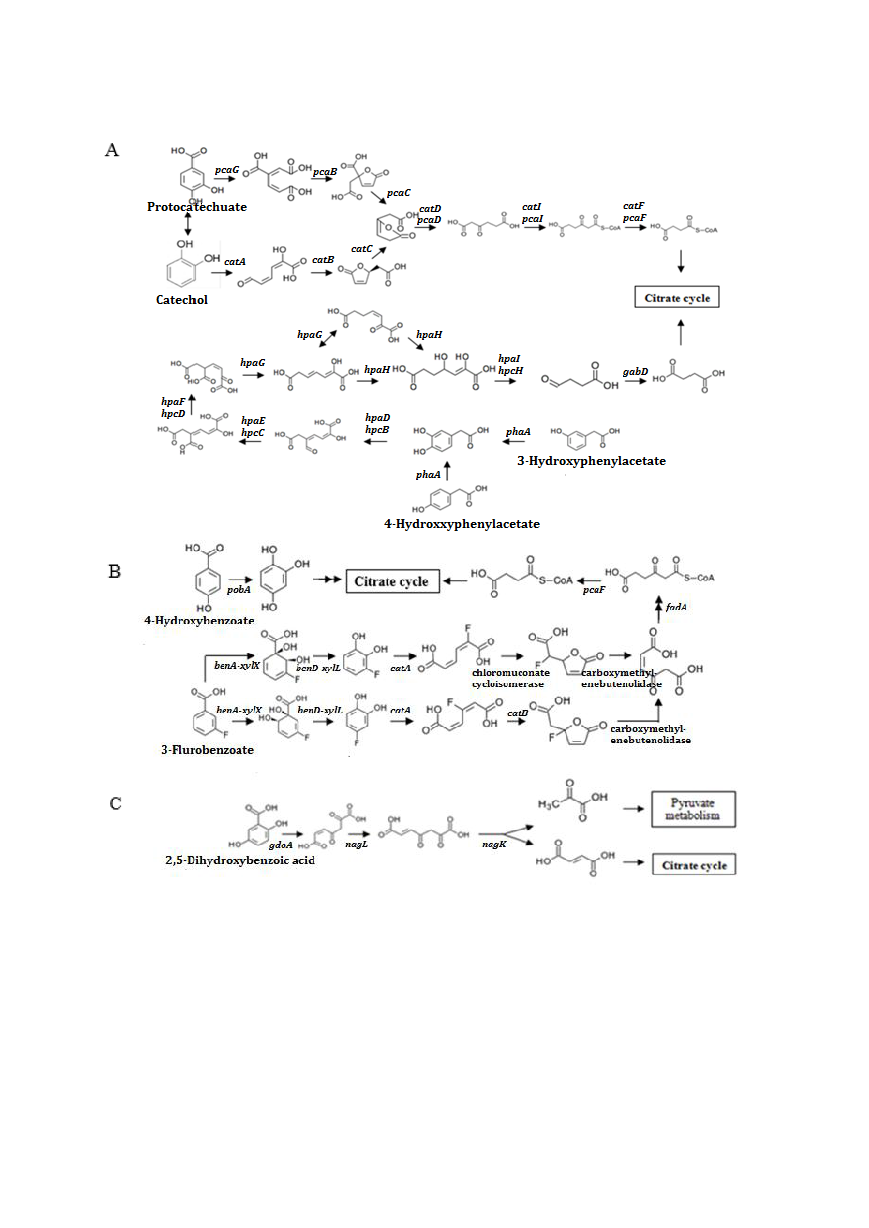

Supplement: Supplementary file 15 [file Image_1.TIF]

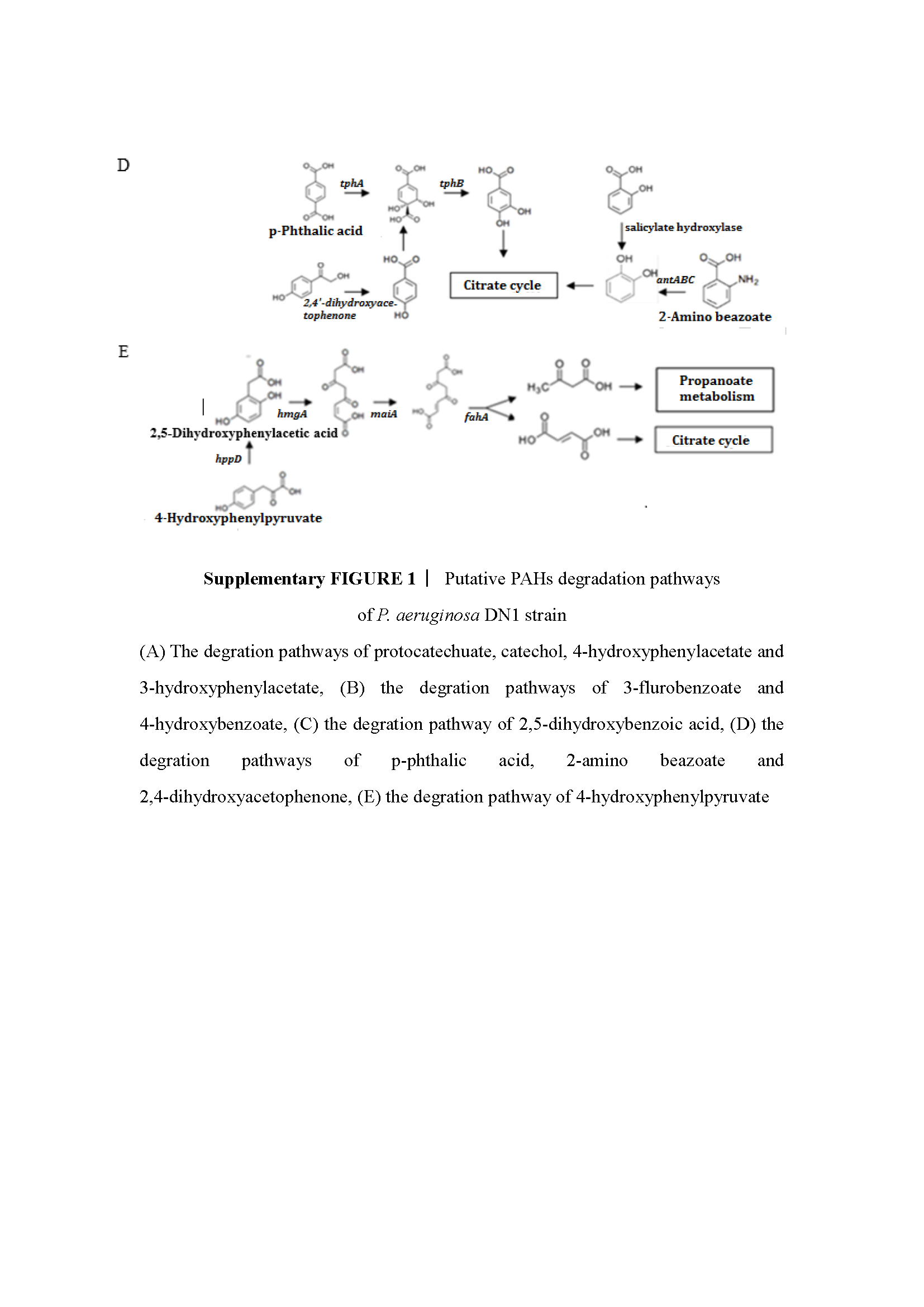

Supplement: Supplementary file 16 [file Image_2.TIFF]
